# Supplementary material for: Oxa-Iboga alkaloids lack cardiac risk and disrupt opioid use in animal models
Source: Nat Commun. 2024 Sep 20;15:8118. doi: 10.1038/s41467-024-51856-y (PMC11415492; doi:10.1038/s41467-024-51856-y)
Supplement: Supplementary file 1 — Supplementary Information [file 41467_2024_51856_MOESM1_ESM.pdf]

Supplementary Information:

Oxa-Iboga Alkaloids Lack Cardiac Risk and Disrupt Opioid Use in Animal Models

(57 pages)

Contents

Fig. S1 | Synthesis of oxa-iboga analog intermediates. 3

Fig. S2 | Synthesis of oxa-iboga analogs. 4

Fig. S3 | Characterization of noribogaine and oxa-iboga analogs target profiles. 6

Fig. S4 | Opioid receptor signaling induced by noribogaine and oxa-iboga analogs (part 1). 7

Fig. S5 | Opioid receptor signaling induced by noribogaine and oxa-iboga analogs (part 2). 9

Fig. S6 | BRET assay for activation of G protein heterotrimers (TRUPATH). 10

Fig. S8 | Pro-arrhythmia risk of noribogaine and oxa-iboga analogs examined in human primary cardiomyocytes. 12

Fig. S9 | Morphine self-administration studies in rats. 13

Table S1. In vitro data collected for iboga alkaloids and analogs. 14

Table S2. Summary of behavioral (in vivo) and pharmacokinetic (in vivo, ex vivo detection) data for oxa-iboga analogs. 16

Synthetic procedures and compound characterization 18

- Noribogaine hydrochloride 18
- Synthesis of exo/endo-isoquinuclidine ketone intermediates 2a and 2b 18
- Synthesis of exo/endo-isoquinuclidine tosylhydrazones 3a and 3b 19
- exo-Methyl 7-(1-(2-tosylhydrazono)ethyl)-2-azabicyclo[2.2.2]oct-5-ene-2-carboxylate (3a) 19
- endo-Methyl 7-(1-(2-tosylhydrazono)ethyl)-2-azabicyclo[2.2.2]oct-5-ene-2-carboxylate (3b) 19
- exo-Methyl 7-ethyl-2-azabicyclo[2.2.2]oct-5-ene-2-carboxylate (4a) 19
- endo-Methyl 7-ethyl-2-azabicyclo[2.2.2]oct-5-ene-2-carboxylate (4b) 20
- 5-Methoxybenzofuran-3(2H)-one (5) 20
- Ethyl 2-(5-methoxybenzofuran-3-yl)acetate (6a) 20
- 2-(5-methoxybenzofuran-3-yl)acetic acid (6b) 21
- 2-(5-Methoxybenzofuran-3-yl)ethanol (7) 21
- 3-(2-Bromoethyl)-5-methoxybenzofuran (8) 22
- General Procedure for Preparation of N-benzofuranylethylisoquinuclidines 22
- exo-7-Ethyl-2-(2-(5-methoxybenzofuran-3-yl)ethyl)-2-azabicyclo[2.2.2]oct-5-ene (9a) 22
- endo-7-Ethyl-2-(2-(5-methoxybenzofuran-3-yl)ethyl)-2-azabicyclo[2.2.2]oct-5-ene (9b) 23
- General Procedure for Preparation of Oxa-ibogaine Analogs by Ni(0)-catalyzed Cyclization 23
- Oxa-ibogaine (10a) 23
- Epi-oxa-ibogaine (10b) 24

General Procedure for Preparation of Oxa-noribogaine Analogs by Demethylation 24
rac-oxa-noribogaine (Oxa-noriboga, 11a) 24
rac-epi-oxa-noribogaine (Epi-oxa, 11b) 26
General Procedure for Preparation of Oxa-ibogaine Analogs by lithiation/iodination and reductive Heck sequence. 26
Oxa-ibogaine (10a) 27
Epi-oxa-ibogaine (10b) 27
Synthesis Comments 27
X-Ray structure determination of Oxa-ibogaine 10a 28
Appendix 1. High Resolution Mass Spectra 29
Appendix 2. NMR Spectra 33
Supplementary References 57

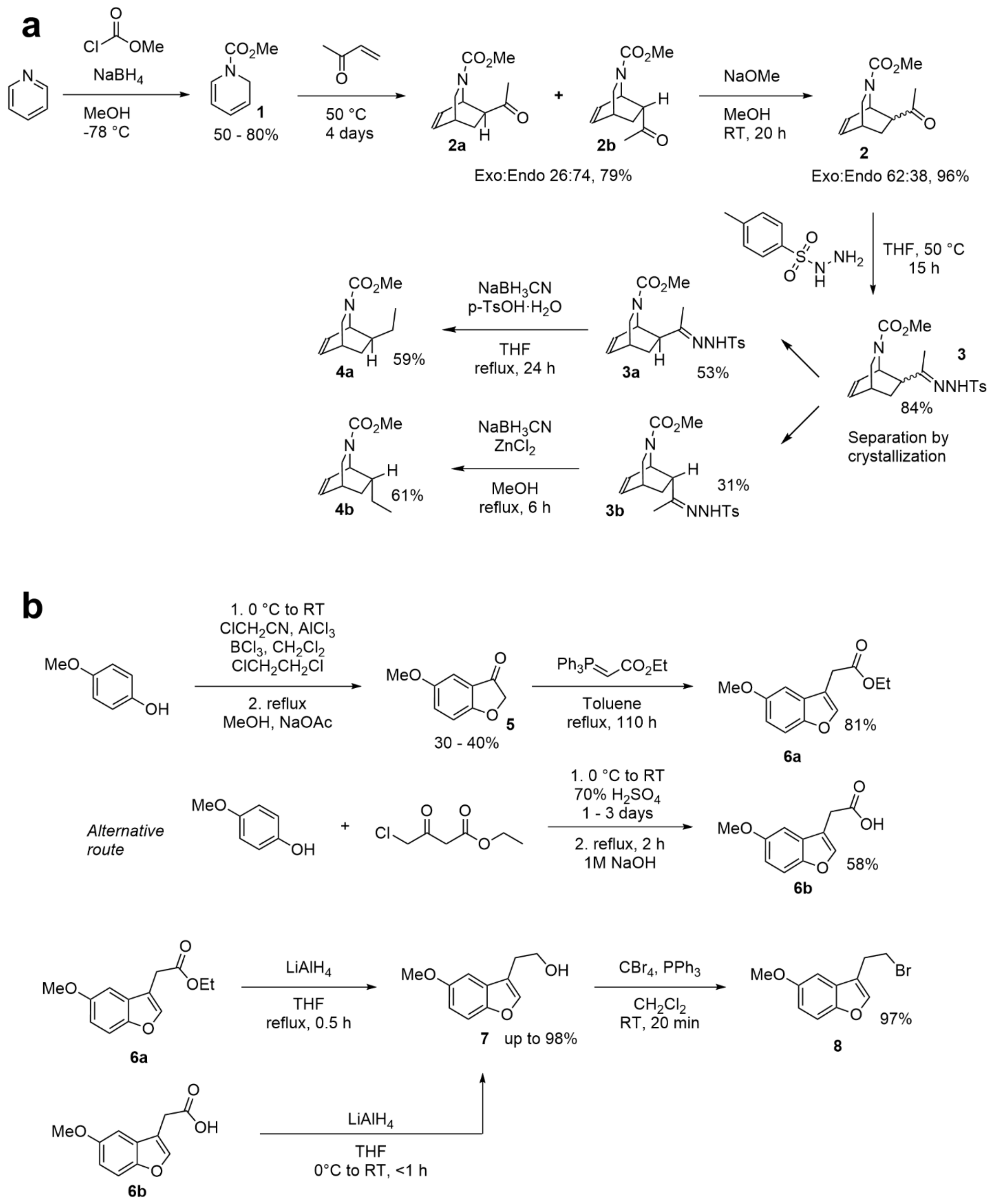

Fig. S1 | Synthesis of oxa-iboga analog intermediates. a The isoquinuclidine ring was assembled via a Diels-Alder reaction starting from dihydropyridine **1**. **b** The benzofuran intermediate was prepared using two slightly different reaction pathways, with the alternative route proving to be a more convenient choice for a multigram synthesis.

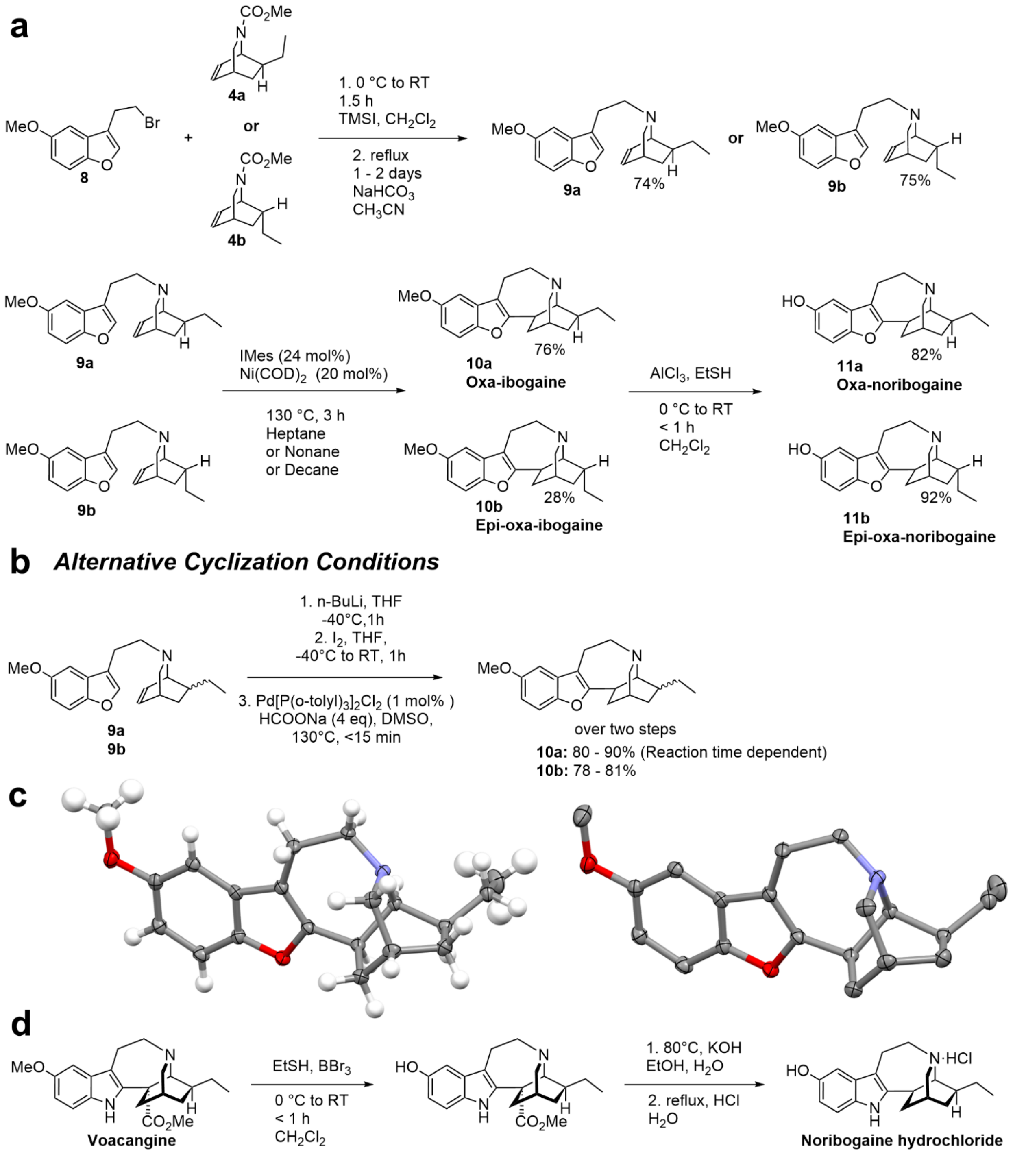

Fig. S2 | Synthesis of oxa-iboga analogs. a Formation of the 7-membered azepine ring can be achieved using a nickel-catalyzed coupling between the benzofuran and isoquinuclidine moieties or **b** via a sequence of lithiation/iodination followed by reductive Heck coupling. **c** An X-ray crystallography structure of racemic oxa-ibogaine (only one enantiomer shown) in thermal ellipsoid style (50% probability). Atom color coding: H white, C gray, O red, N blue. **d** Noribogaine hydrochloride used in the study was prepared starting from Voacangine isolated from *Voacanga africana* root bark according to a procedure which avoided using ibogaine as a synthetic intermediate.

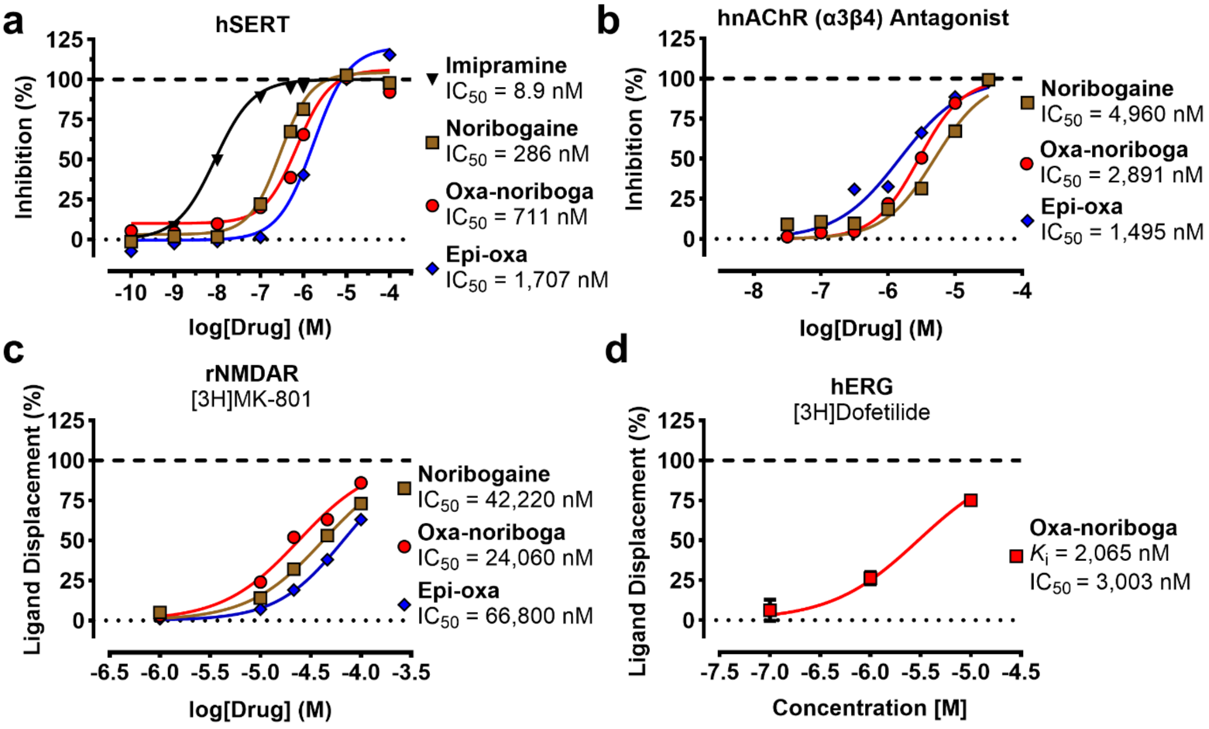

**e**	**Broad receptor radioligand displacement screen for oxa-noribogaine.**		
Primary Binding Screen at 10 μM	Secondary Screen (K_i ± SEM)		
5-HT1A, 5-HT1B, 5-HT1D, 5-HT1E, 5-HT2A, 5-HT2B, 5-HT2C, 5-HT3, 5-HT4, 5-HT5A, 5-HT6, 5-HT7A, A1, A2A, α1A, α1B, α1D, α2A, α2B, α2C, β1, β2, β3, BZP, D1, D2, D3, D4, D5, DAT, DOR, GABAA, H1, H2, H3, H4, KOR, M1, M2, M3, M4, M5, MOR, NET, PBR,SERT, σ1, σ2, AMPA, NR2B, M4 D, NTS1, mGluR5, α2β2, α2β4, α3β2, α3β4, α4β2, α4β2 (Rat Brain), α4β4, α7, NOP, OT, V1A, V1B, V2, BZP Rat Brain Site	**α2A (n = 3)**	**α2C (n = 3)**	**α3β2 (n = 1)**
	1,452 ± 538	2,435 ± 476	~2,207
	**α3β4 (n = 1)**	**α4β4 (n = 1)**	**α7 (n = 1)**
	~2,802	~3,485	~1,293
	**(σ2 (n = 3)**	**SERT (n = 3)**	**D5 (n = 3)**
	4,488 ± 358	1,157 ± 391	4,217 ± 866
	**DOR (n = 3)**	**KOR (n = 3)**	**MOR (n = 3)**
	411 ± 30	1.6 ± 0.3	56 ± 15

**f**	**Functional assays [EC_{50}/IC_{50}, (95% CI) in nM], {%E_{max}} (n = 1).**		
Assay (Species)	Receptor	Noribogaine	Oxa-noribogaine
GTPγS (human)	hKOR (Agonist)	8,325 (6143; 11,280) {73}	/
	h5-HT_{2A} (Agonist)	n.d. {3% at 25 μM}	/
IP1 (human)	h5-HT_{2A} (Agonist)	n.d. {<0% at 25 μM}	n.d. {2% at 10 μM}
	h5-HT_{2B} (Agonist)	n.d. {<0% at 10 μM}	n.d. {<0% at 10 μM}
	h5-HT_{2B} (Antagonist)	n.d. {10% at 10 μM}	n.d. {<0% at 10 μM}

Fig. S3 | Characterization of noribogaine and oxa-iboga analogs target profiles. a Inhibition of human serotonin transporter (hSERT) by noribogaine and oxa-iboga analogs. Data are presented as mean (n = 3) ± SEM. **b** Inhibition of human nicotinic acetylcholine receptor (nAChR α3β4) by iboga alkaloids as determined by electrophysiological assays (n = 1). **c** Binding affinity (radioligand displacement assay, [^{3}H]MK-801) of iboga alkaloids at rat NMDA receptor (n = 1). **d** Oxa-noribogaine binds to the human ether-à-go-go-related gene (**hERG**) potassium channel with a low micromolar activity (radioligand displacement assay, [^{3}H]Dofetilide), (n = 1). **e** Radioligand displacement by oxa-noribogaine was tested for a broad selection of targets at 10 μM. Binding affinities [Ki ± SEM, nM] were determined only for targets identified in the primary binding screening as having ligand displacement >50% at 10 μM. Targets tested in the primary but not secondary screen showed minimal binding affinity at 10 μM. **f** Noribogaine is a low micromolar agonist of human KOR. No agonist activity at human 5-hydroxytryptamine type 2 receptors (h5-HT2R) was detected for noribogaine and oxa-noribogaine up to 10 μM (n = 1).

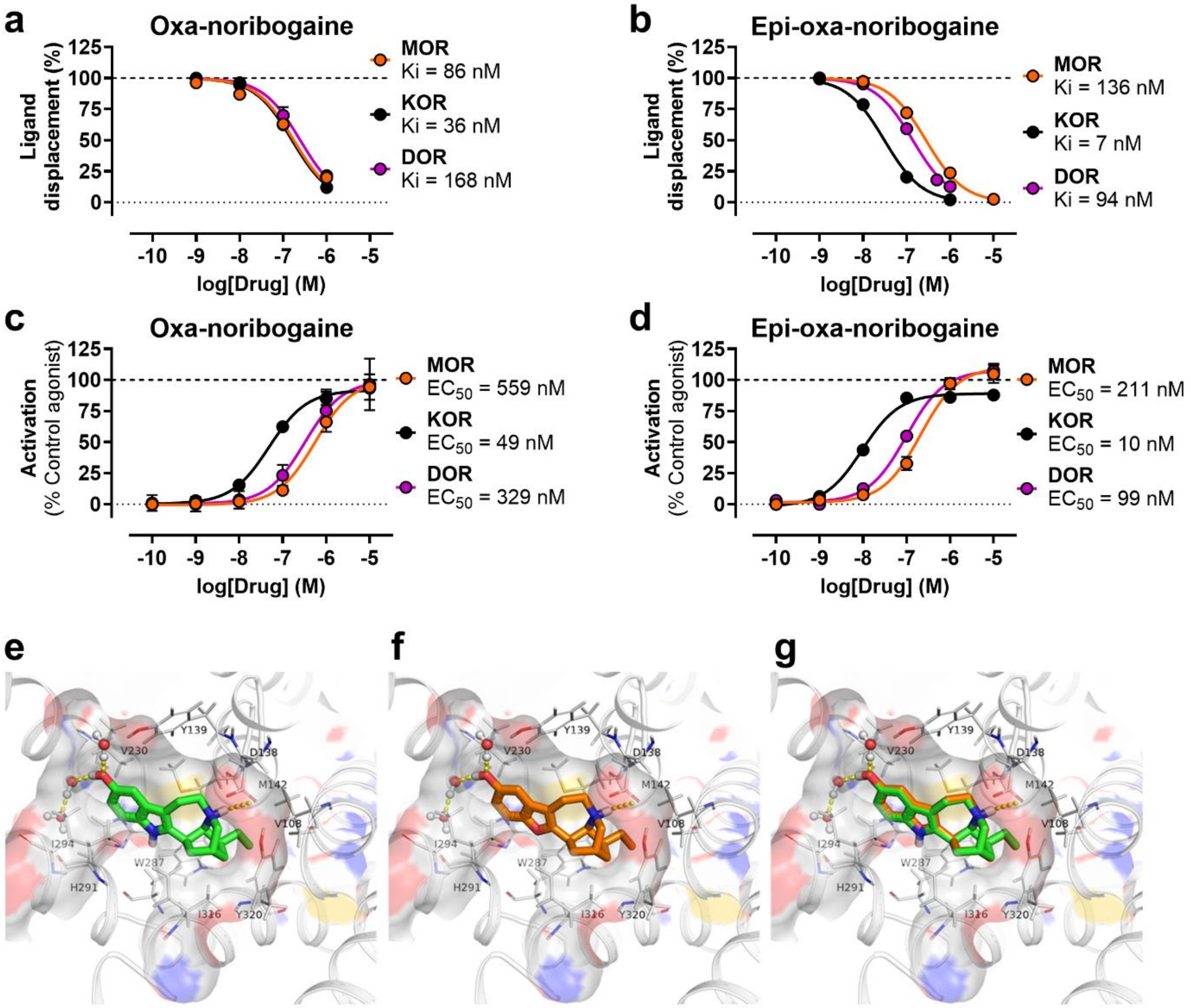

Fig. S4 | Opioid receptor signaling induced by noribogaine and oxa-iboga analogs (part 1). a Binding affinity measured by radioligand ([^{125}I]-*IBNtxA)* displacement experiments at mouse opioid receptors (mMOR, mKOR and mDOR) for oxa-noribogaine and **b** epi-oxa-noribogaine. Data are presented as mean (MOR n = 3, KOR/DOR n = 5) ± SEM. **c** Agonist activity at mouse opioid receptors determined by [^{35}S]GTPγS assay for oxa-noribogaine and **d** epi-oxa-noribogaine. Data are presented as mean (n = 3) ± SEM. Docking pose of noribogaine (**e,** carbon scaffold in green), oxa-noribogaine (**f,** carbon scaffold in orange) and superimposed overlay (**g**) in sticks representation inside active state KOR structure. Hydrogen bonding shown in yellow dots. However, the docking studies do not provide an explanation for the large differences in binding and signaling potencies between noribogaine and oxa-noribogaine as the docking poses are similar. We suggest the observed differences in binding potency are due to electronic effects, namely changes in the electron density distribution in the ligand aromatic ring and the resulting interactions with larger surface of the receptor.

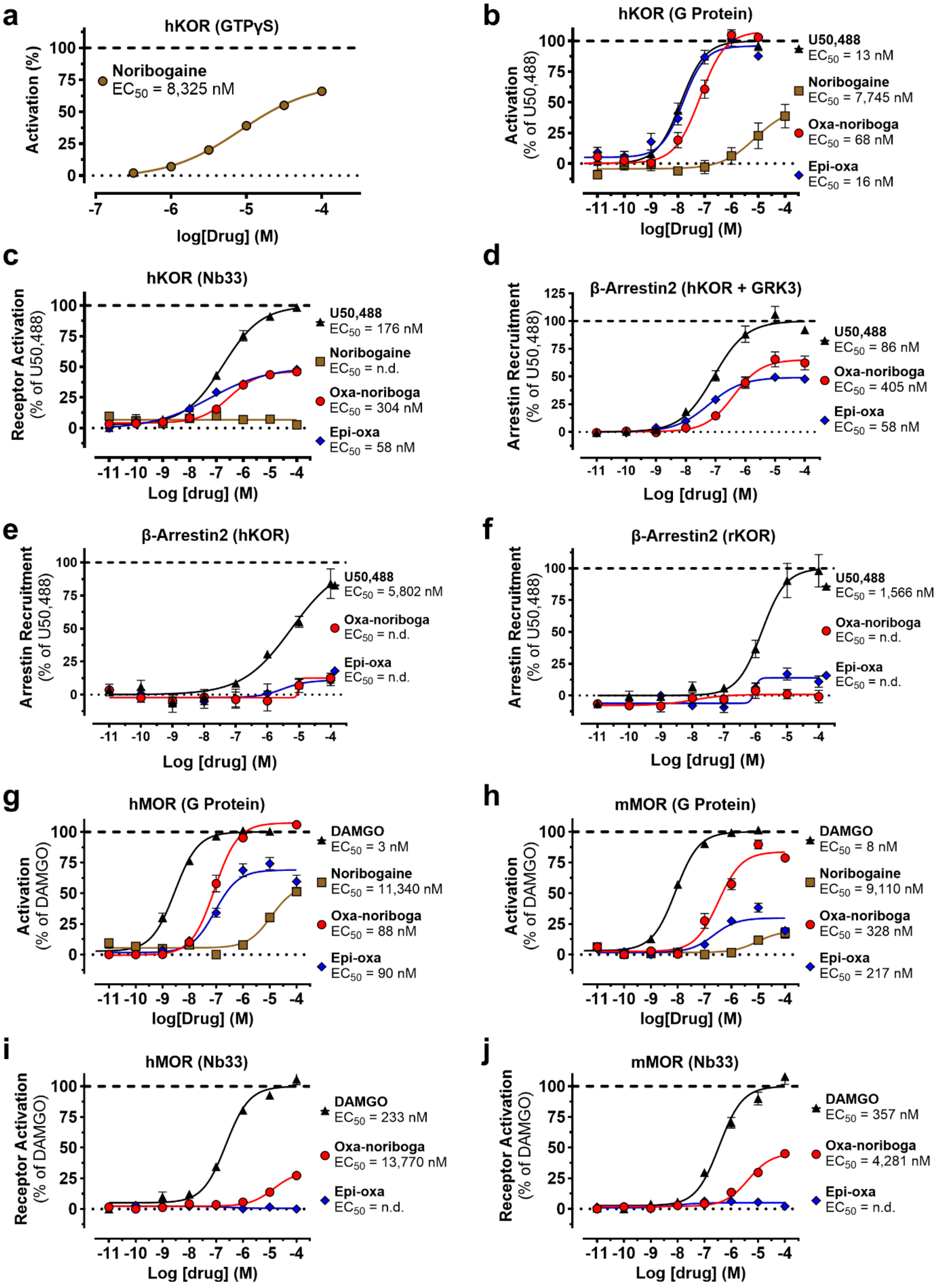
a
hKOR (GTPγS)
Activation (%)
Noriboogaine
EC50 = 8,325 nM
log[Drug] (M)
b
hKOR (G Protein)
Activation (% of U50,488)
U50,488 EC50 = 13 nM
Noribogaine EC50 = 7,745 nM
Oxa-noriboga EC50 = 68 nM
Epi-oxa EC50 = 16 nM
log[Drug] (M)
c
hKOR (Nb33)
Receptor Activation (% of U50,488)
U50,488 EC50 = 176 nM
Noribogaine EC50 = n.d.
Oxa-noriboga EC50 = 304 nM
Epi-oxa EC50 = 58 nM
Log [drug] (M)
d
β-Arrestin2 (hKOR + GRK3)
Arrestin Recruitment (% of U50,488)
U50,488 EC50 = 86 nM
Oxa-noriboga EC50 = 405 nM
Epi-oxa EC50 = 58 nM
Log [drug] (M)
e
β-Arrestin2 (hKOR)
Arrestin Recruitment (% of U50,488)
U50,488 EC50 = 5,802 nM
Oxa-noriboga EC50 = n.d.
Epi-oxa EC50 = n.d.
Log [drug] (M)
f
β-Arrestin2 (rKOR)
Arrestin Recruitment (% of U50,488)
U50,488 EC50 = 1,566 nM
Oxa-noriboga EC50 = n.d.
Epi-oxa EC50 = n.d.
Log [drug] (M)
g
hMOR (G Protein)
Activation (% of DAMGO)
DAMGO EC50 = 3 nM
Noribogaine EC50 = 11,340 nM
Oxa-noriboga EC50 = 88 nM
Epi-oxa EC50 = 90 nM
log[Drug] (M)
h
mMOR (G Protein)
Activation (% of DAMGO)
DAMGO EC50 = 8 nM
Noribogaine EC50 = 9,110 nM
Oxa-noriboga EC50 = 328 nM
Epi-oxa EC50 = 217 nM
log[Drug] (M)
i
hMOR (Nb33)
Receptor Activation (% of DAMGO)
DAMGO EC50 = 233 nM
Oxa-noriboga EC50 = 13,770 nM
Epi-oxa EC50 = n.d.
Log [drug] (M)
j
mMOR (Nb33)
Receptor Activation (% of DAMGO)
DAMGO EC50 = 357 nM
Oxa-noriboga EC50 = 4,281 nM
Epi-oxa EC50 = n.d.
Log [drug] (M)

Fig. S5 | Opioid receptor signaling induced by noribogaine and oxa-iboga analogs (part 2). a Agonist activity of noribogaine at human KOR determined by [^{35}S]GTPγS assay (n = 1). **b** Human KOR agonist activity (G protein BRET assay) for noribogaine and oxa-noribogaine analogs. **c** Human KOR agonist activity determined using a nanobody sensor (Nb33 BRET assay) for noribogaine and oxa-noribogaine analogs. **d** Oxa-iboga alkaloids recruit β-arrestin with partial efficacy compared to U50,488 in HEK-293 cells expressing human (hKOR) kappa opioid receptors. **e** Unamplified experiment without addition of GRK3 (G Protein-Coupled Receptor Kinase 3) determined at human (hKOR) and **f** rat receptors. **g** Oxa-noribogaine acts as a full agonist, while epi-oxa and noribogiane as partial/low efficacy agonists in the human and **h** mouse mu opioid receptor (MOR) G protein BRET assay. **i** Observed agonist activity of oxa-iboga analogs is significantly diminished in the human and **j** mouse MOR nanobody sensor-based assay. Data are presented as mean ± SEM (n = 3, all panels except **a**).

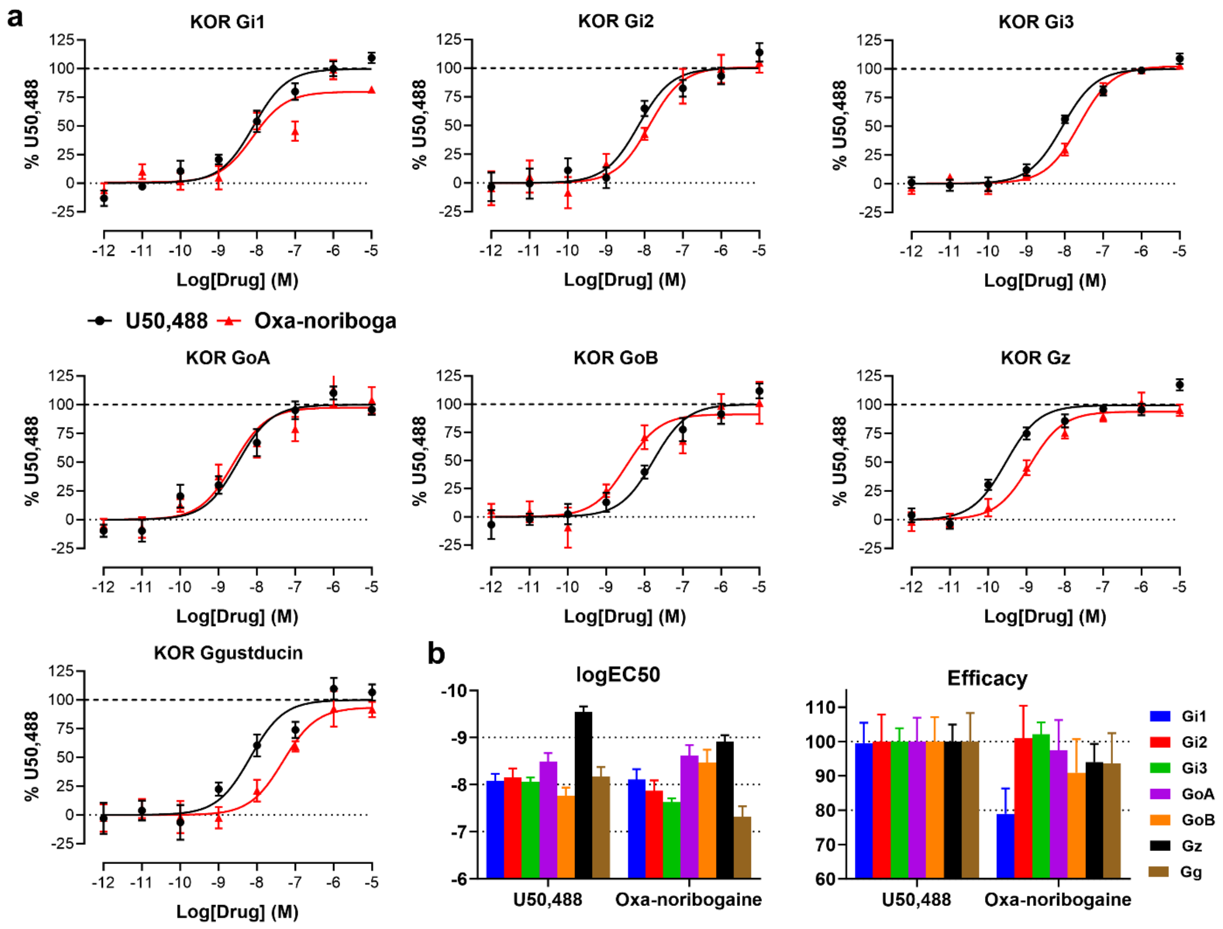

Fig. S6 | BRET assay for activation of G protein heterotrimers (TRUPATH). a Dose-response curves generated for G protein heterotrimer activation incorporating seven distinct Gα subunits. **b** Bar graph comparing the distinct signaling profiles of a classical, strongly aversive KOR agonist (U50,488) and an atypical one (oxa-noribogaine). Data are presented as mean ± SEM (n = 3 biological replicates).

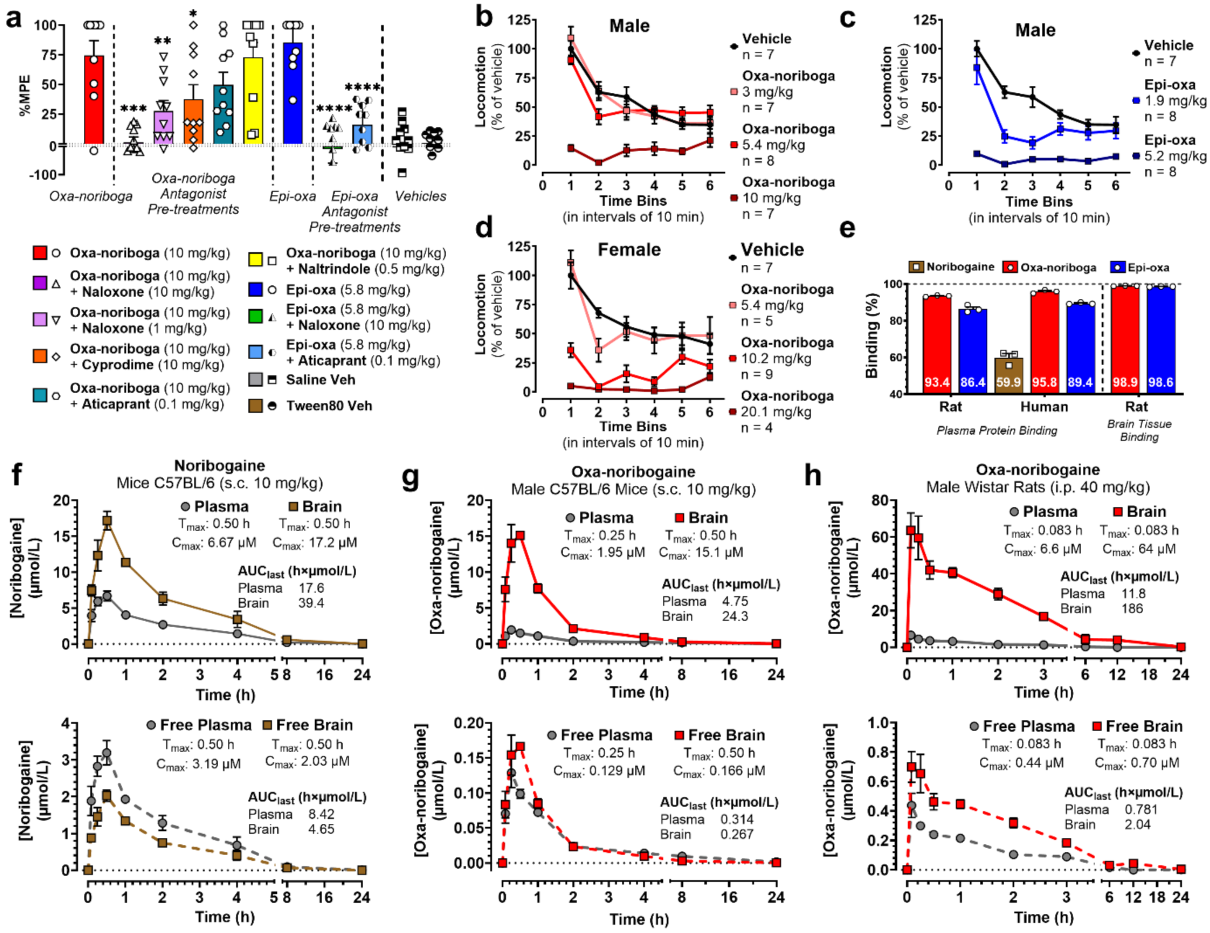

Fig. S7 | In vivo characterization of iboga analogs. a Effect of pre-treatment with antagonists naltrindole (DOR selective), naloxone (non-selective at 10 mg/kg, MOR preferring at 1 mg/kg), cyprodime (MOR selective) and aticaprant (KOR selective) on oxa-noribogaine induced nociception in male mice and naloxone (10 mg/kg) and aticaprant (0.1 mg/kg) on epi-oxa-noribogaine. Data are presented as mean (n = 10) ± SEM. **b** Escalating dose effect of oxa-iboga analogs on locomotion in OF test (male mice). Oxa-noribogaine (3.0 mg/kg = ED_{50}, 5.4 mg/kg = ED_{80} and 10 mg/kg > ED_{95}) and **c** epi-oxa-noribogaine (1.9 mg/kg = ED_{50} and 5.2 mg/kg = ED_{80}). **d**, Escalating dose effect of oxa-noribogaine (5.4 mg/kg = male ED_{80}, 10.2 mg/kg = female ED_{80} and 20.1 mg/kg > ED_{95}) on locomotion in OF test (female mice). Data are presented as mean ± SEM. **e** Rat and human plasma protein binding and rat brain tissue binding data of noribogaine and oxa-iboga analogs. Data are presented as mean (n = 3 technical replicates) ± SEM. **f** Noribogaine (s.c. 10 mg/kg) PK in male C57BL/6 mice, total and estimated free concentrations. **g** Oxa-noribogaine exhibits rapid and high brain penetration in PK (male C57BL/6 mice, s.c. 10 mg/kg, brain/plasma = 5.5). Approximately 85% of the compound is cleared in the first 2 hours and only traces remain after 8 hours. Estimated free plasma and brain drug concentrations. Assuming linearity of PK below 10 mg/kg, $C_{max(brain)}$ at an ED_{50} analgesic dose (3 mg/kg) is expected to be ~ 49 nM, which matches very well the in vitro KOR activation potency (EC_{50} = 41 nM in BRET assay, EC_{50} = 49 nM, in [^{35}S]GTPgS assay). **h** Oxa-noribogaine (i.p. 40 mg/kg) PK in male Wistar rats, total and estimated free concentrations. Rat plasma protein and brain tissue binding data were used to estimate free drug fractions in mice. The C_{max} and area under the curve (AUC, drug exposure) values for estimated free drugs are much smaller for oxa-noribogaine in comparison to noribogaine which is relevant for interpreting the oxa-noribogaine's superior efficacy in addiction models. Data are presented as mean (n = 3 animals/time point) ± SEM. Specifics of statistical tests are reported in source data file, **P* < 0.05, ***P* < 0.01, ****P* < 0.001.

a

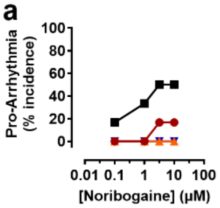

PRO-ARRHYTHMIA	Vehicle	0.1 μM	1 μM	3.16 μM	10 μM
Cell 1	*No*	*No*	*No*	*No*	*No*
Cell 2	*No*	*No*	*No*	*Yes*	*Yes*
Cell 3	*No*	*No*	*No*	*No*	*No*
Cell 4	*No*	*No*	*Yes*	*Yes*	*Yes*
Cell 5	*No*	*Yes*	*Yes*	*Yes*	*Yes*
Cell 6	*No*	*No*	*No*	*No*	*No*
Cumulative Percentage	0.00%	16.67%	33.33%	50.00%	50.00%

b

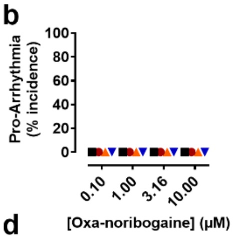

PRO-ARRHYTHMIA	Vehicle	0.1 μM	1 μM	3.16 μM	10 μM
Cell 1	*No*	*No*	*No*	*No*	*No*
Cell 2	*No*	*No*	*No*	*No*	*No*
Cell 3	*No*	*No*	*No*	*No*	*No*
Cell 4	*No*	*No*	*No*	*No*	*No*
Cell 5	*No*	*No*	*No*	*No*	*No*
Cumulative Percentage	0.00%	0.00%	0.00%	0.00%	0.00%

c

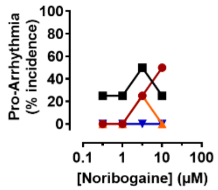

PRO-ARRHYTHMIA	Vehicle	0.316 μM	1 μM	3.16 μM	10 μM
Cell 1	*No*	*No*	*No*	*Yes*	*No*
Cell 2	*No*	*Yes*	*No*	*No*	*No*
Cell 3	*No*	*No*	*No*	*Yes*	*Yes*
Cell 4	*No*	*No*	*Yes*	*Yes*	*Yes*
Cumulative Percentage	0.00%	25.00%	25.00%	75.00%	50.00%

d

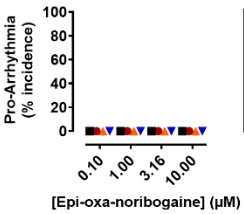

PRO-ARRHYTHMIA	Vehicle	0.1 μM	1 μM	3.16 μM	10 μM
Cell 1	*No*	*No*	*No*	*No*	*No*
Cell 2	*No*	*No*	*No*	*No*	*No*
Cell 3	*No*	*No*	*No*	*No*	*No*
Cell 4	*No*	*No*	*No*	*No*	*No*
Cell 5	*No*	*No*	*No*	*No*	*No*
Cell 6	*No*	*No*	*No*	*No*	*No*
Cumulative Percentage	0.00%	0.00%	0.00%	0.00%	0.00%

After-Contraction % Contraction Failure % Alternans % STV %

e

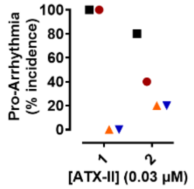

PRO-ARRHYTHMIA	Vehicle	30nM ATX-II
Cell 1	*No*	*Yes*
Cell 2	*No*	*Yes*
Cell 3	*No*	*Yes*
Cell 4	*No*	*Yes*
Cell 5	*No*	*Yes*
Cumulative Percentage	0.00%	100.00%

f

#	Age	Study	Sex	Ethnicity	Body Mass Index (BMI)	Cause of Death (COD)	Ejection Fraction (EF)
1	37	a, b, e1	M	Hispanic / Caucasian	28.5, 29.1 or 31.1	CVA/ICH/Stroke, Blunt Injury or Anoxia	N/A or 80%
2	24	c					
3	43	d, e2					

CVA = Cerebrovascular Accident, **ICH** = Intracranial Hemorrhage
N/A* = Not Available, *Organ procurement organization could not transplant the heart and consequently no echocardiography was performed.

Fig. S8 | Pro-arrhythmia risk of noribogaine and oxa-iboga analogs examined in human primary cardiomyocytes. a Noribogaine was compared to **b** oxa-noribogaine using cardiomyocytes isolated from the same donor heart. **c** Comparable and concentration-dependent pro-arrhythmia risk was detected for noribogaine across 2 donors. **d** Epi-oxa-noribogaine shows no risk of pro-arrhythmia. **e** Anemone toxin, ATX-II, was used as a positive control. **f** Donor information.

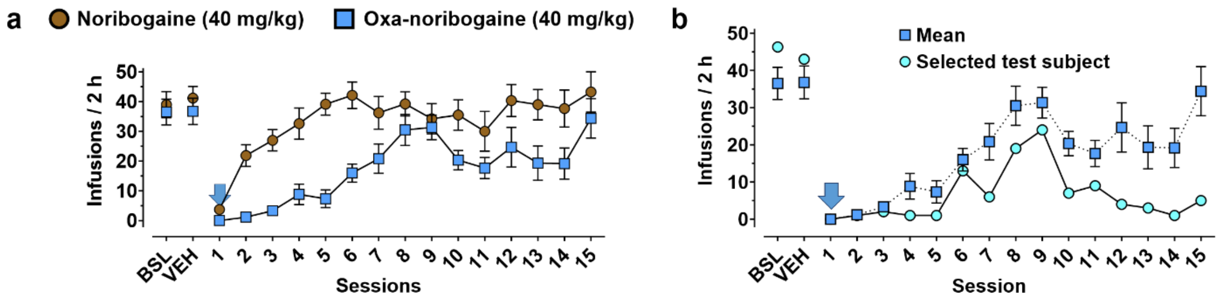

Fig. S9 | Morphine self-administration studies in rats. a Complete time profile of a morphine SA study (Fig 5b) for noribogaine (40 mg/kg, n = 8) and oxa-noribogaine (40 mg/kg, n = 6) treatments. **b** Morphine intake for one test subject that showed a profound and lasting morphine intake suppression (>14 days) after a single dose of oxa-noribogaine (40 mg/kg, i.p.). The mean response of the entire cohort is shown for comparison. Data are presented as mean ± SEM, specifics of statistical tests are reported in source data file, *P < 0.05, **P < 0.01, ***P < 0.001.

Table S1. In vitro data collected for iboga alkaloids and analogs.

**Binding affinities (Radioligand Displacement) [K_i, (95% CI) in nM] for oxa-iboga alkaloids at mouse opioid receptors (n = 3).**		
Receptor	Oxa-noribogaine	Epi-oxa-noribogaine
mMOR	86 (65; 115)	136 (111; 166)
mKOR	36 (29; 44)	6.9 (5.7; 8.3)
mDOR	168 (127; 223)	94 (85; 104)
For radioligand experiments fitted curves were constrained to top = 100 and bottom = 0.		

**Agonist activity (GTPγS assay) [EC_{50}, (95% CI) in nM], {%E_{max}} of iboga alkaloids at mouse opioid receptors (n ≥ 3).**		
Receptor	Oxa-noribogaine	Epi-oxa-noribogaine
mMOR	559 (331, 920) {101}	211 (139, 322) {111}
mKOR	49 (37, 64) {92}	9.6 (7.2, 12.8) {89}
mDOR	329 (91, 117) {100}	99 (75, 130) {108}
Efficacy data were obtained using agonist induced stimulation of [^{35}S]GTPγS binding assay. Efficacy is represented as EC_{50} (nM) and percent maximal stimulation (E_{max}) relative to standard agonist DAMGO (MOR), DPDPE (DOR), or U50,488 (KOR) at 1000 nM.		

**Agonist activity (G protein BRET assay) [EC_{50}, (95% CI) in nM], {%E_{max}} of iboga alkaloids at human and rat kappa opioid receptor (n = 3)**				
Receptor	(±)-U50,488	Noribogaine	Oxa-noribogaine	Epi-oxa-noribogaine
hKOR	13 (8, 20) {100}	7,745 (284; 21,050) {46}	68 (42; 109) {108}	16 (10; 26) {96}
rKOR	18 (11; 32) {100}	6,239 (2,640; 33,510) {52}	43 (17; 126) {82}	12 (7; 23) {81}

**Agonist activity (G protein BRET assay) [EC_{50}, (95% CI) in nM], {%E_{max}} of iboga alkaloids at human and mouse mu opioid receptor (n = 3)**				
Receptor	DAMGO	Noribogaine	Oxa-noribogaine	Epi-oxa-noribogaine
hMOR	3 (2, 4) {100}	11,134 (6,734; 19,260) {57}	88 (69; 113) {108}	90 (54; 147) {69}
mMOR	8 (7; 10) {100}	9,110 (1,941; 53,530) {19}	328 (201; 531) {81}	217 (105; 443) {28}

**Agonist activity (Nb33 BRET assay) [EC_{50}, (95% CI) in nM], {%E_{max}} of iboga alkaloids at human and rat kappa opioid receptor (n = 3)**				
Receptor	(±)-U50,488	Noribogaine	Oxa-noribogaine	Epi-oxa-noribogaine
hKOR	176 (113, 284) {100}	n.d. {<3% at 100 μM}	304 (171; 567) {46}	58 (18; 336) {50}
rKOR	202 (159; 257) {100}	n.d. {<4% at 100 μM}	484 (304; 801) {52}	62 (35; 112) {34}

**Agonist activity (Nb33 BRET assay) [EC_{50}, (95% CI) in nM], {%E_{max}} of iboga alkaloids at human and mouse mu opioid receptor (n = 3)**			
Receptor	DAMGO	Oxa-noribogaine	Epi-oxa-noribogaine
hMOR	233 (170, 322) {100}	13,770 (4,743; 43,640) {31}	n.d.
mMOR	357 (232; 547) {100}	4,281 (2,286; 7,614) {44}	n.d.

β-Arrestin recruitment (BRET assay) [EC_{50}, (95% CI) in nM], {$\%E_{max}$} of iboga alkaloids at human and rat kappa opioid receptor (n = 3), with and without addition of G-protein coupled receptor kinase 3 (GRK3)			
Receptor	(±)-U50,488	Oxa-noribogaine	Epi-oxa-noribogaine
hKOR + GRK3	86 (52, 141) {100}	405 (216; 748) {65}	49 (31; 108) {50}
rKOR + GRK3	129 (64; 276) {100}	610 (314; 997) {69}	128 (60; 194) {50}
hKOR	5,802 (1,515; 3,260,000) {100}	n.d. {13% at 100 µM}	n.d. {11% at 100 µM}
rKOR	1,566 (841; 3,784) {100}	n.d. {11% at 100 µM}	n.d. {<0% at 100 µM}

Agonist activity (G protein BRET assay) [EC_{50} in nM], {$\%E_{max}$} of oxa-ibogamine analogs demonstrating the importance of the phenolic -OH (position 10) on KOR activity (n = 4). Fitted curves were constrained to top = 100% and bottom = 0%.			
Assay (Species)	Receptor	Oxa-ibogamine	Epi-oxa-ibogamine
G protein BRET (human)	hKOR	>10,000 {56% at 100 µM}	>10,000 {46% at 100 µM}

Binding affinities for hNOP, rNMDAR and inhibition of hnAChR (α3β4) ion channels [IC_{50}, (95% CI) in nM] by iboga alkaloids (n = 1). Fitted curves were constrained to top = 100% and bottom = 0%.				
Assay	Receptor	Noribogaine	Oxa-noribogaine	Epi-oxa-noribogaine
Radioligand Displacement	hNOP	n.d. {<0% at 10 µM}	n.d. {<0% at 10 µM}	n.d. {5% at 10 µM}
	rNMDAR	42,220 (35,820; 49,770)	24,060 (17,500; 33,090)	66,800 (63,950; 69,790)
	hERG	/	3,003 (2,028; 4,461)	/
Electrophysiology	hnAChR (α3β4)	4,960 (3,343; 7,360)	2,891 (2,622; 3,188)	1,495 (1,041; 2,147)

Inhibitory activity [IC_{50} (95% CI) in nM] {$\%E_{max}$} of iboga alkaloids at human serotonin transporter (hSERT, n = 3).				
Assay	Imipramine	Noribogaine	Oxa-noribogaine	Epi-oxa-noribogaine
Fluorescence Uptake Inhibition	8.9 (7.0; 11) {100}	286 (216; 372) {104}	711 (560; 908) {106}	1,707 (1,282; 2,394) {120}

Table S2. Summary of behavioral (in vivo) and pharmacokinetic (in vivo, ex vivo detection) data for oxa-iboga analogs.

**Pharmacokinetics data determined for noribogaine in male C57BL/6 mice following a single subcutaneous administration (Dose: 10 mg/kg, n = 3).**							
Matrix	T_{max} (h)	C_{max} (ng/mL)[a]	AUC_{last} (h×ng/mL)	AUC_{inf} (h×ng/mL)	$T_{1/2}z$ (h)	CLf (mL/min/kg)	VZ/F (L/kg)
Plasma	0.50	1979.32	5221.91	5229.14	2.76	31.87	7.62
Brain	0.50	5093.45	11672.46	12112.71	1.71	13.76	2.04
[a] Brain conc. expressed as ng/g, density of brain tissue was considered as 1 which is equivalent to plasma density.							

**Pharmacokinetics data determined for oxa-noribogaine in male C57BL/6 mice following a single subcutaneous administration (Dose: 10 mg/kg, n = 3).**								
Matrix	T_{max} (h)	C_{max} (ng/mL)[a]	AUC_{last} (h×ng/mL)	$T_{1/2}z$ (h)	CLf (mL/min/kg)	Vd/F (L/kg)	Brain-Kp(C_{max})	Brain-Kp(AUC_{last})
Plasma	0.25	581.24	1413.06	6.10	112.73	59.53	N/A	N/A
Brain	0.50	4492.48	7227.68	4.76	N/A	N/A	7.72	5.11
[a] Brain conc. expressed as ng/g, density of brain tissue was considered as 1 which is equivalent to plasma density.								

**Pharmacokinetics data determined for oxa-noribogaine in male Wistar rats following a single intraperitoneal administration (Dose: 40 mg/kg, n = 3).**								
Matrix	T_{max} (h)	C_{max} (ng/mL)[a]	AUC_{last} (h×µg/mL)	$T_{1/2}z$ (h)	CLf (mL/min/kg)	Vd/F (L/kg)	Brain-Kp(C_{max})	Brain-Kp(AUC_{last})
Plasma	0.08	1973.00	3520	1.26	189.29	20.62	N/A	N/A
Brain	0.08	18919.68	55220	3.59	N/A	N/A	9.60	15.69
[a] Brain conc. expressed as ng/g, density of brain tissue was considered as 1 which is equivalent to plasma density.								

**Rat plasma protein binding of Oxa- and Epi-oxa-noribogaine (n = 3).**				
Compound	% Bound ± SD	% Unbound	% Recovery	% Stability (4 h)
Oxa-noribogaine	93.4 ± 0.4	6.6	94	104
Epi-oxa-noribogaine	86.4 ± 1.9	13.6	88	111

**Human plasma protein binding of noribogaine, Oxa- and Epi-oxa-noribogaine (n = 3).**				
Compound	% Bound ± SD	% Unbound ± SD	% Recovery	% Stability (4 h)
Noribogaine	59.9 ± 3.7	40.1	75	105
Oxa-noribogaine	95.8 ± 0.9	4.2	97	116
Epi-oxa-noribogaine	89.4 ± 0.5	10.6	98	108

Rat brain tissue binding of Oxa- and Epi-oxa-noribogaine (n = 3).				
Compound	% Bound ± SD	% Unbound	% Recovery	% Stability (4 h)
Oxa-noribogaine	98.9 ± 0.1	1.1	120	80
Epi-oxa-noribogaine	98.6 ± 0.1	1.4	106	97

Tail-flick test characterization of antinociceptive properties of U50,488, oxa- and epi-oxa-noribogaine [Base Latency, (95% CI) in s; ED_{50}, (95% CI) in mg/kg], in male WT C57BL/6 mice.			
Compound	(±)-U-50,488 (n = 5)*	Oxa-noribogaine (n = 20)	Epi-oxa-noribogaine (n = 9)
Base Latency (s)	3.7 (3.2; 4.1)	2.6 (2.5; 3.9)	2.0 (1.2; 2.7)
ED_{50} (mg/kg)	2.2 (1.6; 3.1)	3.0 (2.6; 3.4)	1.9 (1.4; 2.5)
*5 naive animals were used for each dose, baseline was determined for each test subject			

Tail-flick test characterization of antinociceptive properties of U50,488, oxa- and epi-oxa-noribogaine [Base Latency, (95% CI) in s; ED_{50}, (95% CI) in mg/kg], in female WT C57BL/6 mice.			
Compound	(±)-U-50,488 (n = 5)*	Oxa-noribogaine (n = 10)	Epi-oxa-noribogaine (n = 10)
Base Latency (s)	2.9 (2.6; 3.2)	2.3 (1.8; 2.8)	2.3 (1.7; 2.9)
ED_{50} (mg/kg)	6.3 (4.4; 9.0)	4.9 (3.9; 6.3)	9.7 (6.9; 13.7)
*5 naive animals were used for each dose, baseline was determined for each test subject			

Antinociceptive properties of oxa-noribogaine in wild type (WT), mu (MOR-KO) and kappa (KOR) knockout (KO) mouse models [baseline, (95% CI) in s; ED_{50}, (95% CI) in mg/kg].						
Sex	**Male**			**Female**		
Model	**WT (n = 10)**	**KOR-KO (n = 8)**	**MOR-KO (n = 9)**	**WT (n = 10)**	**KOR-KO (n = 4)**	**MOR-KO (n = 5)**
Base Latency (s)	2.5 (2.3, 2.7)	2.7 (2.4, 3.0)	3.0 (2.7, 3.2)	3.2 (2.8, 3.5)	3.5 (2.6, 4.4)	3.5 (3.3, 3.8)
ED_{50} (mg/kg)	2.3 (1.9; 2.7)	17.7 (14.4; 21.9)	4.3 (3.2; 5.9)	2.0 (1.5; 2.5)	12.4 (7.4; 20.9)	7.1 (4.0; 12.6)

Antinociceptive properties of epi-oxa-noribogaine in wild type (WT), mu (MOR-KO) and kappa (KOR) knockout (KO) mouse models [baseline, (95% CI) in s; ED_{50}, (95% CI) in mg/kg].			
Sex	**Male**		
Model	**WT (n = 5)**	**KOR-KO (n = 5)**	**MOR-KO (n = 7)**
Base Latency (s)	2.3 (1.9, 2.6)	2.2 (2.0, 2.4)	3.4 (2.7, 4.0)
ED_{50} (mg/kg)	1.3 (0.9; 1.7)	n.d. ≫100	2.0 (1.5; 2.6)

Synthetic procedures and compound characterization

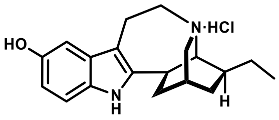

Noribogaine hydrochloride

Noribogaine hydrochloride

The compound was synthetized as previously described starting from Voacangine[1] isolated from root bark of *Voacanga africana* according to published isolation procedure.[2] To avoid complications arising from legal restrictions on ibogaine synthesis (controlled substance schedule I in the US) a synthetic route utilizing 10-hydroxy-coronaridine as intermediate was used (Fig. S2).

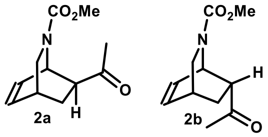

Synthesis of *exolendo*-isoquinuclidine ketone intermediates 2a and 2b

The isoquinuclidine core was synthesized according to the reported literature procedures.[3,4] Briefly, methyl pyridine-1(2*H*)-carboxylate **1** was prepared by treating a mixture of pyridine and sodium borohydride in methanol with methyl chloroformate at -70°C. The crude diene (unstable, slowly decomposing during storage, should be used for next step as soon as possible) was filtered through silica (eluted with 10% diethyl ether in hexanes), concentrated and then heated with methyl vinyl ketone at 50 °C for 5 days to form a mixture of *exo* **2a** and *endo* **2b** compounds in a 26:74 ratio. The mixture was further epimerized using sodium methoxide in methanol to obtain a new ratio of *exo:endo* 62:38.

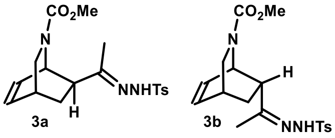

Synthesis of *exo*/*endo*-isoquinuclidine tosylhydrazones 3a and 3b

A mixture of exo/endo-intermediates **2a/2b** (62:38 *exo*:*endo* ratio, 34.93 g, 167 mmol) and *p*-toluenesulfonyl hydrazide (31.10 g, 167 mmol) in THF (136 mL) was heated at 50 °C for 15 h, at which time a white precipitate had formed. The reaction mixture was cooled to room temperature and the white precipitate was collected by filtration, the solid was washed 3× on the filter with ice-cold MeOH, to provide pure *endo*-tosylhydrazone **3b** as a fine white powder (19.55 g, 31%). The filtrate and washings were combined and concentrated to a tan solid, which was recrystallized from MeOH to obtain the pure *exo*-tosylhydrazone **3a** as white plates (33.14 g, 53%).

***exo*-Methyl 7-(1-(2-tosylhydrazono)ethyl)-2-azabicyclo[2.2.2]oct-5-ene-2-carboxylate (3a)**

Mp 162-166 °C (inaccurate due to trapped MeOH); **^{1}H NMR (400 MHz, $CDCl_3$)** (spectrum complicated by conformers) δ 7.85 and 7.79 (d, *J* = 8.3 Hz, 2H), 7.35 – 7.28 (m, 3H), 6.46 – 6.37 (m, 2H), 4.74 – 4.68 and 4.61 – 4.57 (m, 1H), 3.52 and 3.34 (s, 3H), 3.02 and 2.94 (dd, *J* = 9.8, 2.2 Hz, 1H), 2.87 and 2.78 (dt, *J* = 9.8, 2.6 Hz, 1H), 2.73 – 2.67 (m, 1H), 2.48 – 2.37 (m, 1H), 2.44 (s, 3H), 2.30 and 2.09 (ddd, *J* = 13.1, 4.4, 2.4 Hz, 1H), 1.89 and 1.80 (s, 3H), 1.42 – 1.26 (m, 1H); **LR-MS** (m/z): calcd. for $C_{18}H_{24}N_3O_4S^+$ $[M+H]^+$ 378.15, found 377.80.

***endo*-Methyl 7-(1-(2-tosylhydrazono)ethyl)-2-azabicyclo[2.2.2]oct-5-ene-2-carboxylate (3b)**

Mp 181-184 °C; **^{1}H NMR (400 MHz, $CDCl_3$)** (spectrum complicated by conformers) δ 7.81 (t, *J* = 7.4 Hz, 2H), 7.32 (d, *J* = 8.0 Hz, 2H), 7.12 (s, 1H), 6.19 (q, *J* = 7.5 Hz, 1H), 6.02 (dd, *J* = 12.0, 5.6 Hz, 1H), 4.87 and 4.77 (d, *J* = 4.3 Hz, 1H), 3.69 and 3.66 (s, 3H), 3.23 (d, *J* = 10.1 Hz, 1H), 3.01 – 2.85 (m, 2H), 2.74 (br s, 1H), 2.45 and 2.44 (s, 3H), 1.87 – 1.66 (m, 1H), 1.71 and 1.69 (s, 3H), 1.59 – 1.47 (m, 1H); **LR-MS** (m/z): calcd. for $C_{18}H_{24}N_3O_4S^+$ $[M+H]^+$ 378.15, found 377.80.

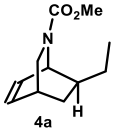

***exo*-Methyl 7-ethyl-2-azabicyclo[2.2.2]oct-5-ene-2-carboxylate (4a)**

exo-Tosylhydrazone **3a** (33.02 g, 87.5 mmol), sodium cyanoborohydride (21.99 g, 350 mmol), and *p*-toluenesulfonic acid monohydrate (1.40 g, 7.36 mmol) were combined in THF (250 mL) and refluxed for 21 h. At this time, additional *p*-TsOH·H_2O (0.35 g, 1.84 mmol) was added, and reflux was continued for an additional 4 h. The reaction mixture was then diluted with water (250 mL) and extracted with cyclohexane (3 × 100 mL). The combined organics were washed with water (250 mL), saturated aqueous $NaHCO_3$ (250 mL), and water again (50 mL), dried over Na_2SO_4, and concentrated to provide a cloudy, pale-yellow oil. The oil was passed through a short silica column in 7:3 hexanes:EtOAc and the eluate was concentrated to yield pure product as a pale-yellow oil (10.12 g, 59%). The spectral characterization was in agreement with the previously reported literature data.[5]

^{1}H NMR (400 MHz, $CDCl_3$) δ 6.47 and 6.42 (br dd, *J* = 7 Hz, 1H), 6.34 and 6.30 (br dd, *J* = 8 Hz, 1H), 4.59 and 4.44 (d, *J* = 6 Hz, 1H), 3.67 and 3.66 (s, 3H), 3.20 (td, *J* = 10, 2 Hz, 1H), 2.98 and 2.94 (dt, *J* = 10, 3 Hz, 1H), 2.65 (m, 1H), 1.64 and 1.61 (dt, *J* = 10, 3 Hz, 1H), 1.38 (m, 3H), 1.00 (m, 1H), 0.95 and 0.92 (t, *J* = 7 Hz, 3H); **^{13}C NMR (101 MHz, $CDCl_3$)** δ 156.7 and 156.3, 133.8 and 133.7, 133.3 and 133.1, 52.2 and 52.1, 49.1 and 48.8, 48.4 and 48.1, 40.7, 30.8 and 30.6, 29.9, 27.5 and 27.4, 12.1 and 12.0; **LR-MS** (m/z): calcd. for $C_{11}H_{18}NO_2^+$ $[M+H]^+$ 196.13, found 195.9.

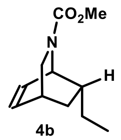

4b

endo-Methyl 7-ethyl-2-azabicyclo[2.2.2]oct-5-ene-2-carboxylate (4b)

To a suspension of endo-tosylhydrazone **3b** (3.50 g, 9.27 mmol) in MeOH (41 mL) was added a solution of sodium cyanoborohydride (0.83 g, 13.26 mmol) and $ZnCl_2$ (0.90 mg, 6.63 mmol) in MeOH (28 mL) and the resulting mixture was refluxed for 3 h. The reaction was then quenched with 1% aqueous NaOH (200 mL) and extracted with cyclohexane (3 × 50 mL). The combined organics were washed with water (50 mL) and brine (50 mL), dried over Na_2SO_4, and concentrated to provide a clear, colorless oil. The crude material was filtered through a short silica column with 1:1 hexanes:EtOAc and the eluate was concentrated to yield pure product as a colorless oil (1.11 g, 61%).

^{1}H NMR (400 MHz, $CDCl_3$) δ 6.31 (m, 2H), 4.66 and 4.48 (s, 1H), 3.69 and 3.66 (s, 3H), 3.21 (m, 1H), 2.94 (m, 1H), 2.69 (m, 1H), 1.96 (m, 1H), 1.81 (m, 1H), 1.19 (m, 1H), 1.01 - 0.84 (m, 5H); **^{13}C NMR (101 MHz, $CDCl_3$)** δ 155.6 and 155.2, 134.4 and 134.0, 130.6 and 130.0, 52.0 and 51.9, 49.3 and 48.9, 46.9 and 46.5, 40.7 and 40.5, 31.0 and 30.8, 30.0, 28.3 and 28.2, 11.2; **LR-MS** (m/z): calcd. for $C_{11}H_{18}NO_2^+$ $[M+H]^+$ 196.13, found 195.90.

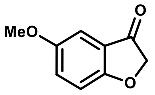

5

5-Methoxybenzofuran-3(2H)-one (5)

Benzofuranone **5** was prepared from 4-methoxyphenol according to literature procedure[6] and obtained as orange-tan crystals (yield 30 – 40%).

^{1}H NMR (500 MHz, $CDCl_3$) δ 7.24 (dd, *J* = 9.0, 2.8 Hz, 1H), 7.06 (d, *J* = 5.8 Hz, 1H), 7.05 (s, 1H), 4.64 (s, 2H), 3.80 (s, 3H); **^{13}C NMR (126 MHz, $CDCl_3$)** δ 200.3, 169.5, 155.2, 128.1, 121.2, 114.7, 104.0, 75.6, 56.1; **LR-MS** (m/z): calcd. for $C_9H_9O_3^+$ $[M+H]^+$ 165.06, found 164.99.

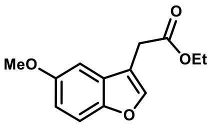

6a

Ethyl 2-(5-methoxybenzofuran-3-yl)acetate (6a)

A solution of benzofuranone **5** (4.92 g, 30.00 mmol) and (carbethoxymethylene)triphenylphosphorane (11.50 g, 33.00 mmol) in toluene (100 mL) was refluxed for 110 h and then concentrated under reduced pressure. The resulting material was triturated with 9:1 hexanes:EtOAc (210 mL) and filtered, and the remaining solids were washed with additional portions of 9:1 hexanes:EtOAc (3 × 90 mL). The combined filtrates were concentrated to give the crude product, which was purified by column chromatography (1:1 hexanes:CH_2Cl_2, 5 column volumes → CH_2Cl_2, 3 column volumes → 1:1 CH_2Cl_2:Et_2O, 1 column volume). Collected fractions were concentrated to provide product **6** as a thin, yellow-orange oil (5.68 g, 81%). The spectral characterization was in agreement with the previously reported literature data.[7]

^{1}H NMR (400 MHz, $CDCl_3$) δ 7.60 (s, 1H), 7.36 (d, *J* = 9.0 Hz, 1H), 7.02 (d, *J* = 2.6 Hz, 1H), 6.91 (dd, *J* = 8.9, 2.6 Hz, 1H), 4.19 (q, *J* = 7.1 Hz, 2H), 3.85 (s, 3H), 3.66 (d, *J* = 1.0 Hz, 2H), 1.28 (t, *J* = 7.1 Hz, 3H); **^{13}C NMR (101 MHz, $CDCl_3$)** δ 170.8, 156.1, 150.4, 143.8, 128.3, 113.42, 113.39, 112.1, 102.3, 61.2, 56.1, 30.1, 14.4; **LR-MS** (m/z): calcd. for $C_{13}H_{15}O_4^+$ $[M+H]^+$ 235.10, found 235.37.

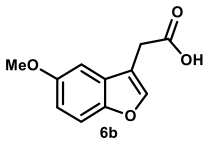

6b

2-(5-methoxybenzofuran-3-yl)acetic acid (6b)

Compound **6b** was prepared using slightly modified published reaction conditions.[8] A mixture of 4-methoxyphenol (24.8 g, 200 mmol) and ethyl 4-chloroacetoacetate (32.64 mL, 240.00 mmol) was cooled in an ice bath and cold (~ 4 °C) 70% aqueous sulfuric acid was added (1 mL of acid per 1 mmol of phenol). Reaction mixture was further stirred for 1-3 days and quenched with ice and ice-cold water (final volume ~500 mL). The resulting beige suspension was further stirred for 1 h and was extracted with CH_2Cl_2 (3 × 250 mL). Combined dark brown extracts were dried over Na_2SO_4 and filtered through a plug of silica, washing the plug with more CH_2Cl_2, until all product eluted (solvent was recycled by evaporation and reused for further elution). Collected fractions were concentrated to yield the coumarin intermediate as a bright yellow solid (35.6 g). The crude intermediate was then suspended in 1M NaOH (500 mL) and heated to reflux for 2 h. After cooling to room temperature pH of reaction mixture was adjusted to ~6 using concentrated (36-38%) aq. hydrochloric acid (~20 mL) and was back adjusted to 7 using addition of solid $NaHCO_3$. Neutral aqueous solution was washed with diethyl ether (2 × 150 mL, removal of unreacted phenol). Aqueous mixture was further acidified to pH ~2 using concentrated (36-38%) aq. hydrochloric acid and the formed suspension was extracted with CH_2Cl_2 (4 × 250 mL, product dissolves slowly initially). Combined extracts were dried over Na_2SO_4, filtered and concentrated to yield the crude acid **6b** (~90-95% pure) as a beige solid (25.00 g, 58%). The crude material was used as is for the next step. The spectral characterization was in agreement with the previously reported literature data.[8]

^{1}H NMR (400 MHz, $CDCl_3$) δ 10.73 (s, 1H), 7.61 (d, *J* = 1.1 Hz, 1H), 7.37 (d, *J* = 8.9 Hz, 1H), 7.00 (d, *J* = 2.6 Hz, 1H), 6.92 (dd, *J* = 8.9, 2.6 Hz, 1H), 3.85 (s, 3H), 3.73 (d, *J* = 1.1 Hz, 2H). **^{1}H NMR (500 MHz, DMSO)** δ 12.42 (s, 1H), 7.84 (d, *J* = 1.2 Hz, 1H), 7.45 (d, *J* = 8.9 Hz, 1H), 7.11 (d, *J* = 2.6 Hz, 1H), 6.90 (dd, *J* = 8.9, 2.6 Hz, 1H), 3.78 (s, 3H), 3.67 (d, *J* = 1.1 Hz, 2H); **LR-MS** (m/z): calcd. for $C_{11}H_{10}O_4^-$ $[M-H]^-$ 205.1, found 205.4.

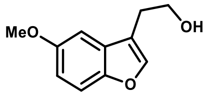

7

2-(5-Methoxybenzofuran-3-yl)ethanol (7)

From **6a**: To a suspension of $LiAlH_4$ (2.36 g, 62.09 mmol) in THF (60 mL) at room temperature was carefully added a solution of the ester **6a** (5.60 g, 23.88 mmol) in THF (20 mL), and the mixture was refluxed for 30 min. After cooling to room temperature, the reaction was quenched by the successive addition of H_2O (2.4 mL), 15% aqueous NaOH (2.4 mL), and H_2O again (7.2 mL). The resulting mixture was stirred vigorously until the aluminum salts were white and loose and then filtered, washing the filter cake with Et_2O (3 × 60). The combined filtrate and washings were concentrated to yield the product **7** directly as a yellow oil (4.50 g, 98%). The spectral characterization was in agreement with the previously reported literature data.[9]

From **6b**: Suspension of $LiAlH_4$ (2.85 g, 75.0 mmol) in THF (30.0 mL) was cooled using water/ice-bath and the solution of crude (~90-95% pure) acid **6b** (6.51 g, ~30.00 mmol) in THF (30.0 mL) was added in small portions via canula. Reaction mixture was allowed to warm to room temperature and stirred until no more starting material was detected by TLC (<1 h). The reaction mixture was again cooled using water/ice-bath before adding diethyl ether (not anhydrous, 60 mL) and carefully quenching it by the successive addition of H_2O (2.9 mL), 15% aqueous NaOH (2.9 mL), and H_2O again (8.7 mL). The resulting mixture was stirred vigorously until the aluminum salts were pale and loose, suspension was dried by addition of $MgSO_4$ and filtered, washing the filter cake with diethyl ether until no more product eluted. The combined filtrate and washings were concentrated to yield the product **7** directly as a yellow oil (5.23 g, 90%).

^{1}H NMR (500 MHz, $CDCl_3$) δ 7.48 (s, 1H), 7.36 (d, *J* = 8.9 Hz, 1H), 7.00 (d, *J* = 2.6 Hz, 1H), 6.90 (dd, *J* = 8.9, 2.6 Hz, 1H), 3.91 (t, *J* = 6.4 Hz, 2H), 3.85 (s, 3H), 2.91 (t, *J* = 6.4 Hz, 2H), 1.74 (s, 1H); **^{13}C NMR (126 MHz, $CDCl_3$)** δ 156.0, 150.5, 143.2, 128.7, 117.0, 113.2, 112.1, 102.3, 61.9, 56.1, 27.2; **LR-MS** (m/z): calcd. for $C_{11}H_{13}O_3^+$ [M+H]$^+$ 193.09, found 193.35.

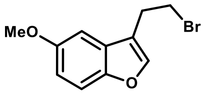

8

3-(2-Bromoethyl)-5-methoxybenzofuran (8)

To a solution of the alcohol **7** (2.18 g, 11.34 mmol) and carbon tetrabromide (5.64 g, 17.01 mmol) in CH_2Cl_2 (anhydrous, 23 mL) at room temperature was carefully added triphenylphosphine (4.46 g, 17.01 mmol) and the resulting dark orange-brown mixture was left to stir for 20 min. The reaction mixture was filtered through a silica plug to remove baseline impurities, washing the plug with additional CH_2Cl_2 until TLC indicated that all product was eluted. The filtrate was then concentrated and purified by column chromatography (hexanes, 2 column volumes → 20:1 hexanes:Et_2O, 2 column volumes → 10:1 hexanes:Et_2O, 2 column volumes) to provide a pale-yellow oil that slowly crystallized to a white solid (2.81 g, 97%). The spectral characterization was in agreement with the previously reported literature data.[10]

^{1}H NMR (500 MHz, $CDCl_3$) δ 7.51 (s, 1H), 7.37 (d, *J* = 8.9 Hz, 1H), 6.97 (d, *J* = 2.5 Hz, 1H), 6.91 (dd, *J* = 8.9, 2.6 Hz, 1H), 3.86 (s, 3H), 3.64 (t, *J* = 7.4 Hz, 2H), 3.23 (td, *J* = 7.4, 0.7 Hz, 2H); **^{13}C NMR (126 MHz, $CDCl_3$)** δ 156.1, 150.4, 143.1, 128.1, 117.8, 113.2, 112.3, 101.9, 56.2, 31.3, 27.7; **LR-MS** (m/z): calcd. for $C_{11}H_{12}BrO_2^+$ [M+H]$^+$ 255.00 and 257.00, found 255.29 and 257.29.

General Procedure for Preparation of N-benzofuranylethylisoquinuclidines

To a solution of a carbamate protected isoquinuclidine **4** (1 equivalent) in CH_2Cl_2 (anhydrous, 0.125 M, based on 2) at 0 °C was added iodotrimethylsilane (4 equivalents), and the resulting mixture was stirred for 10 min at 0 °C and then at room temperature until TLC indicated that no **4** remained (typically ~1 h). The reaction mixture was then quenched with MeOH (3.0 mL per mmol of **4**) and concentrated to yield the deprotected isoquinuclidine hydroiodide salt in quantitative yield. To this material was added 3-(2-bromoethyl)-5-methoxybenzofuran **8** (1 equivalent) and $NaHCO_3$ (4 equivalents), followed by anhydrous CH_3CN (0.208 M, based on **4**), and the resulting mixture was refluxed until TLC indicated the disappearance of the bromide (typically >24 h). The reaction was then diluted with water, made strongly basic with aqueous NaOH, and extracted with CH_2Cl_2 (3×). The combined organics were washed with water, dried over Na_2SO_4, and concentrated to provide the crude product, which was purified by column chromatography with an appropriate solvent mixture (as described below for each compound).

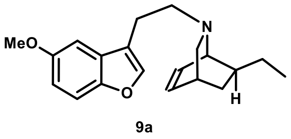

9a

***exo*-7-Ethyl-2-(2-(5-methoxybenzofuran-3-yl)ethyl)-2-azabicyclo[2.2.2]oct-5-ene (9a)**

The product **9a** was prepared according to the general procedure and purified by column chromatography (20:1 hexanes:Et_2O, 3 column volumes → 20:1 hexanes:Et_2O + 2% Et_3N, 5 column volumes) to provide a pale-yellow oil (288 mg, 74%).

*Note (multigram scale preparation) reaction was scaled up to 30 mmol of starting materials **8** and **4a** to yield **9a** (7.02g, 75%).*

^{1}H NMR (400 MHz, $CDCl_3$) δ 7.46 (s, 1H), 7.33 (d, *J* = 8.9 Hz, 1H), 6.98 (d, *J* = 2.6 Hz, 1H), 6.87 (dd, *J* = 8.9, 2.6 Hz, 1H), 6.39 – 6.27 (m, 2H), 3.86 (s, 3H), 3.23 (dt, *J* = 5.3, 1.9 Hz, 1H), 3.09 (dd, *J* = 9.1, 2.3 Hz, 1H), 2.84 – 2.63 (m, 3H), 2.57 – 2.48 (m, 1H), 2.48 – 2.40 (m, 1H), 1.94 (dt, *J* = 9.1, 2.6 Hz, 1H), 1.63 –

1.42 (m, 3H), 1.35 – 1.24 (m, 1H), 0.95 – 0.90 (m, 1H), 0.88 (t, *J* = 7.4 Hz, 3H); **^{13}C NMR (101 MHz, $CDCl_3$)** δ 155.8, 150.3, 142.6, 133.1, 132.8, 129.2, 119.2, 112.6, 111.8, 102.5, 57.8, 56.3, 56.3, 56.2, 41.3, 31.8, 29.9, 27.4, 23.1, 12.6; **HR-MS** (m/z): calcd. for $C_{20}H_{26}NO_2^+$ $[M+H]^+$ 312.1964, found 312.1948.

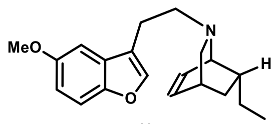

9b

***endo*-7-Ethyl-2-(2-(5-methoxybenzofuran-3-yl)ethyl)-2-azabicyclo[2.2.2]oct-5-ene (9b)**

The product **9b** was prepared according to the general procedure and purified by column chromatography (gradient of 5, 10 to 15% of EtOAc in hexanes + 2% Et_3N) to provide a pale-yellow oil (582 mg, 75%).

^{1}H NMR (500 MHz, $CDCl_3$) δ 7.42 (s, 1H), 7.33 (d, *J* = 8.9 Hz, 1H), 7.00 (d, *J* = 2.6 Hz, 1H), 6.88 (dd, *J* = 8.8, 2.6 Hz, 1H), 6.42 – 6.35 (m, 1H), 6.17 – 6.10 (m, 1H), 3.85 (s, 3H), 3.41 – 3.34 (m, 1H), 3.02 (dd, *J* = 9.7, 2.0 Hz, 1H), 2.88 – 2.70 (m, 3H), 2.57 – 2.48 (m, 2H), 2.07 (dt, *J* = 9.6, 2.7 Hz, 1H), 2.05 – 1.98 (m, 1H), 1.78 (ddd, *J* = 12.2, 9.2, 2.9 Hz, 1H), 1.22 – 1.13 (m, 1H), 1.05 – 0.95 (m, 1H), 0.85 (t, *J* = 7.3 Hz, 3H), 0.79 (ddt, *J* = 12.2, 5.1, 2.8 Hz, 1H); **^{13}C NMR (126 MHz, $CDCl_3$)** δ 155.8, 150.3, 142.4, 133.7, 130.2, 129.0, 118.9, 112.7, 111.9, 102.4, 57.8, 57.4, 56.1, 54.5, 40.9, 31.6, 30.7, 28.8, 23.2, 11.8; **HR-MS** (m/z): calcd. for $C_{20}H_{26}NO_2^+$ $[M+H]^+$ 312.1964, found 312.1955.

General Procedure for Preparation of Oxa-ibogaine Analogs by Ni(0)-catalyzed Cyclization

In a glovebox, a vial was charged with $Ni(COD)_2$ (0.20 equivalents) and 1,3- bis(2,4,6-trimethylphenyl)-1,3-dihydro-*2H*-imidazol-2-ylidene (IMes, 0.24 equivalents) followed by heptane (0.100 M, based on $Ni(COD)_2$), and the resulting black solution was stirred at room temperature for 15 min. To this mixture was then added a solution of the substrate **9** (1 equivalent) in heptane (0.333 M, based on **9**), and the reaction vessel was sealed with a Teflon lined solid cap, removed from the glovebox, and heated at 130 °C for 3 h. After cooling to room temperature, the reaction mixture was purified as described below for each compound.

Note, especially for larger synthetic scale using nonane/decane as the solvent is preferred to avoid over pressurization of reaction vessel.

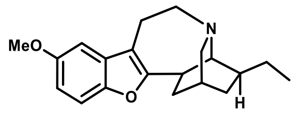

10a

Oxa-ibogaine (10a)

Prepared according to the general procedure. The crude reaction mixture was purified directly by column chromatography (30:1 hexanes:EtOAc + 1% Et_3N) to yield the crude product as a pale-yellow oil. This material was further purified by preparative TLC (30:1 hexanes:EtOAc + 1% Et_3N) to provide the pure product **10a** as a pale-brown oil (118 mg, 76%).

^{1}H NMR (500 MHz, $CDCl_3$) δ 7.24 (d, *J* = 8.7 Hz, 1H), 6.86 (d, *J* = 2.6 Hz, 1H), 6.81 (dd, *J* = 8.7, 2.6 Hz, 1H), 3.85 (s, 3H), 3.45 – 3.36 (m, 1H), 3.26 – 3.11 (m, 3H), 3.02 – 2.91 (m, 2H), 2.82 – 2.78 (m, 1H), 2.53 – 2.44 (m, 1H), 2.08 – 2.00 (m, 1H), 1.88 – 1.76 (m, 2H), 1.67 – 1.61 (m, 1H), 1.59 – 1.42 (m, 3H), 1.24 – 1.15 (m, 1H), 0.91 (t, *J* = 7.1 Hz, 3H); **^{13}C NMR (126 MHz, $CDCl_3$)** δ 161.1, 155.8, 148.6, 131.4, 111.8, 111.4, 111.0, 101.9, 57.2, 56.2, 53.3, 49.7, 41.5, 41.2, 33.1, 32.3, 27.5, 26.5, 19.5, 11.9; **HR-MS** (m/z): calcd. for $C_{20}H_{26}NO_2^+$ $[M+H]^+$ 312.1964, found 312.1956.

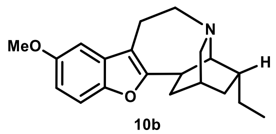

10b

Epi-oxa-ibogaine (10b)

Prepared according to the general procedure. The crude reaction mixture was purified directly by column chromatography (9:1 hexanes:EtOAc + 2% Et_3N, 4 column volumes → 8:2 hexanes:EtOAc + 2% Et_3N, 3 column volumes) to yield the crude product as a yellow-orange oil. This material was further purified by preparative TLC (Et_2O + 1% Et_3N) to provide the pure product **10b** as a nearly colorless oil that slowly crystallized to a white solid (27.0 mg, 28%). **^{1}H NMR (400 MHz, $CDCl_3$)** δ 7.24 (d, *J* = 8.8 Hz, 1H), 6.87 (d, *J* = 2.6 Hz, 1H), 6.80 (dd, *J* = 8.8, 2.6 Hz, 1H), 3.85 (s, 3H), 3.43 (ddd, *J* = 13.7, 4.7, 2.3 Hz, 1H), 3.38 – 3.15 (m, 3H), 3.06 (qt, *J* = 9.5, 2.5 Hz, 2H), 2.86 (t, *J* = 2.3 Hz, 1H), 2.46 (dt, *J* = 16.3, 3.1 Hz, 1H), 2.09 – 1.90 (m, 3H), 1.92 – 1.84 (m, 1H), 1.66 – 1.56 (m, 1H), 1.45 – 1.31 (m, 2H), 1.18 – 1.05 (m, 1H), 0.93 (t, *J* = 7.3 Hz, 3H); **^{13}C NMR (101 MHz, $CDCl_3$)** δ 161.7, 155.9, 148.4, 131.3, 112.3, 111.5, 111.0, 101.8, 56.5, 56.2, 53.5, 49.1, 42.0, 34.5, 34.1, 31.7, 28.5, 26.4, 19.0, 12.3; **HR-MS** (m/z): calcd. for $C_{20}H_{26}NO_2^+$ $[M+H]^+$ 312.1964, found 312.1983.

General Procedure for Preparation of Oxa-noribogaine Analogs by Demethylation

To a solution of the oxa-ibogaine **10** (1 equivalent) in CH_2Cl_2 (0.125 M, based on **10**) at 0 °C was added aluminum chloride (6 equivalents) followed by ethanethiol (18 equivalents), and the resulting mixture was allowed to warm to room temperature and stirred until TLC indicated the complete consumption of starting material (typically <1.5 h). The reaction was then quenched with saturated aqueous $NaHCO_3$ (100 mL per mmol of **10**) and extracted with CH_2Cl_2 (4 × to 6 ×, until no further extraction detected by TLC). The combined organic layers were dried over Na_2SO_4 and concentrated to provide the crude product. This material was purified by column chromatography with an appropriate solvent mixture (as described below for each compound).

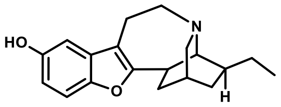

11a
Oxa-noribogaine

***rac*-oxa-noribogaine (Oxa-noriboga, 11a)**

The product **11a** was prepared according to the general procedure and purified by column chromatography (1:1 hexanes:EtOAc) to provide a white, foamy solid (24.3 mg, 82%).

*Note (multigram scale preparation starting from **9a**):* In glovebox $Ni(COD)_2$ (0.94 g, 3.42 mmol) and IMes (1.25 g, 4.10 mmol) were balanced into three 40 mL oven-dried scintillation vials (divided in equal portions), sealed with cap with teflon septa and electrical (vinyl) tape and removed from glove box. Decane (11.4 mL per vial) was added into each vial and the black mixture was vigorously stirred for ~15 min (sonication was used to improve initial dissolution of components). Starting material **9** (5.32 g, 17.08 mmol) was dissolved in decane under Argon and divided equally among the reaction vials washing the original container 2 times with fresh solvent (total volume 51.6 mL decane, solution divided equally per vial). Dark mixture was heated to 130°C (135-140 °C vial heating block) and stirred vigorously. After 3 h, mixture was cooled to room temperature and directly purified by repeated column chromatography (30:1 hexanes:EtOAc + 1% Et_3N). After first column crude material was dissolved in hot hexanes, colored insoluble impurities were filtered off and washed with hexanes. After second column chromatography slightly impure product (5.32 g) was dissolved in CH_2Cl_2 (71.2 mL) and cooled in ice-water bath. Aluminum chloride (6.83 g, 51.24 mmol) followed by ethanethiol (11.8 mL, 153.72 mmol) were added at 0 °C and the resulting mixture was allowed to warm to room temperature and stirred. After 3 h, reaction mixture was poured into a mixture of saturated solution of sodium bicarbonate (150 mL) and solution of potassium sodium tartrate (Rochelle salt, 2 eq. per $AlCl_3$,

28.9 g) in H_2O (150 mL) and the mixture was vigorously stirred and shaken until all aluminum salts dissolved. Aqueous phase was further extracted with CH_2Cl_2 (4 × 100 mL), combined extracts were dried over Na_2SO_4, desiccant was filtered, and solution was concentrated to a pink foamy solid. Crude material was purified by repeated column chromatography (gradient of 20 to 40% EtOAc in hexanes + 2% Et_3N). Slightly impure product was repeatedly dissolved in aqueous ethanol and concentrated to remove all triethylamine trapped as the partial phenolate salt. Material was then dissolved in methanol (~15 mL per 1 g of solid) and acidified by a dropwise addition of concentrated hydrochloric acid (12.1 M, 36-38%), until the solution gave strongly acidic response on pH paper. Solution was concentrated, suspended in acetonitrile, concentrated again and solid thoroughly dried. Crude hydrochloride salt was suspended in acetonitrile (~10 mL per 1 g of solid), suspension was briefly heated to reflux (colored impurities dissolve) and cooled to room temperature. Oxa-noribogaine hydrochloride was collected by filtration, washed with acetonitrile (~5 mL per 1 g of solid) and diethyl ether and air dried. Material was further suspended in saturated sodium bicarbonate solution and repeatedly extracted with mixture of 9:1 CH_2Cl_2:isopropanol, until no further extraction was detected by TLC. Combined extracts were dried over sodium sulfate, filtered and concentrated. To remove traces of solvents used, the material was dissolved in aqueous ethanol and concentrated. The obtained foamy solid material was crushed to release trapped solvent residue and dried overnight at 45 °C under high vacuum. Oxa-noribogaine was obtained as an off-white (pale grey) amorphous solid (3.49 g, 67% over two steps), yield was corrected for 1.9% of ethanol remaining in the material even after extensive drying.
^{1}H NMR (500 MHz, $CDCl_3$) δ 7.19 (d, *J* = 8.6 Hz, 1H), 6.80 (d, *J* = 2.5 Hz, 1H), 6.70 (dd, *J* = 8.6, 2.6 Hz, 1H), 4.74 (br, 1H), 3.45 – 3.33 (m, 1H), 3.21 – 3.09 (m, 3H), 3.00 – 2.91 (m, 2H), 2.81 (d, *J* = 2.2 Hz, 1H), 2.48 – 2.36 (m, 1H), 2.09 – 1.99 (m, 1H), 1.89 – 1.76 (m, 2H), 1.64 (dq, *J* = 13.3, 3.2 Hz, 1H), 1.61 – 1.41 (m, 3H), 1.21 (ddt, *J* = 12.7, 6.5, 2.4 Hz, 1H), 0.91 (t, *J* = 7.1 Hz, 3H); **^{13}C NMR (126 MHz, $CDCl_3$)** δ 161.1, 151.4, 148.6, 131.8, 111.64, 111.55, 110.9, 104.2, 57.3, 53.3, 49.6, 41.6, 41.1, 33.0, 32.2, 27.5, 26.4, 19.4, 12.0; **HR-MS** (m/z): calcd. for $C_{19}H_{24}NO_2^+$ [M+H]$^+$ 298.1807, found 298.1818.

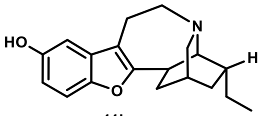

11b
Epi-oxa-noribogaine

***rac*-epi-oxa-noribogaine (Epi-oxa, 11b)**
The product **11b** was prepared according to the general procedure and purified by column chromatography (20:1 CH_2Cl_2:MeOH, 4 column volumes → 20:1 acetone:MeOH, 4 column volumes) to provide a white, foamy solid (13.7 mg, 92%).
^{1}H NMR (500 MHz, $CDCl_3$) δ 7.19 (d, *J* = 8.7 Hz, 1H), 6.79 (d, *J* = 2.4 Hz, 1H), 6.71 (dd, *J* = 8.7, 2.5 Hz, 1H), 5.62 (br s, 1H), 3.40 (ddd, *J* = 14.2, 4.6, 2.4 Hz, 1H), 3.34 – 3.26 (m, 2H), 3.20 – 3.08 (m, 2H), 3.02 (d, *J* = 9.8 Hz, 1H), 2.91 (s, 1H), 2.43 (dt, *J* = 16.6, 2.9 Hz, 1H), 2.08 – 1.95 (m, 3H), 1.90 (s, 1H), 1.65 – 1.58 (m, 1H), 1.44 – 1.33 (m, 2H), 1.15 – 1.09 (m, 1H), 0.92 (t, *J* = 7.3 Hz, 3H); **^{13}C NMR (126 MHz, $CDCl_3$)** δ 161.5, 152.1, 148.2, 131.4, 112.11, 112.06, 111.0, 104.2, 56.5, 53.6, 49.0, 41.2, 34.0, 33.9, 31.4, 28.4, 26.1, 18.8, 12.3.; **HR-MS** (m/z): calcd. for $C_{19}H_{24}NO_2^+$ [M+H]$^+$ 298.1807, found 298.1818.

General Procedure for Preparation of Oxa-ibogaine Analogs by lithiation/iodination and reductive Heck sequence.
Uncyclized intermediate **9a**/**9b** (1 equivalent) was dissolved in THF (0.5 M based on **9a**/**9b**) under argon atmosphere and the solution was cooled to -40°C in acetone/dry ice bath. Solution of n-butyl lithium in hexanes (2.5 M, 2 equivalents) was added dropwise over 5 – 10 min and the resulting orange solution was stirred 1 h at -40°C. Solution of iodine (1.5 - 1.7 equivalents) in THF (same volume as for **9a**/**9b**) was added (I_2 is consumed during addition, but persistent coloration remains after the entire amount is added) and after stirring for 10 min at -40°C the cooling bath was removed, and reaction allowed to warm to room temperature. After stirring at room temperature for 1 h, reaction was quenched with addition of saturated $Na_2S_2O_3$ solution (1 mL per 1 mmol of **9a**/**9b**) and the resulting mixture was vigorously stirred until excess iodine was consumed (org. phase discolored to pale orange-brown). Mixture was then poured into water, extracted with diethyl ether (3×), combined extracts were dried over Na_2SO_4, filtered and concentrated. Crude material (from **9a** pale orange, from **9b** orange-brown oil, can solidify over time) was used for next step without further purification. Largest scale tested for **9a** (5.05 mmol, 1.57 g) and **9b** (2.79 mmol, 0.87 g).
Note: Despite using excess n-BuLi and I_2 the reaction never reached full conversion, even when I_2 was added in excess to n-BuLi. Typically, 3 – 10% of unreacted starting material remains and is difficult to separate. No improvement in conversion was observed by increasing the reaction temperature after addition of n-BuLi to 0°C or using freshly opened commercially available anhydrous THF and n-BuLi solution.
Crude iodo-intermediate (1 eq.), sodium formate (4 eq., powdered and dried overnight on high-vacuum) and bis[tri(o-tolyl)phosphine]palladium(II) chloride (1 mol%, 0.01 eq.) were combined in dimethyl sulfoxide (0.25 M based on iodo-intermediate) under argon atmosphere. Closed reaction vessel was placed in a pre-heated (130 °C) heating adapter. Upon reaching reaction temperature (2 to 10 min, depending on volume and reaction vessel) the mixture (pale orange for **10a** or orange-brown for **10b**) turned dark brown/black. Reaction was further stirred at 130 °C for 5 - 60 min. After complete conversion was observed, reaction was cooled to room temperature and the dark mixture was poured to water (~5× volume of DMSO) and extracted with diethyl ether (3-4×, until no further extraction was observed). Combined extracts were dried over Na_2SO_4, filtered and concentrated. Obtained crude material was purified as indicated for each derivative.
*Note: In the course of reaction scale-up, it was observed that starting material is consumed upon reaching reaction temperature (exact time depends on reaction volume and vessel used). Subsequently, reaction time was reduced to 5 minutes after color change was observed due to precipitation of reduced palladium. Two repeats for oxa-ibogaine **10a** suggest possible improvement in yield by shortening the reaction time or lowering the reaction temperature. Palladium loading <1 mol% was not tested at this time.*

Oxa-ibogaine (10a)
Prepared according to the general procedure, reaction was repeated 3 times on (1.88, 2.0 and 5.05 mmol scale). The crude material after cyclization was purified by column chromatography (30:1 hexanes:EtOAc + 1% Et_3N) to yield the product as an off white solid. The slightly impure material, typically (>95% purity) was used for next step as is, yield of first repeat over two steps (0.47 g, 80%), reaction time 60 min. For second (0.52 g 86%) and third repeat (1.42 g, 90%), reactions were terminated 5 minutes after reduction of palladium was observed, total reaction time <15 minutes. Yields are corrected for presence of uncyclized material **9a** that can be removed after transformation to **11a,** as indicated in the multigram scale Ni-based synthesis note.
Spectral characterization of **10a** is identical to the material prepared by Ni mediated cyclization reaction.

Epi-oxa-ibogaine (10b)
Prepared according to the general procedure, reaction was repeated 3 times on (1.0, 1.03, and 6.74 mmol scale). The crude material after cyclization was purified by column chromatography (gradient of 15 to 20% EtOAc in hexanes + 2% Et_3N) to yield the product as an off white solid. Yields over two steps (81%, 252 mg) and (250 mg, 78%). Third repeat (1.32 g, 63%), initial palladium catalyst was reduced upon reaching reaction temperature, but no conversion of iodo-intermediate was detected. Full conversion was achieved by the addition of extra 4 equiv. HCOONa and 1 mol% of $Pd[P(o\text{-tolyl})_3]_2Cl_2$ to the same reaction mixture and continued heating for 15 min. During purification a mixed fraction containing 83:17 **9b**:**10b** was also recovered (0.37 g, 19%). Deviation from previous repeats were higher reaction scale, different batch of solvent and reagents (SM, HCOONa and Pd cat. were pre-dried for 1 h before addition of solvent at 40°C instead of only HCOONa overnight at room temperature).
Spectral characterization of **10b** is identical to the material prepared by Ni mediated cyclization reaction.

Synthesis Comments
The lithiation/iodination and reductive Heck sequence represents a superior alternative for multigram synthesis of oxa-iboga compounds compared to alternative, previously published methodologies: Ni-catalyzed C–H activation (catalyst and ligand are oxygen sensitive, require high loading and provide modest yields for certain substrates)[11] or electrophilic palladation–cyclization (low to modest yields – substrate dependent, stoichiometric Pd reagent required).[11,12] The development of the reductive Heck sequence for oxa-iboga compounds was inspired by conditions applied for synthesis of indole-based iboga analogs.[13]

X-Ray structure determination of Oxa-ibogaine 10a

X-Ray suitable crystals were prepared by dissolving racemic oxa-ibogaine in hot acetonitrile and the resulting solution was allowed to cool to room temperature and slowly concentrate by evaporation.

**Property**	**Oxa-ibogaine 10a**	**Property**	**Oxa-ibogaine 10a**
Formula	C20H25NO2	*Radiation type*	MoK α
Formula Weight	311.41	*Wavelength (λ, Å)*	0.71073
$D_{calc.}$/ $g{\cdot}cm^{-3}$	1.272	*θ min, deg.*	2.33
Mu/mm^{-1}	0.081	*θ max, deg.*	30.55
Color	colorless	*Measured Refl.*	25983
Shape	block	*Independent Refl.*	4982
$Size/mm^3$	0.43×0.11×0.07	*Reflections with I > 2(I)*	3804
Space Group	$P2_1/n$	R_{int}	0.0432
Crystal System	Monoclinic	*Parameters*	210
a/Å	10.9618(18)	*Restraints*	0
b/Å	8.4800(14)	*Density Max*	0.434
c/Å	17.660(3)	*Density Min*	-0.211
α/°	90	*GoF*	1.045
β/°	97.742(3)	*wR2 (all data)*	0.1217
γ/°	90	*wR2*	0.1100
$V/Å^3$	1626.6(5)	*R1 (all data)*	0.0625
Z	4	*R1*	0.0441
Temperature (K)	150(2)		

Appendix 1. High Resolution Mass Spectra

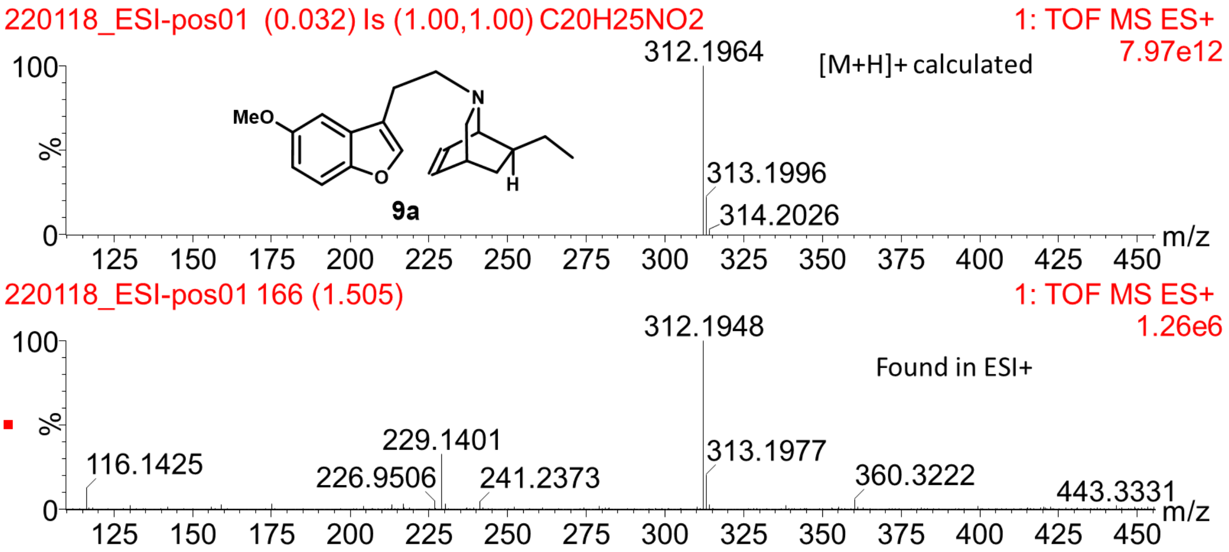

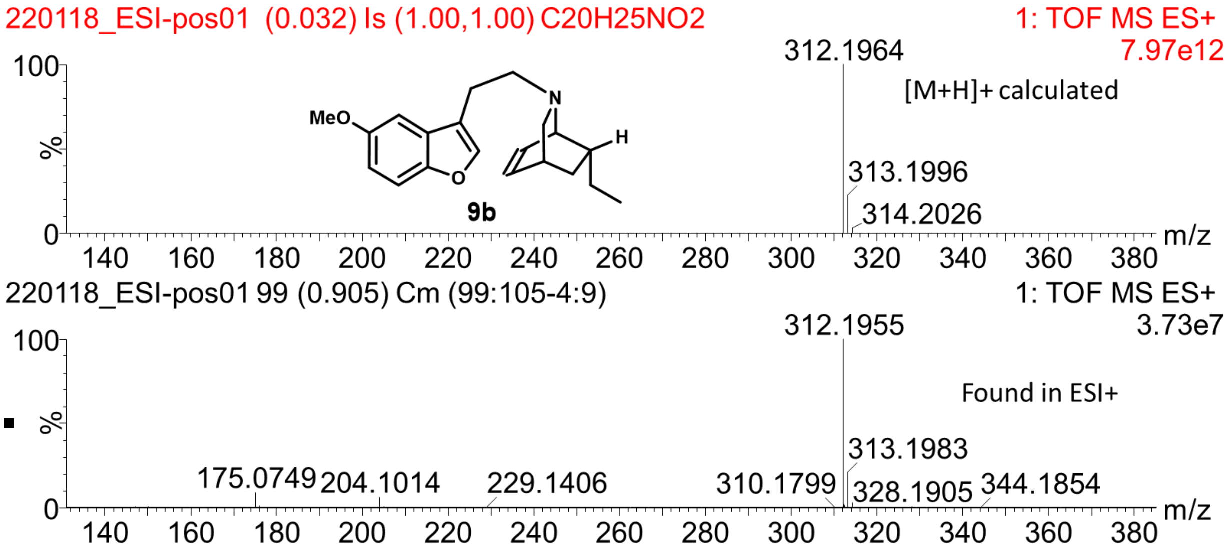

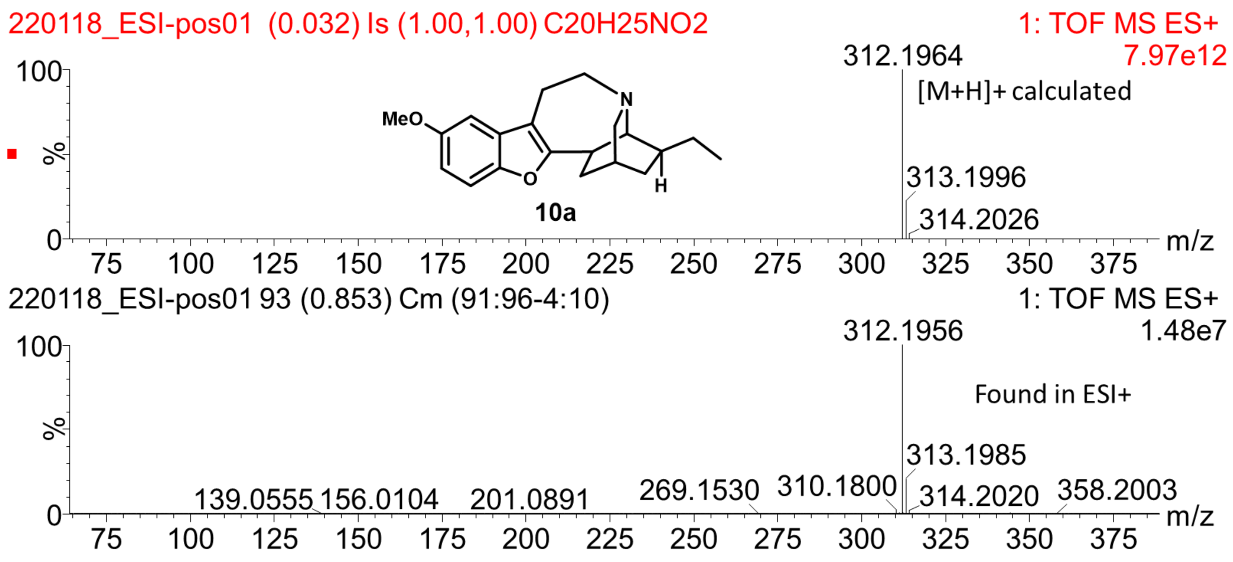
220118_ESI-pos01 (0.032) Is (1.00,1.00) C20H25NO2
1: TOF MS ES+
312.1964
7.97e12
[M+H]+ calculated
MeO
N
O
H
10a
313.1996
314.2026
100
%
0
m/z
75 100 125 150 175 200 225 250 275 300 325 350 375
220118_ESI-pos01 93 (0.853) Cm (91:96-4:10)
1: TOF MS ES+
312.1956
1.48e7
Found in ESI+
313.1985
139.0555
156.0104
201.0891
269.1530
310.1800
314.2020
358.2003

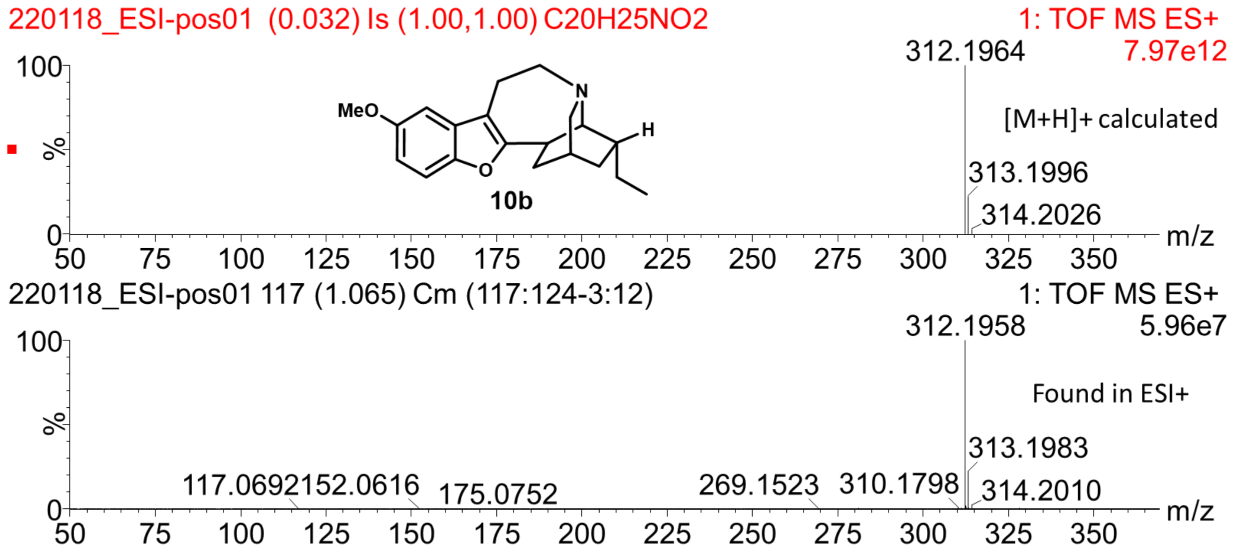
220118_ESI-pos01 (0.032) Is (1.00,1.00) C20H25NO2
1: TOF MS ES+
312.1964
7.97e12
MeO
N
H
O
10b
[M+H]+ calculated
313.1996
314.2026
100
%
0
m/z
50 75 100 125 150 175 200 225 250 275 300 325 350
220118_ESI-pos01 117 (1.065) Cm (117:124-3:12)
1: TOF MS ES+
312.1958
5.96e7
Found in ESI+
313.1983
117.0692
152.0616
175.0752
269.1523
310.1798
314.2010

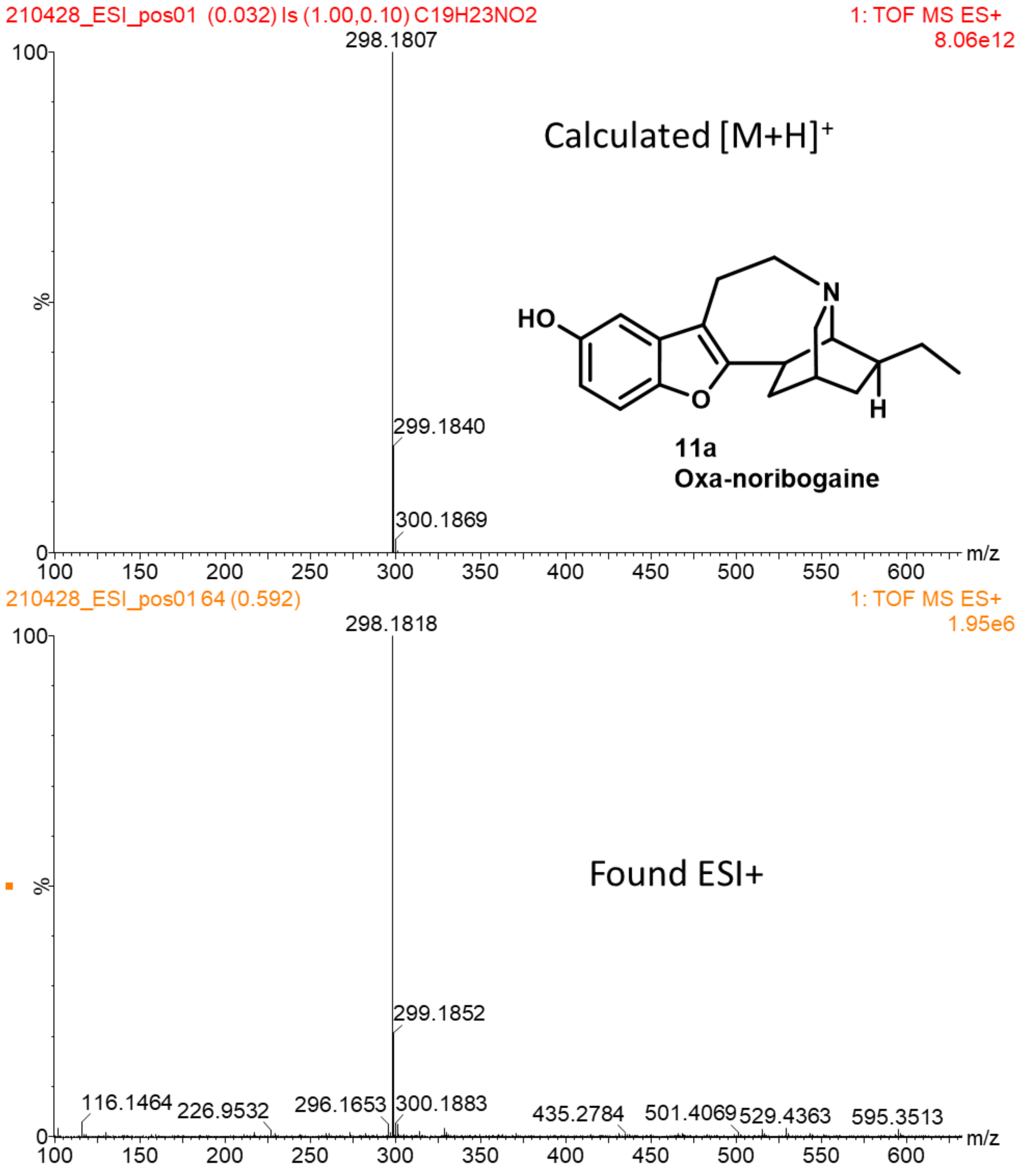
210428_ESI_pos01 (0.032) Is (1.00,0.10) C19H23NO2
1: TOF MS ES+
8.06e12
298.1807
Calculated [M+H]+
299.1840
300.1869
HO
N
O
H
11a
Oxa-noribogaine
m/z
210428_ESI_pos01 64 (0.592)
1: TOF MS ES+
1.95e6
298.1818
Found ESI+
299.1852
116.1464
226.9532
296.1653
300.1883
435.2784
501.4069
529.4363
595.3513
%
100
0
100
150
200
250
300
350
400
450
500
550
600

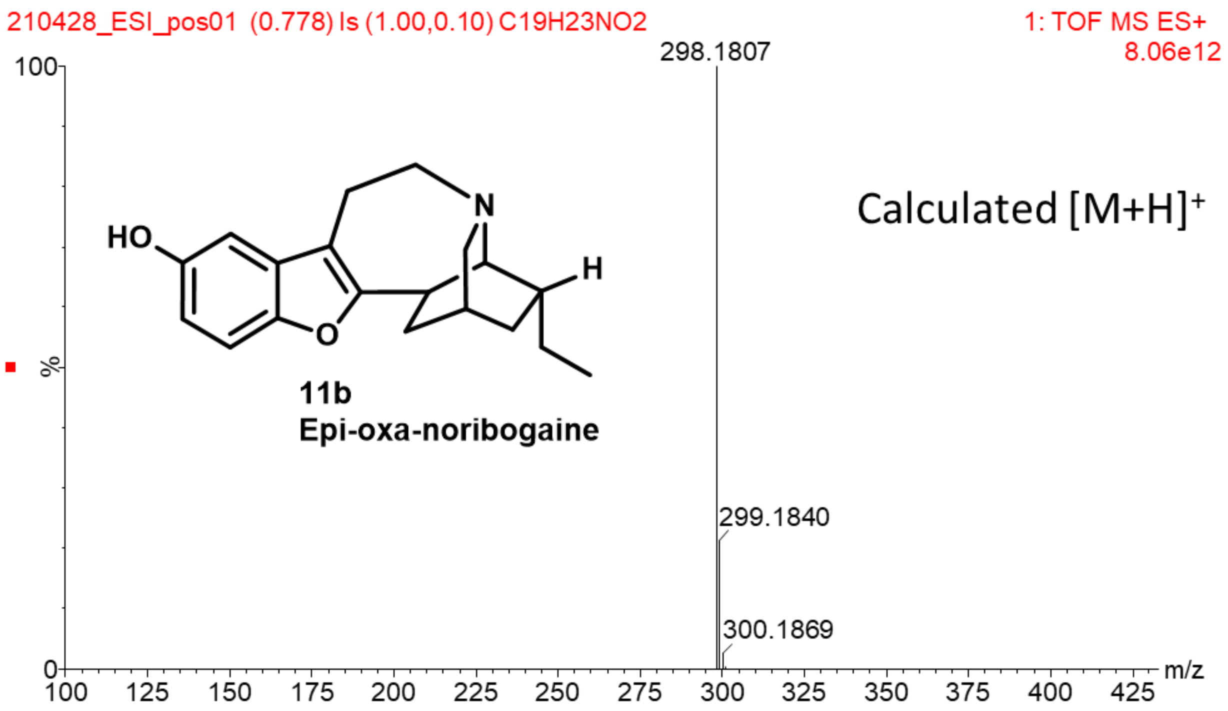
210428_ESI_pos01 (0.778) Is (1.00,0.10) C19H23NO2
1: TOF MS ES+
8.06e12
298.1807
299.1840
300.1869
Calculated [M+H]+
HO
N
H
O
11b
Epi-oxa-noribogaine
100
%
0
100 125 150 175 200 225 250 275 300 325 350 375 400 425
m/z

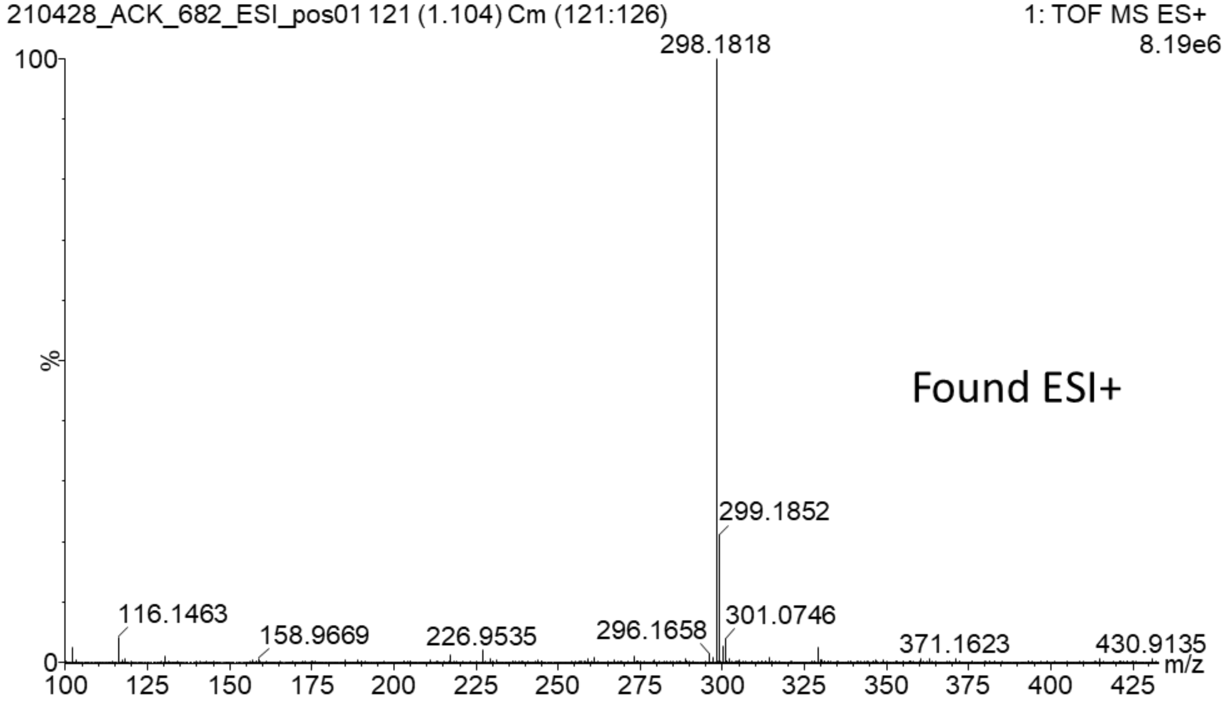
210428_ACK_682_ESI_pos01 121 (1.104) Cm (121:126)
1: TOF MS ES+
8.19e6
298.1818
299.1852
116.1463
158.9669
226.9535
296.1658
301.0746
371.1623
430.9135
Found ESI+
100
%
0
100 125 150 175 200 225 250 275 300 325 350 375 400 425
m/z

Appendix 2. NMR Spectra

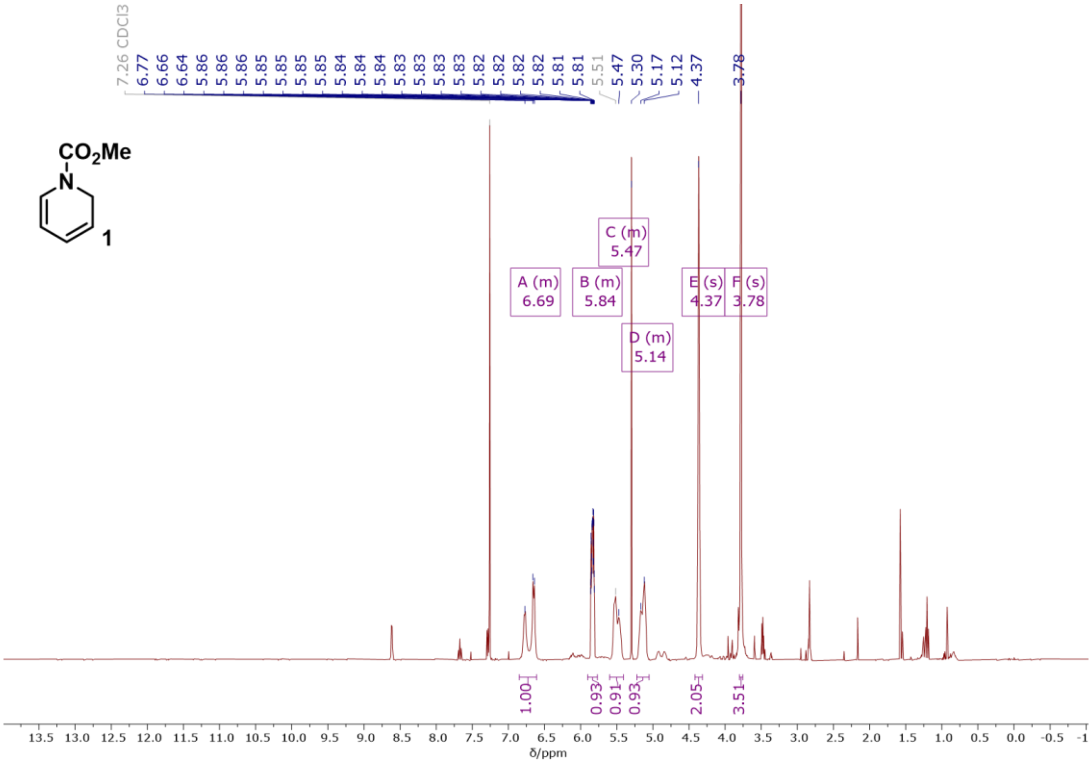

^{1}H NMR (400 MHz, $CDCl_3$) spectrum of a crude, unstable intermediate **1**.

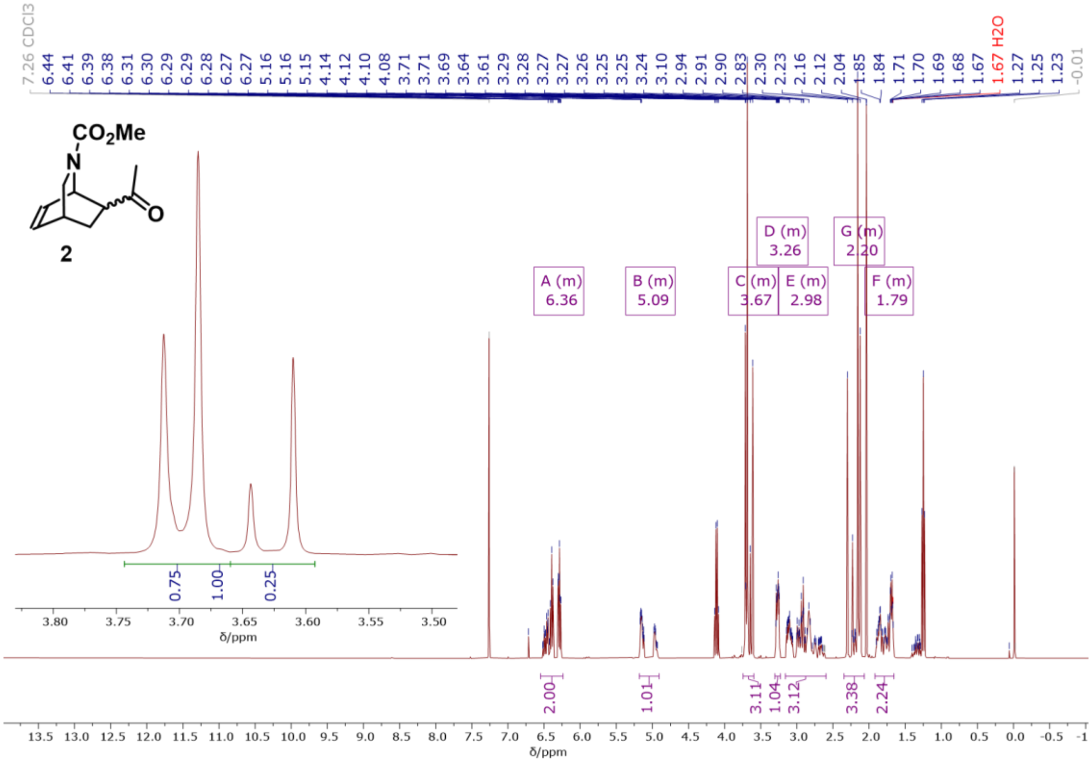

^{1}H NMR (400 MHz, $CDCl_3$) spectrum of a mixture of **2a** and **2b** prior to epimerization.

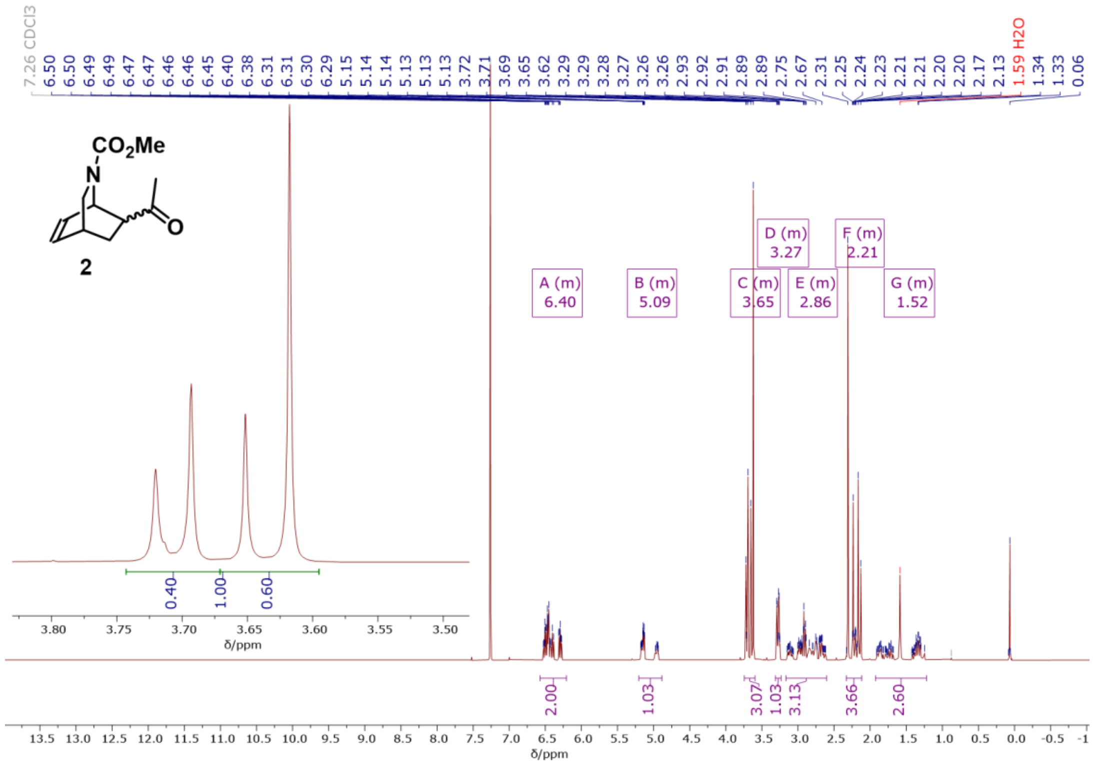

^1H NMR (400 MHz, $CDCl_3$) spectrum of a mixture of **2a** and **2b** after epimerization.

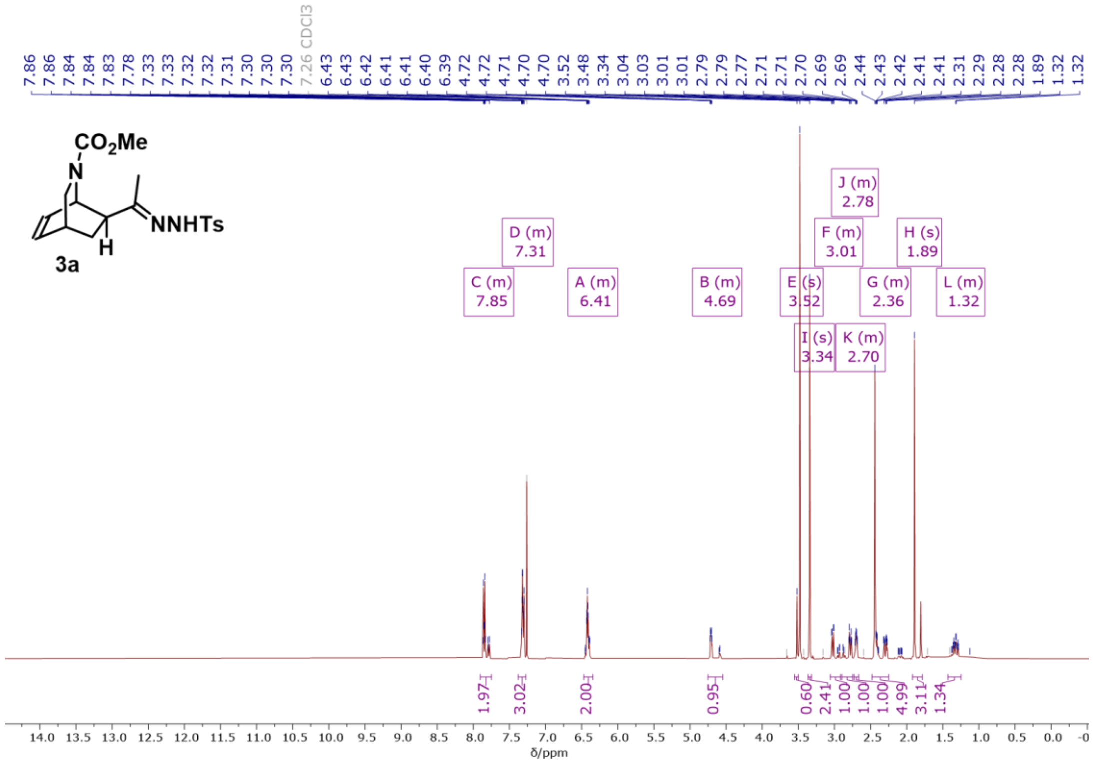

^1H NMR (400 MHz, $CDCl_3$) spectrum of compound 3**a.**

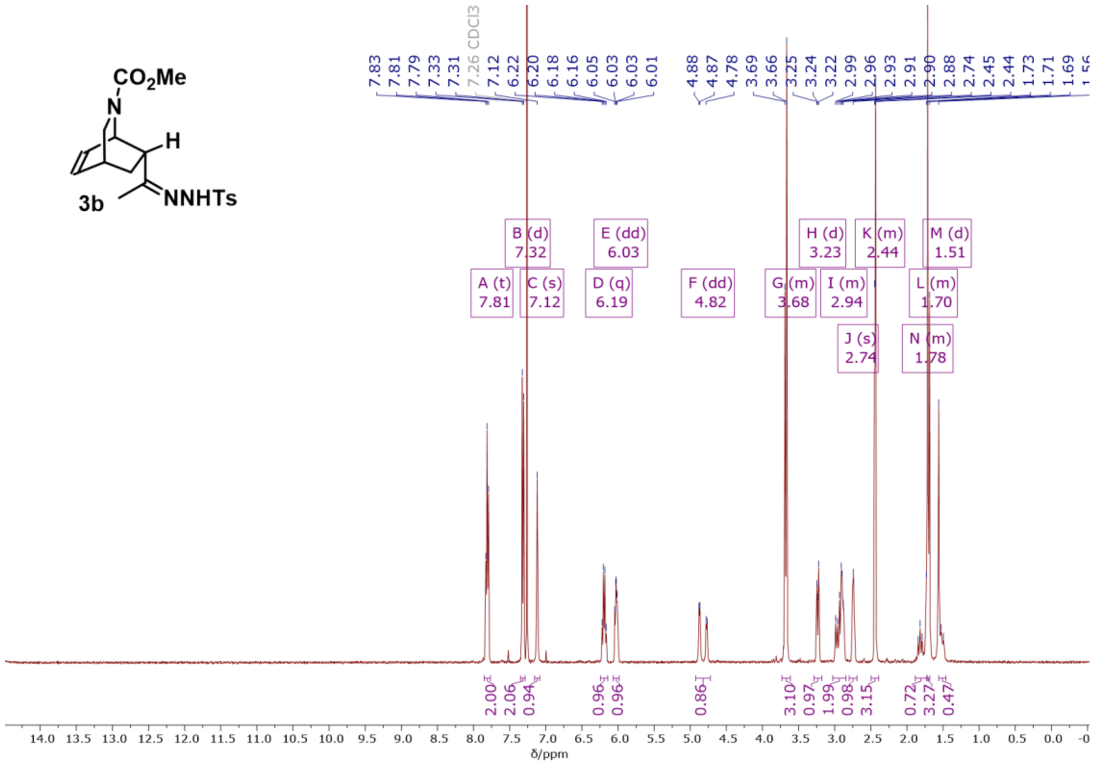

^{1}H NMR (400 MHz, $CDCl_3$) spectrum of compound 3**b.**

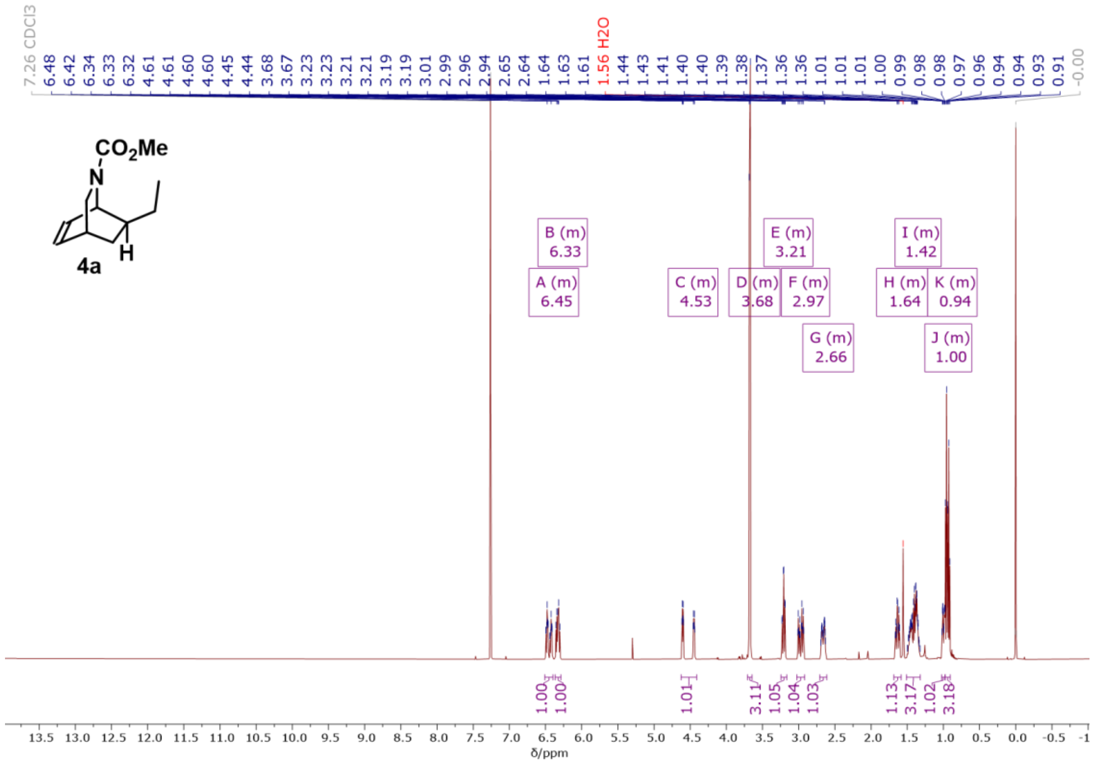

^{1}H NMR (500 MHz, $CDCl_3$) spectrum of compound 4**a.**

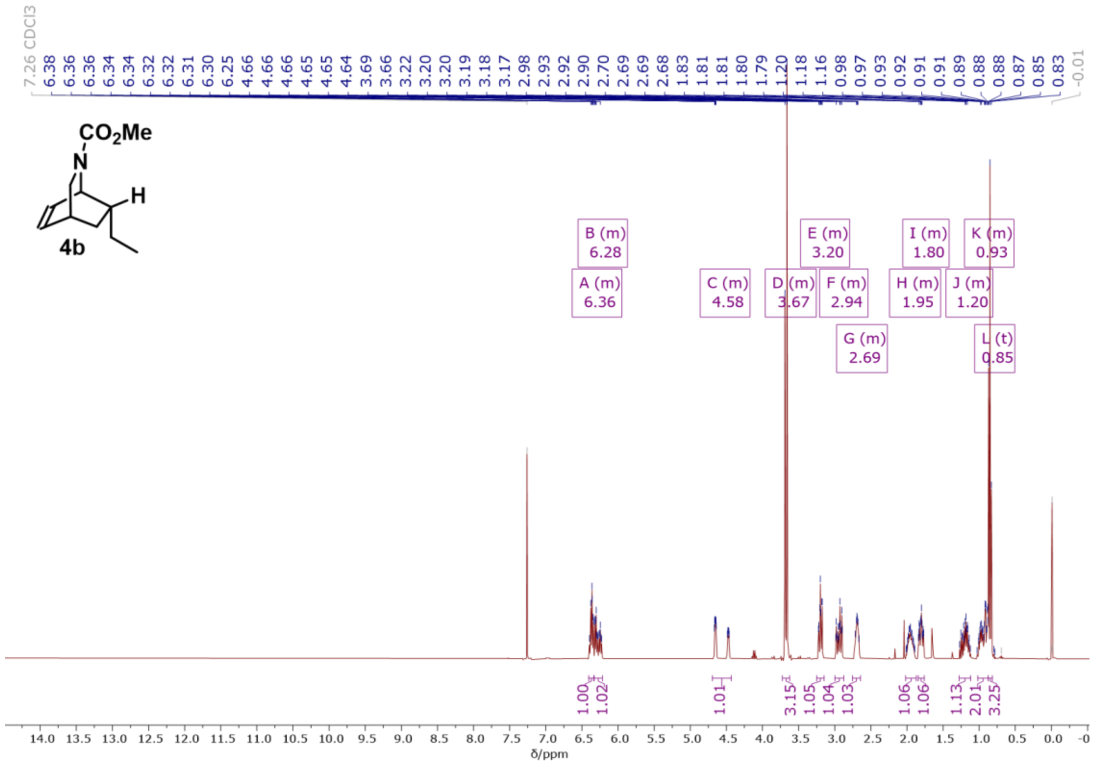

^{1}H NMR (400 MHz, $CDCl_3$) spectrum of compound 4**b**.

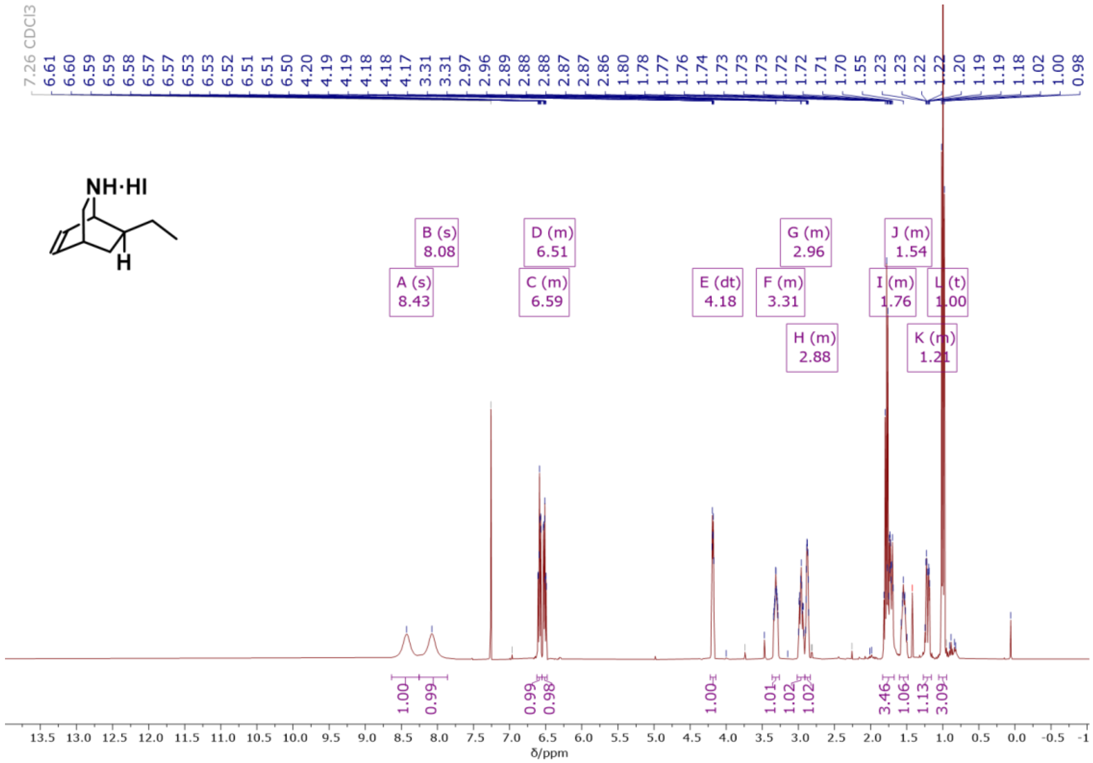

^{1}H NMR (400 MHz, $CDCl_3$) spectrum of exo-ethyl isoquinuclidine hydroiodide intermediate.

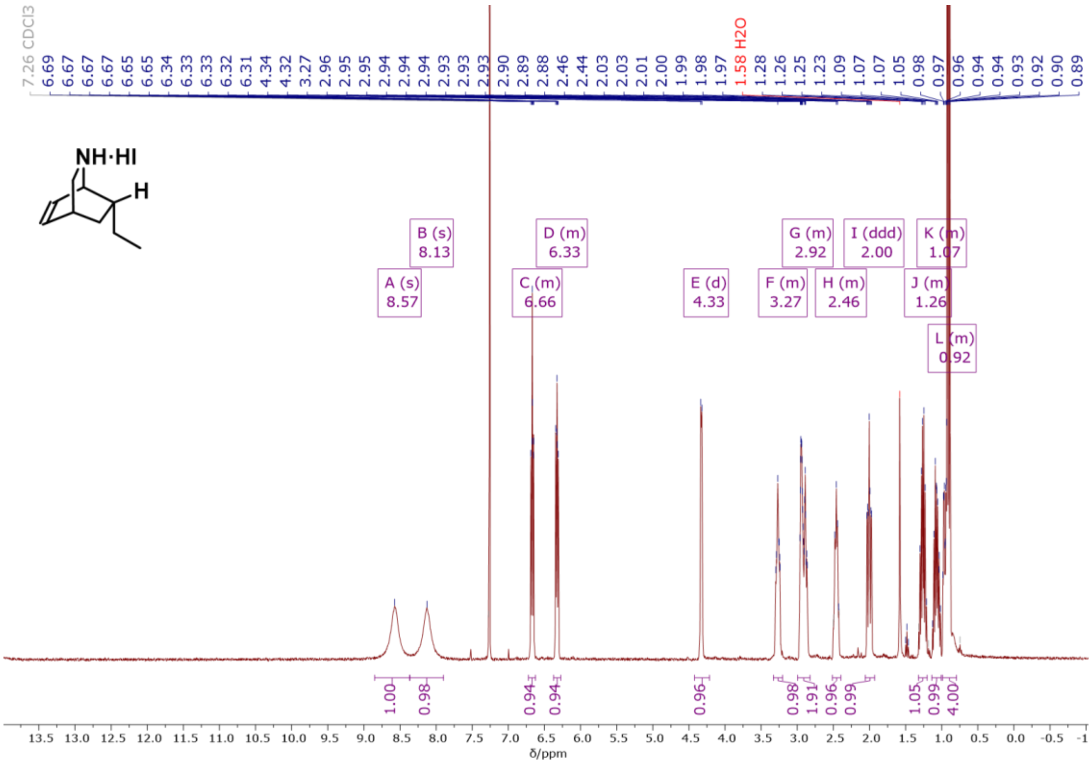

^{1}H NMR (400 MHz, $CDCl_3$) spectrum of endo-ethyl isoquinuclidine hydroiodide intermediate.

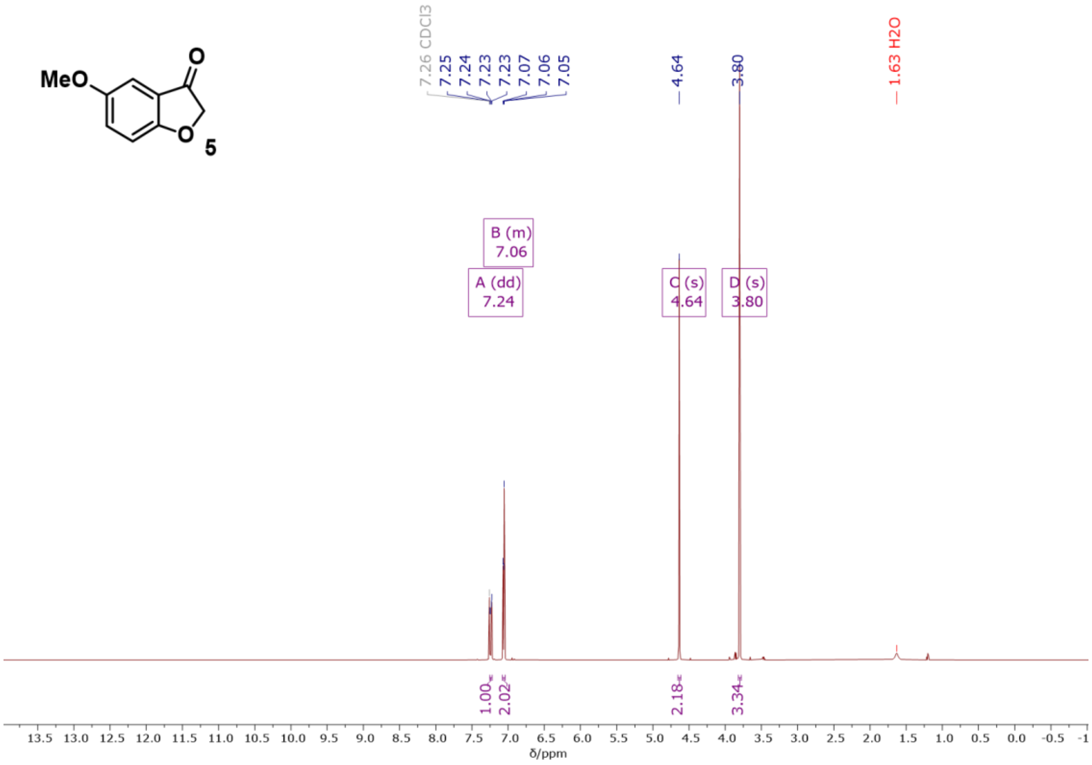

^{1}H NMR (400 MHz, $CDCl_3$) spectrum of compound **5**.

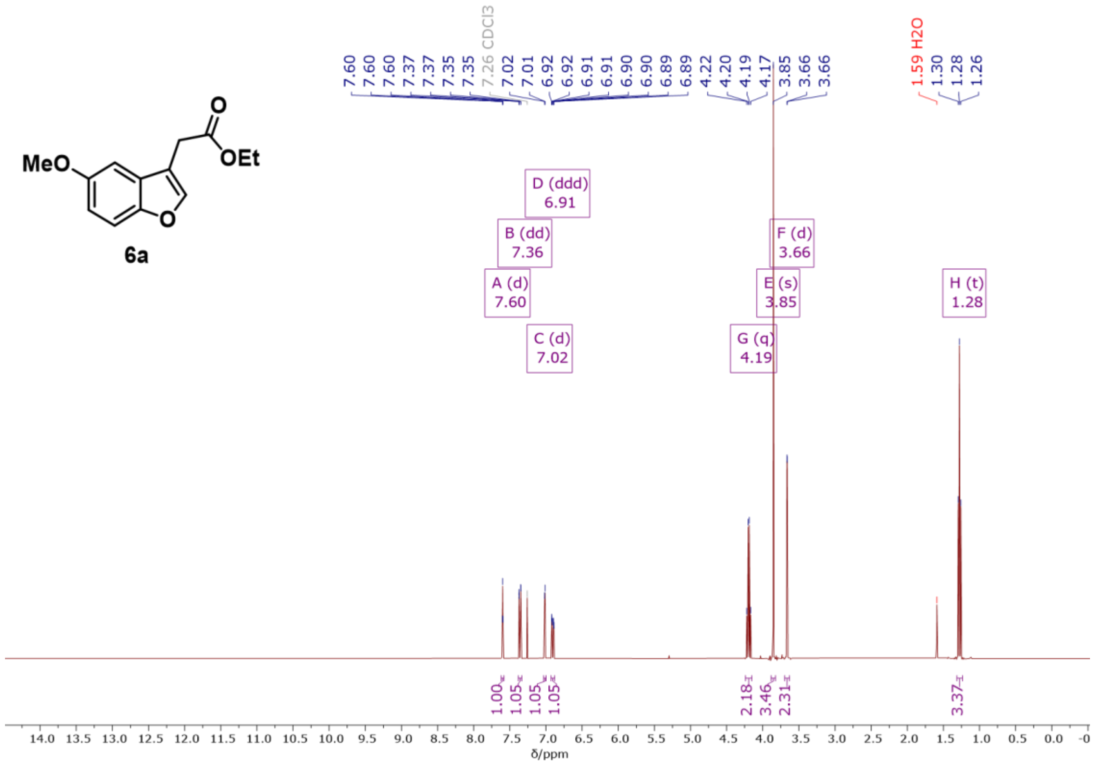

^{1}H NMR (400 MHz, $CDCl_3$) spectrum of compound **6a**.

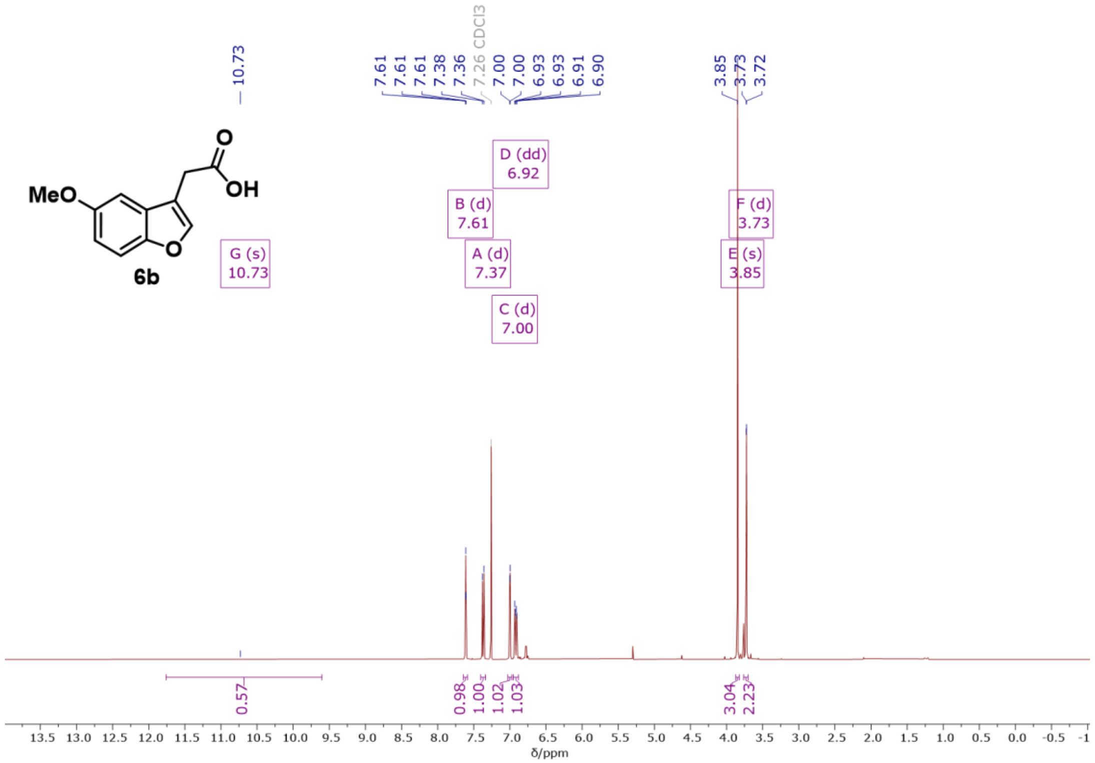

^{1}H NMR (400 MHz, $CDCl_3$) spectrum of crude compound **6b**.

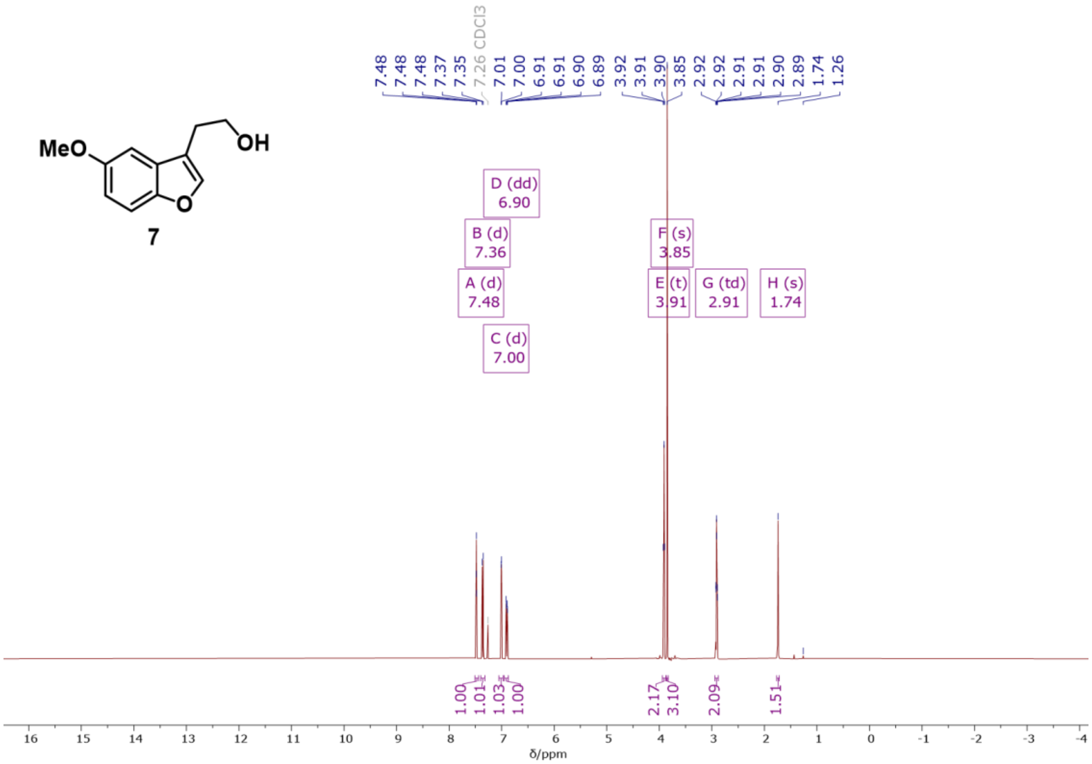

^{1}H NMR (500 MHz, $CDCl_3$) spectrum of compound **7**.

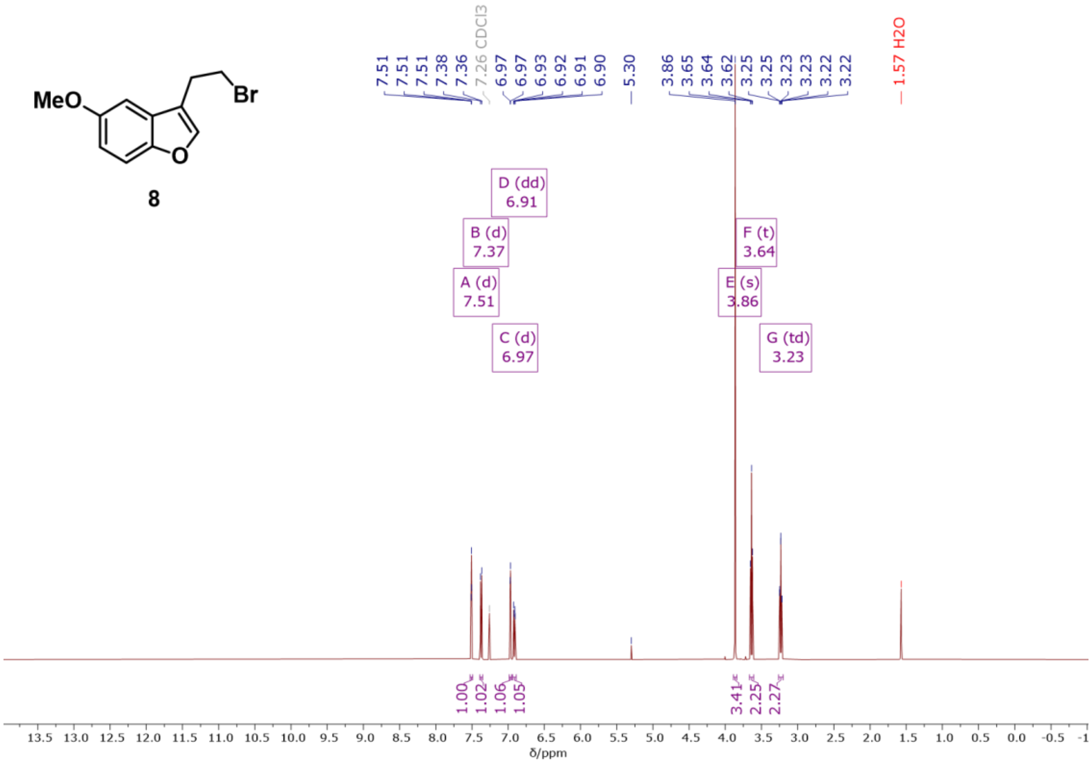

^{1}H NMR (500 MHz, $CDCl_3$) spectrum of compound **8**.

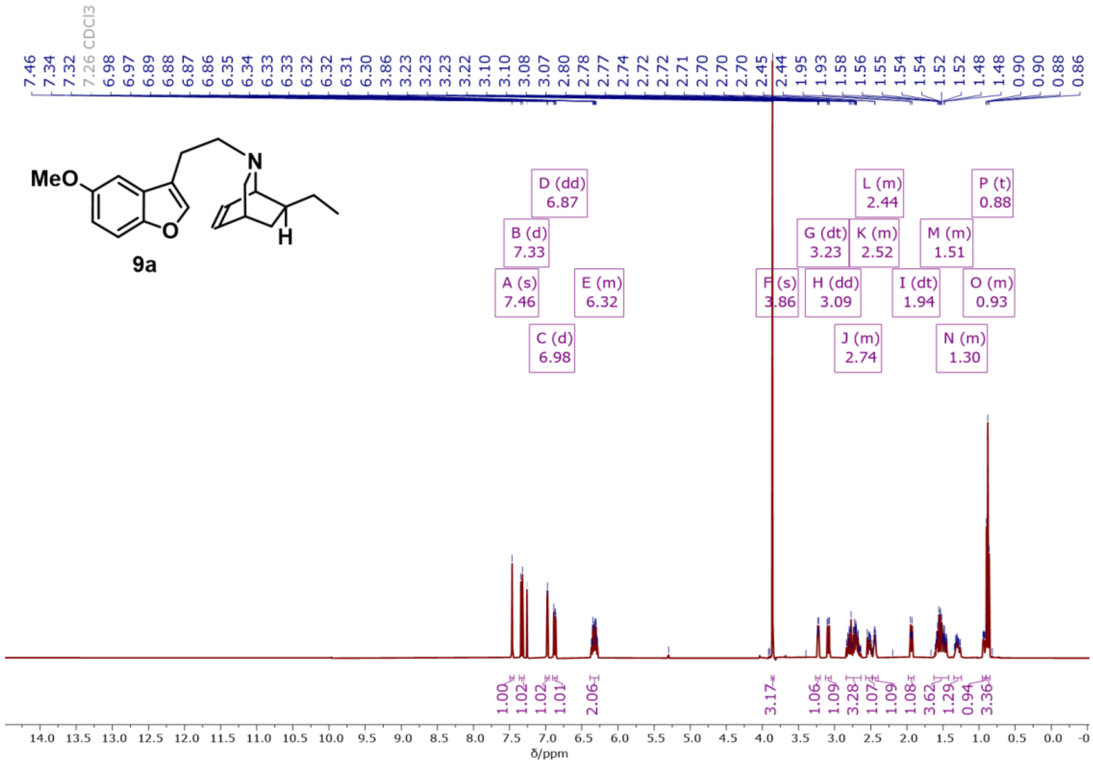

^1H NMR (400 MHz, $CDCl_3$) spectrum of compound **9a.**

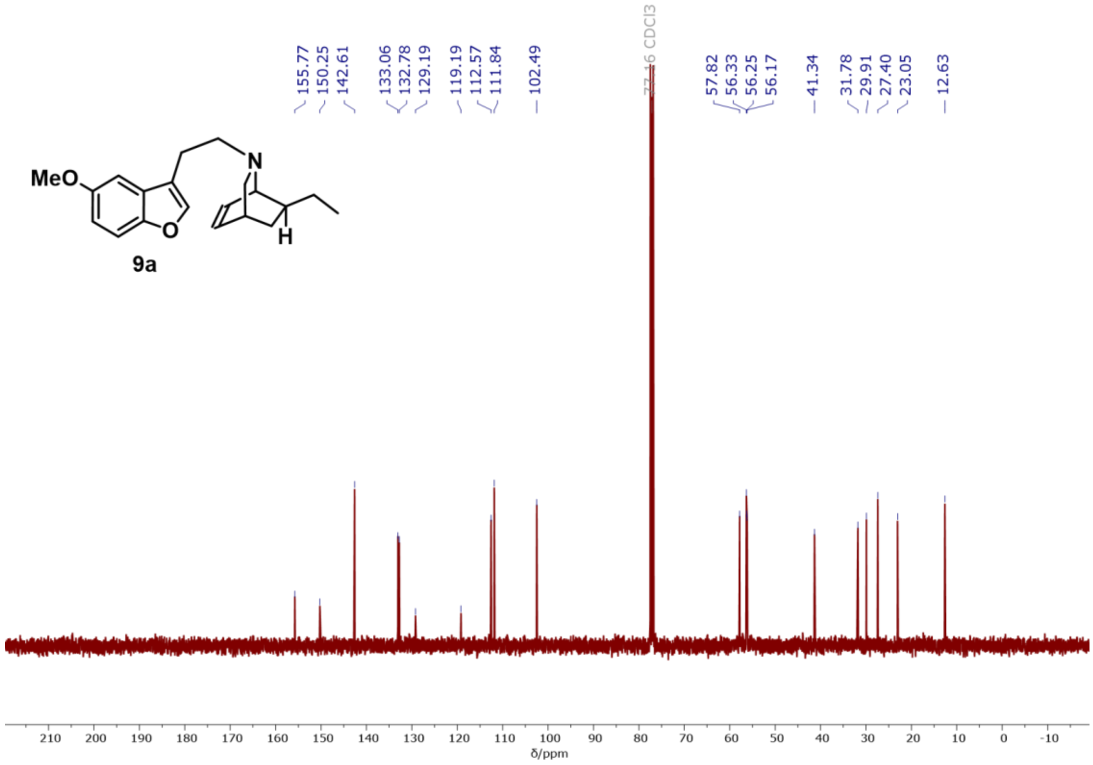

^{13}C NMR (101 MHz, $CDCl_3$) spectrum of compound **9a.**

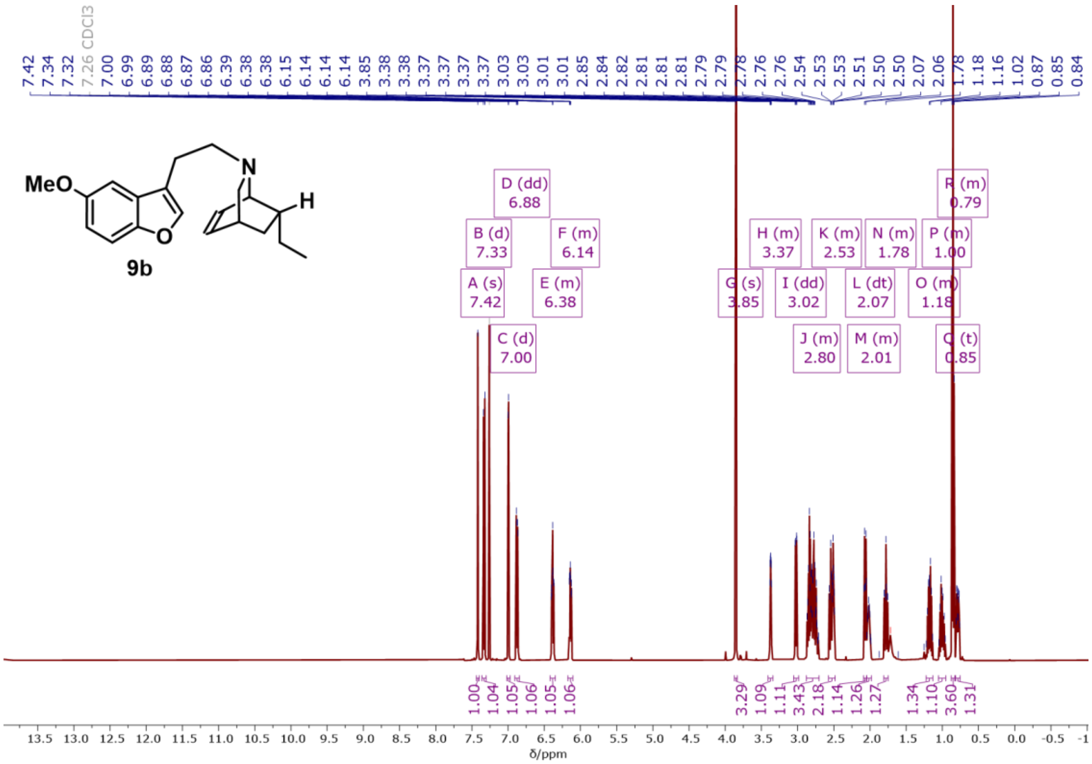

^{1}H NMR (400 MHz, $CDCl_3$) spectrum of compound **9b.**

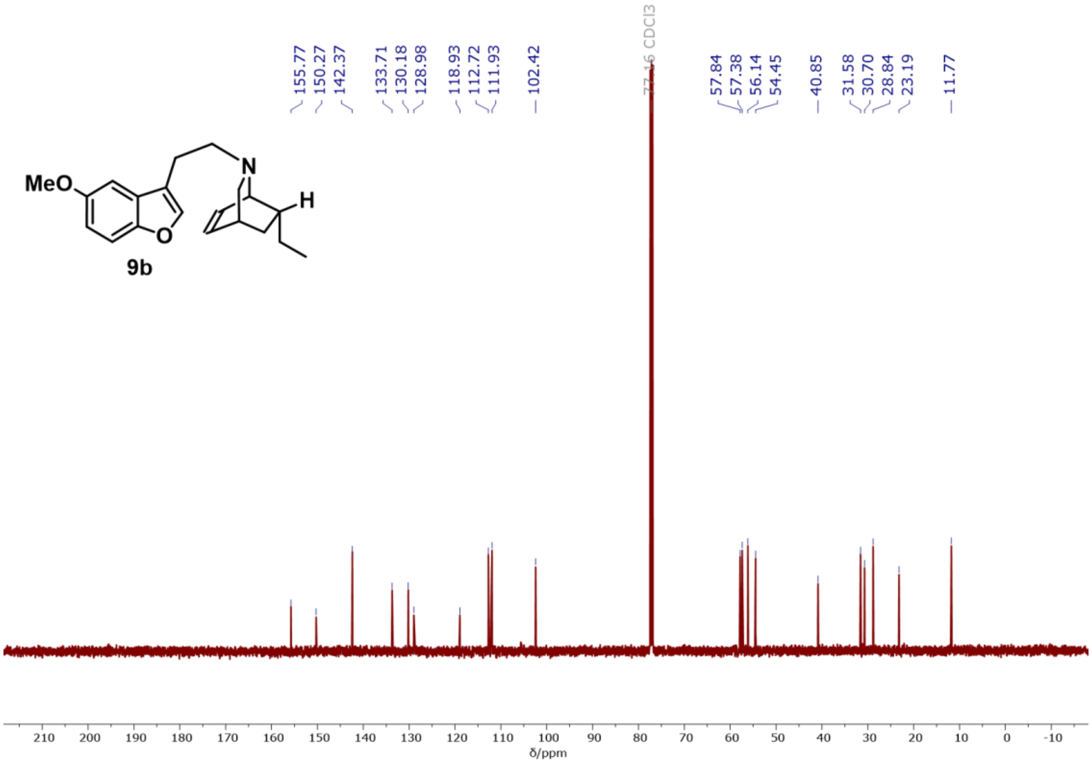

^{13}C NMR (101 MHz, $CDCl_3$) spectrum of compound **9b.**

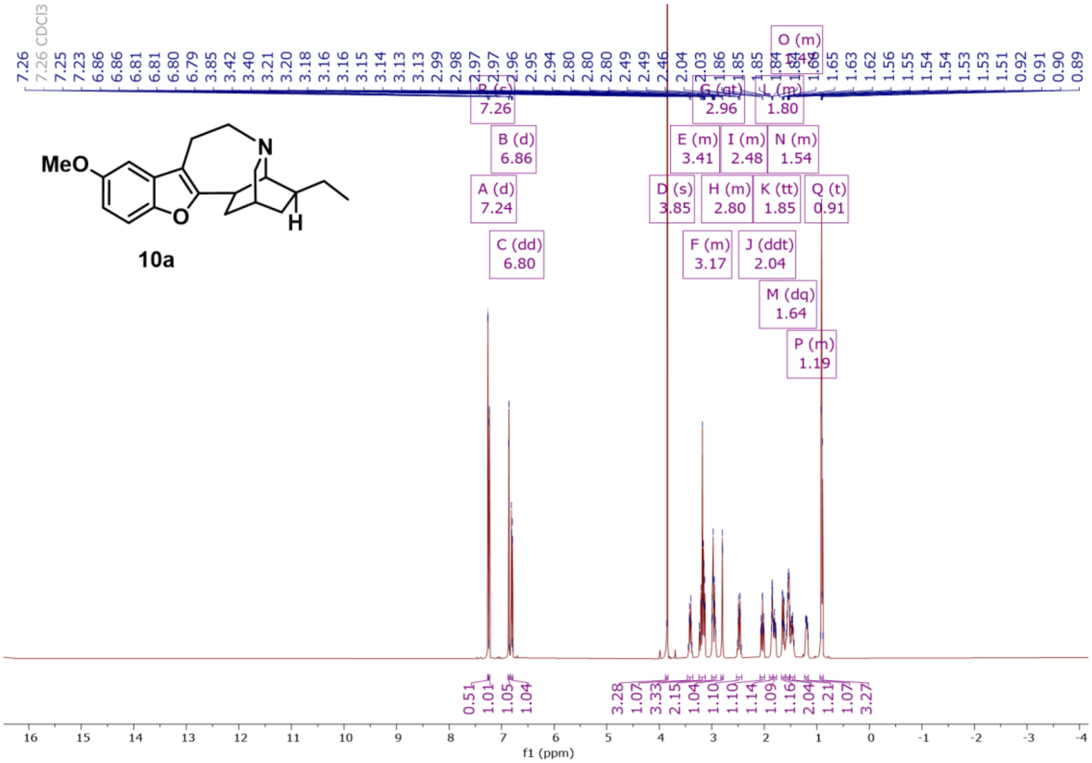

^{1}H NMR (500 MHz, $CDCl_3$) spectrum of compound **10a.**

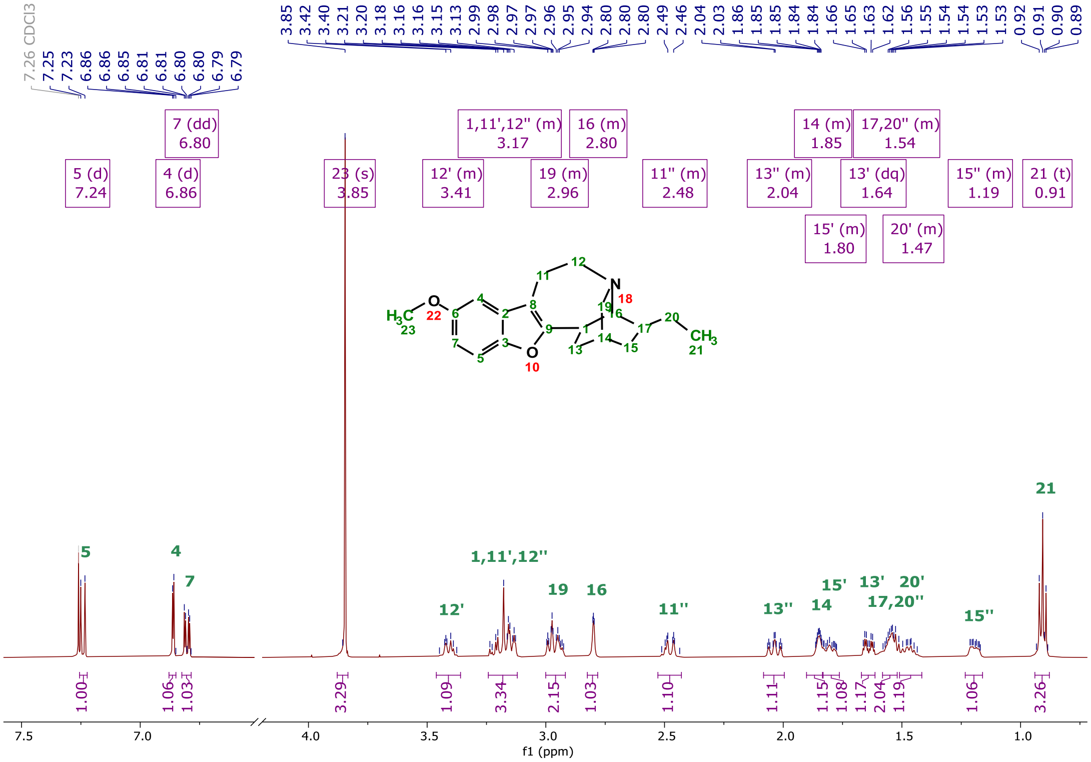

^{1}H NMR (500 MHz, $CDCl_3$) magnified and assigned spectrum of compound **10a**. Blank regions of spectra were omitted for clarity.

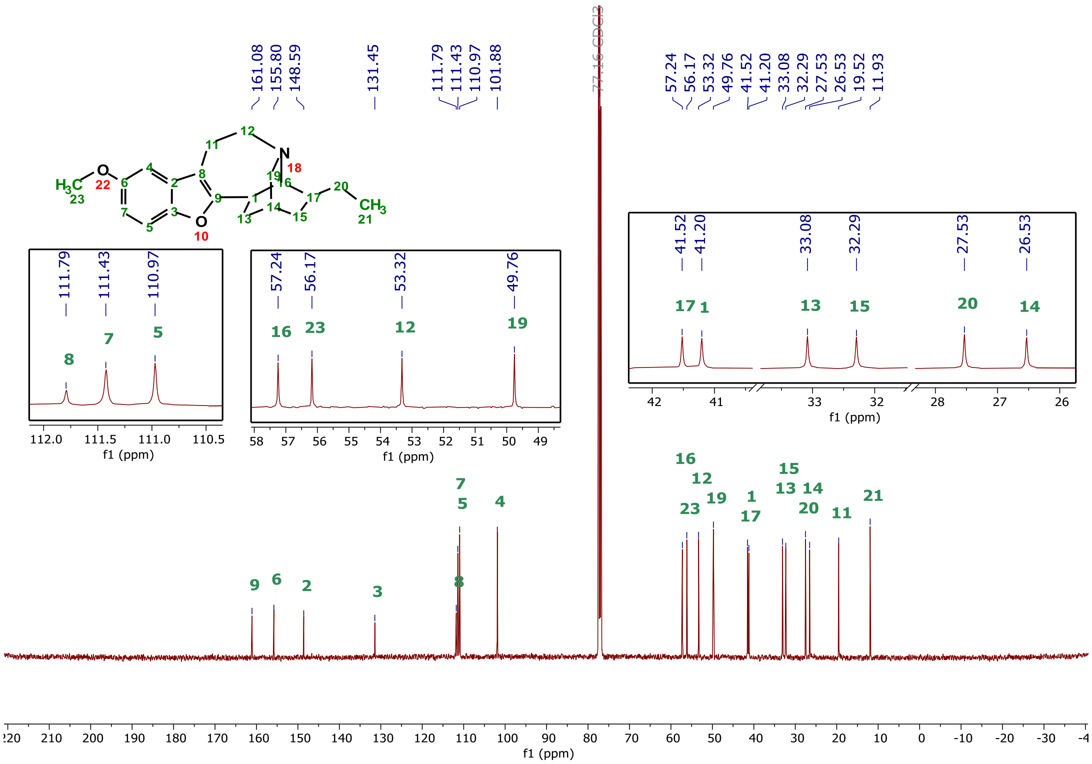

^{13}C NMR (126 MHz, $CDCl_3$) spectrum of compound **10a.** Expansions included for tightly clustered peaks.

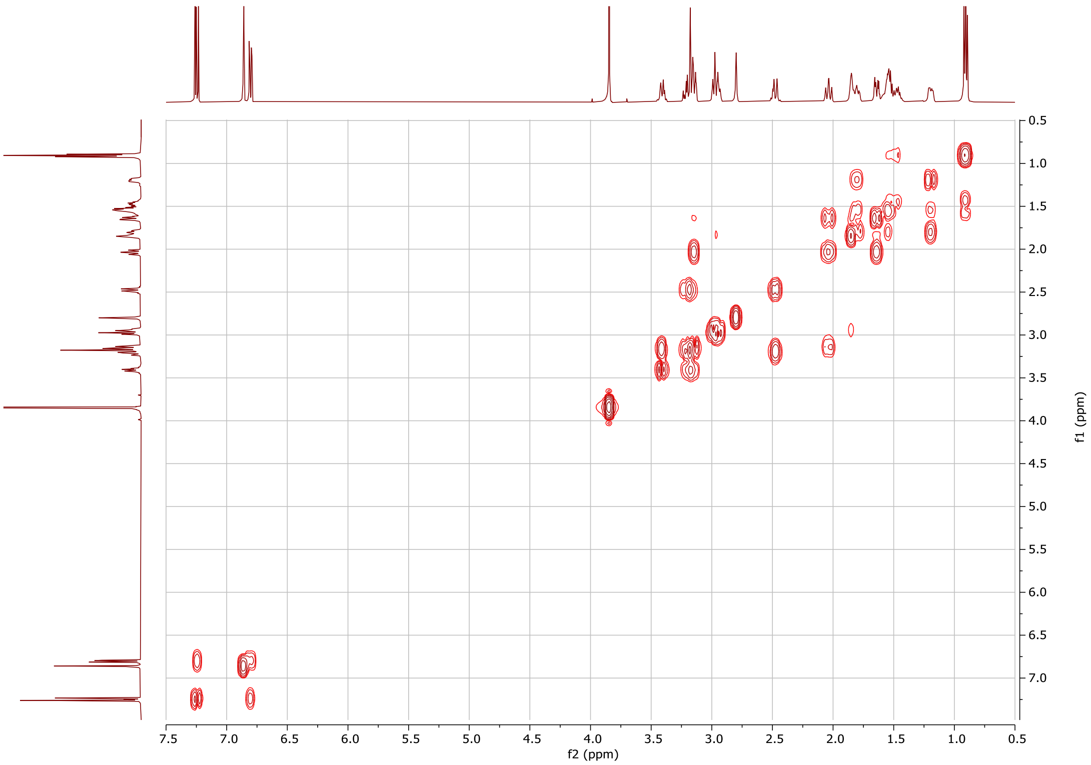

^{1}H-^{1}H COSY NMR (500 MHz, $CDCl_3$) spectrum of compound **10a.**

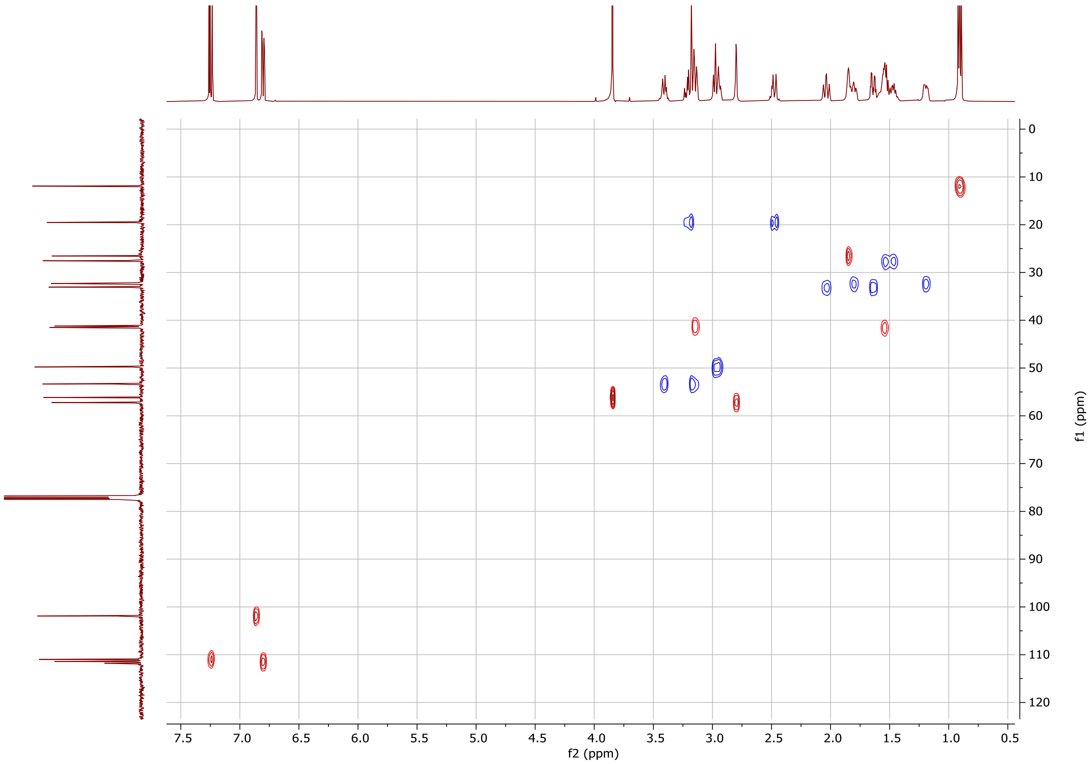

^{1}H-^{13}C HSQC multiplicity edited NMR (500/126 MHz, $CDCl_3$) spectrum of compound **10a.**

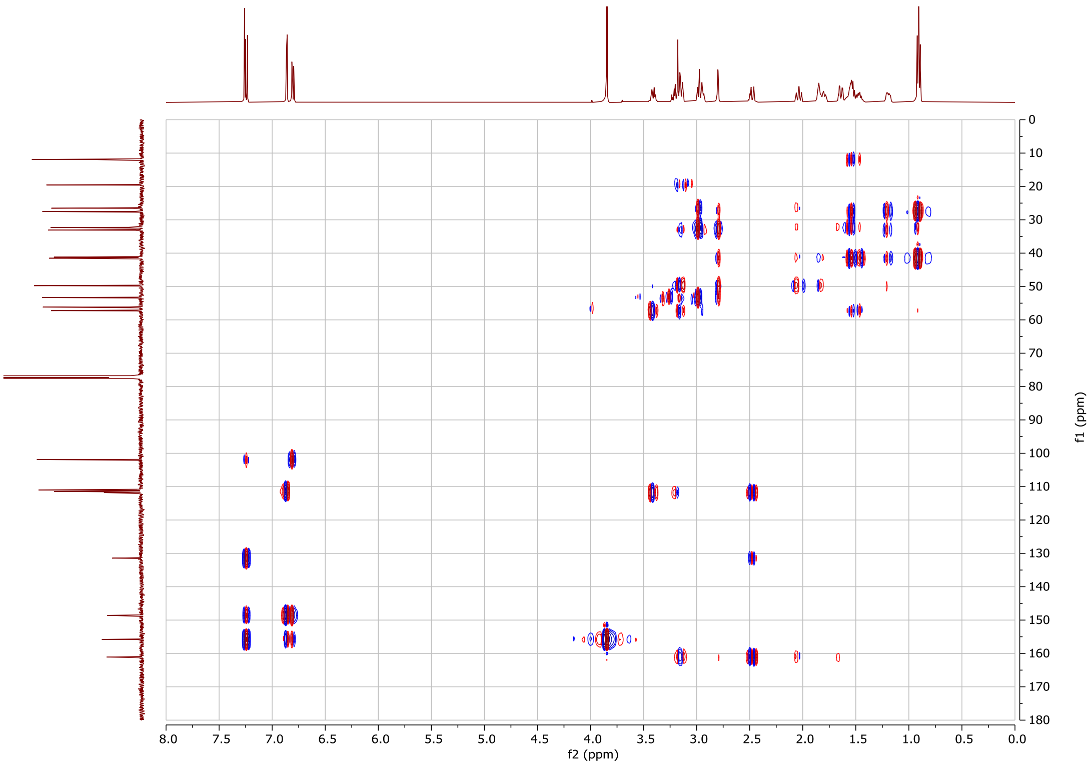

^{1}H-^{13}C HMBC NMR (500/126 MHz, $CDCl_3$) spectrum of compound **10a**, one-bond correlations are suppressed.

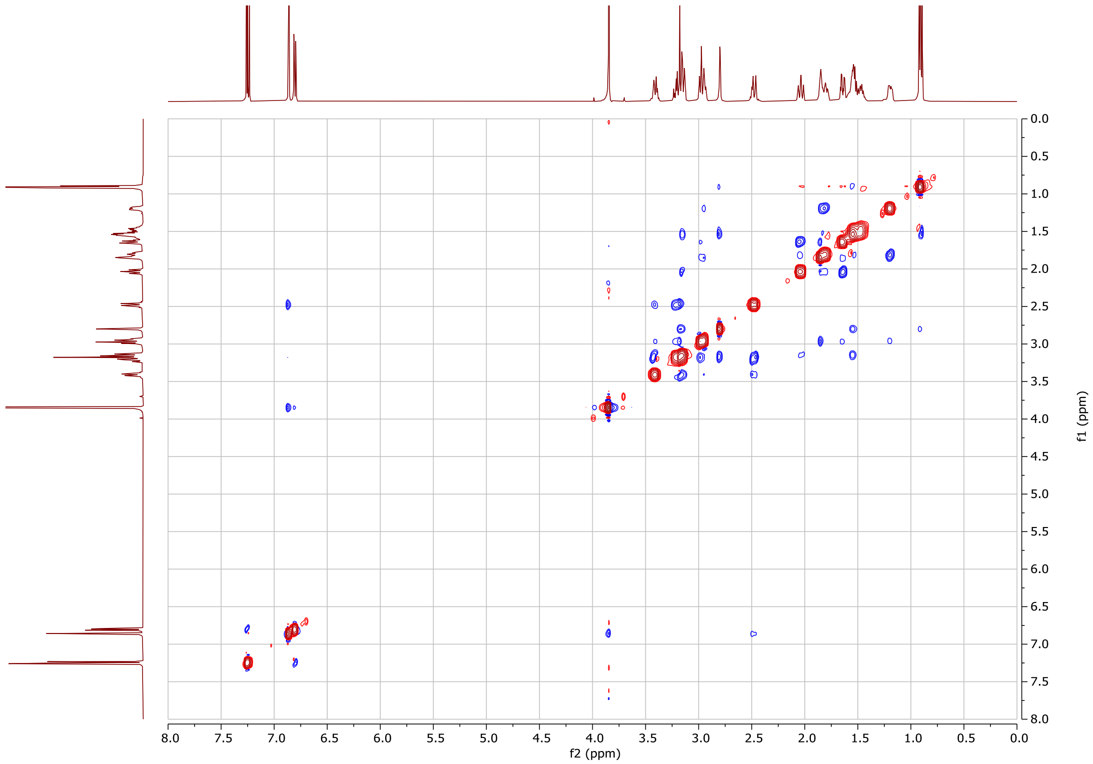

^{1}H-^{1}H NOESY NMR (500 MHz, $CDCl_3$) spectrum of compound **10a.**

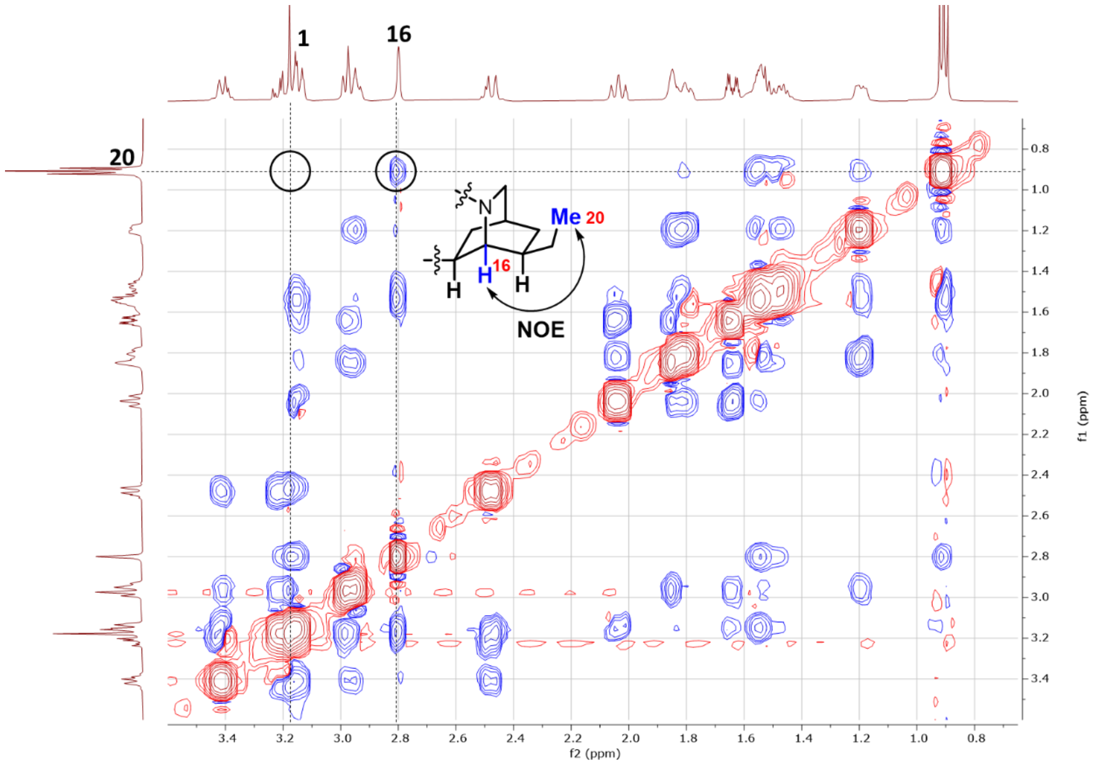

Isoquinuclidine portion of the NOESY spectrum of compound **10a.**

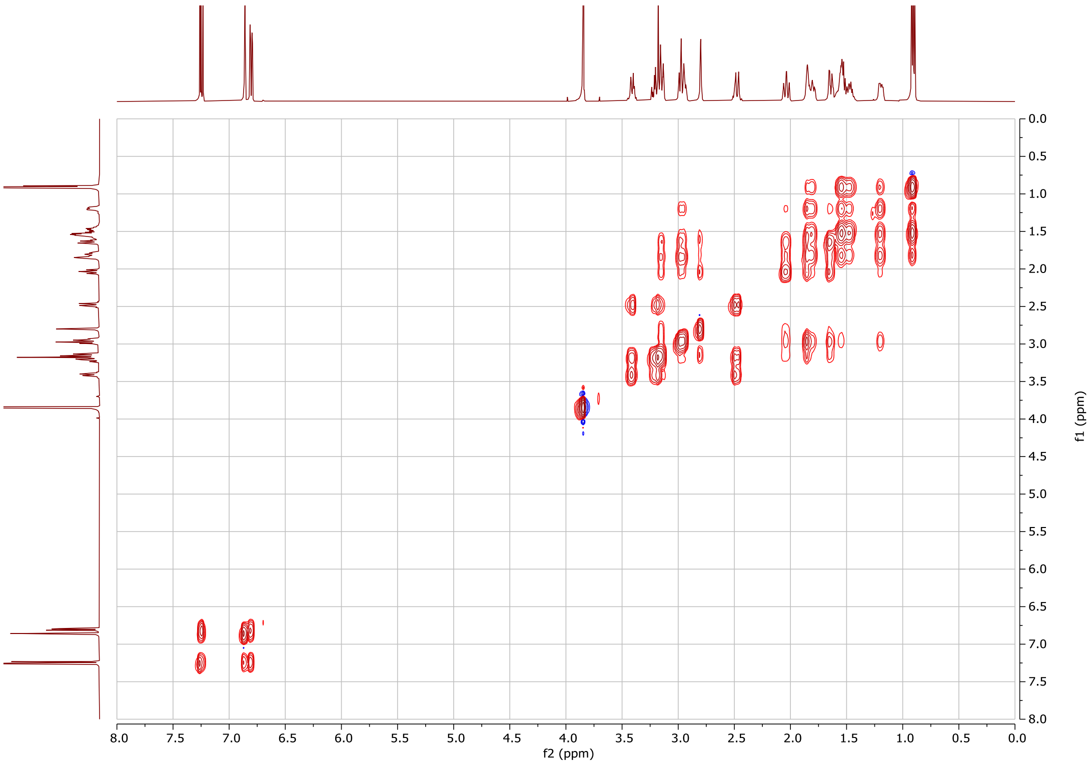

^{1}H-^{1}H TOCSY NMR (500 MHz, $CDCl_3$) spectrum of compound **10a.**

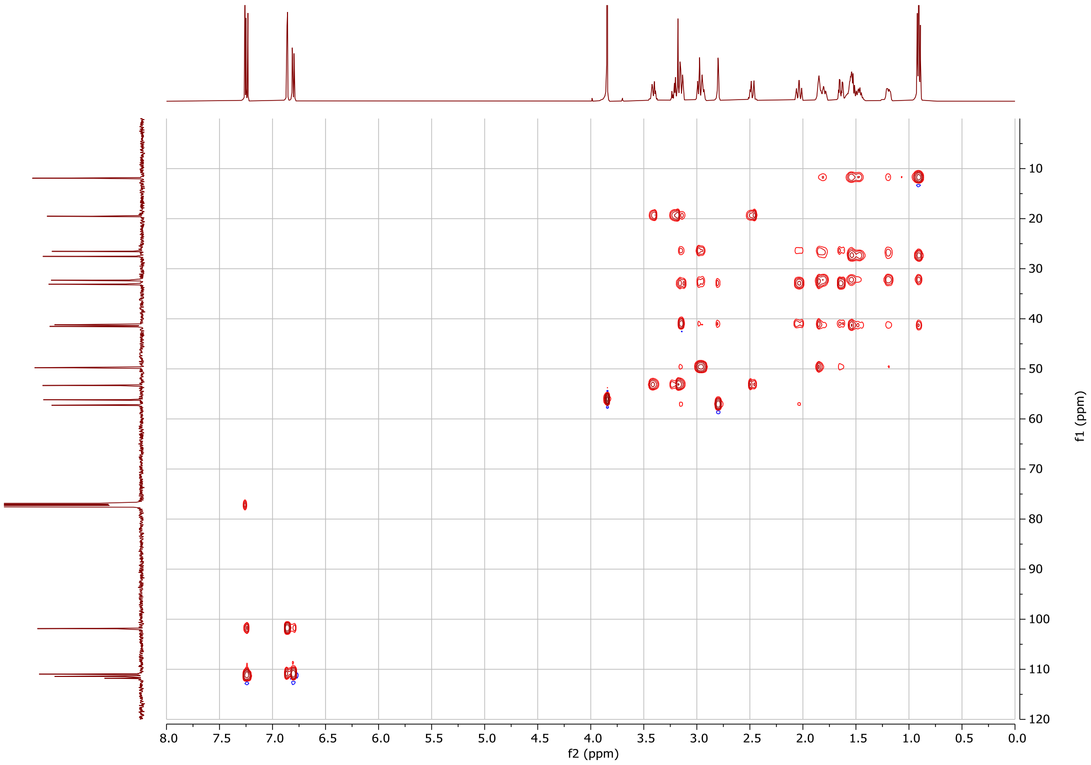

^{1}H-^{13}C HSQC-TOCSY NMR (500/126 MHz, $CDCl_3$) spectrum of compound **10a**.

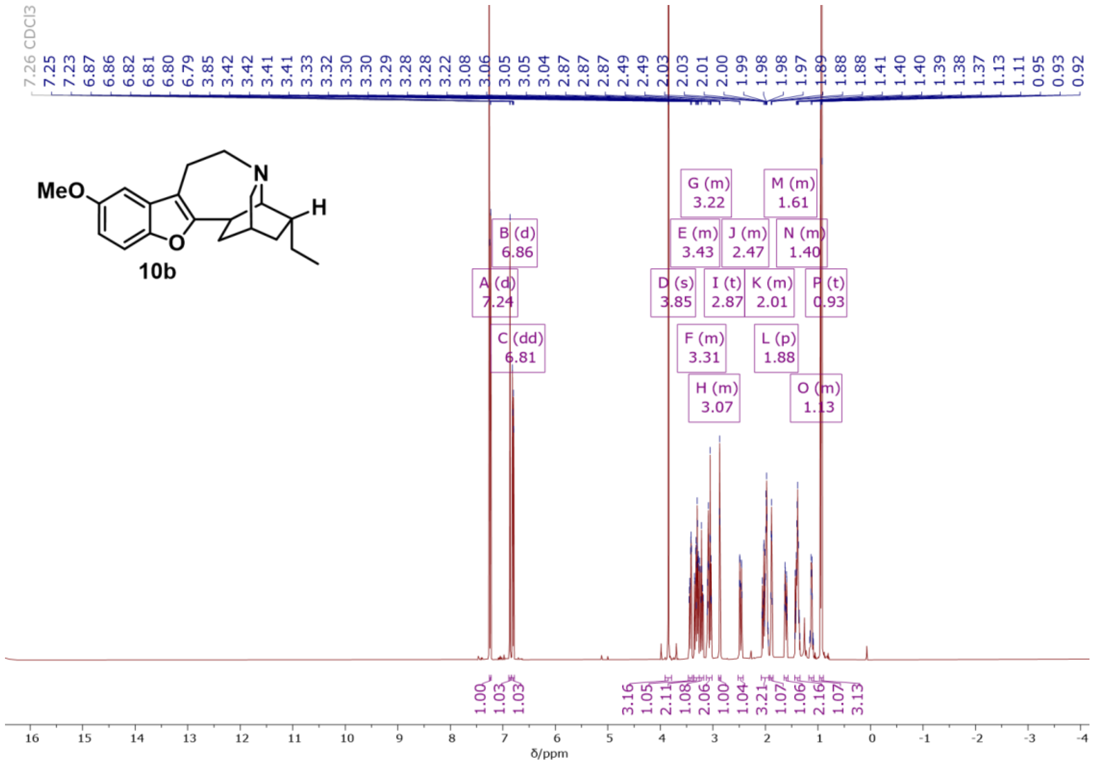

^{1}H NMR (500 MHz, $CDCl_3$) spectrum of compound **10b.**

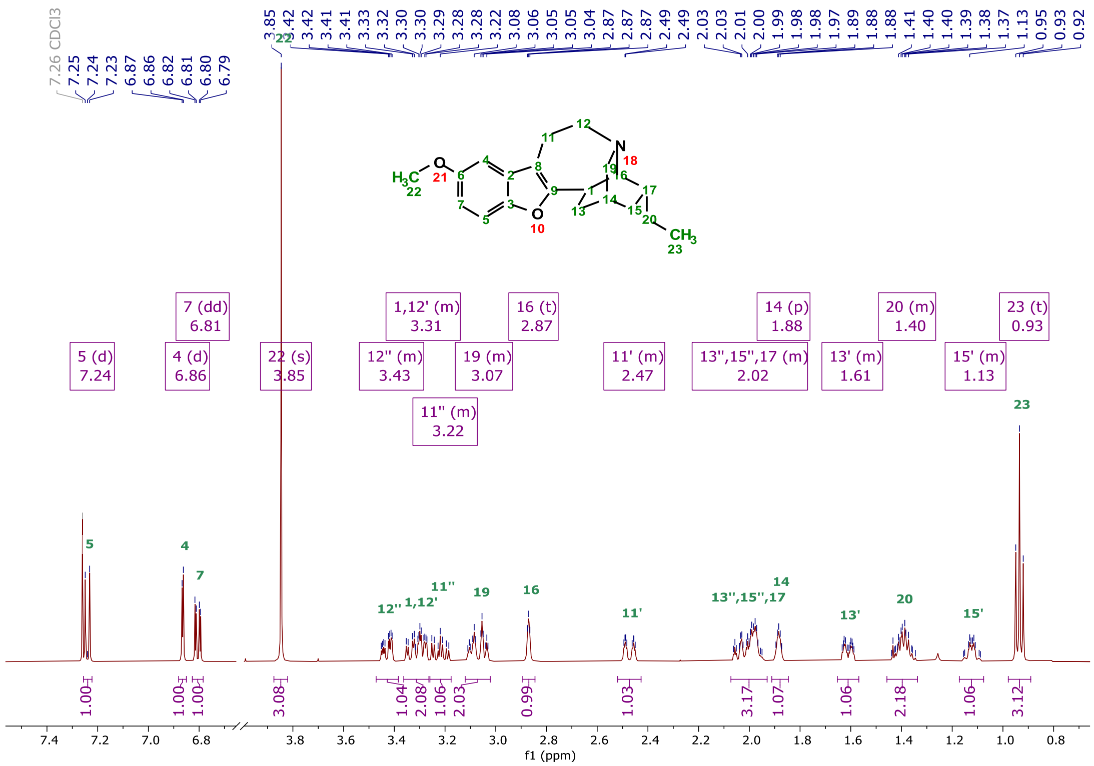

^{1}H NMR (500 MHz, $CDCl_3$) magnified and assigned spectrum of compound **10b**. Blank regions of spectra were omitted for clarity.

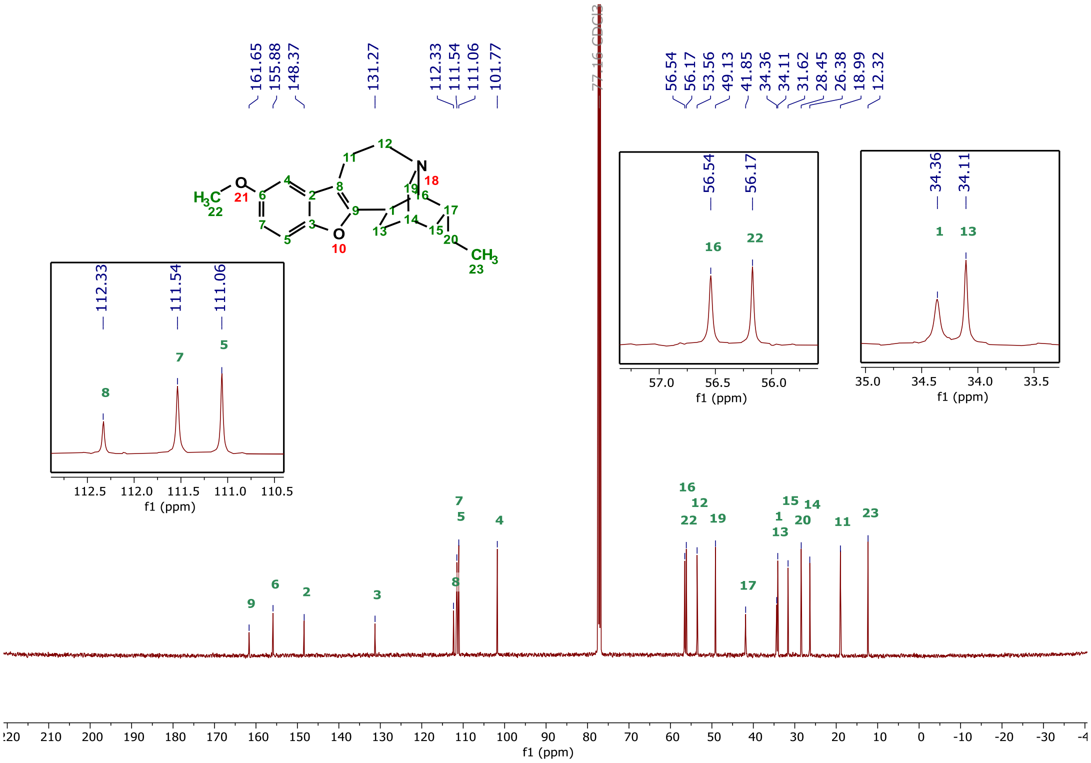

^{13}C NMR (126 MHz, $CDCl_3$) spectrum of compound **10b**. Expansions included for tightly clustered peaks.

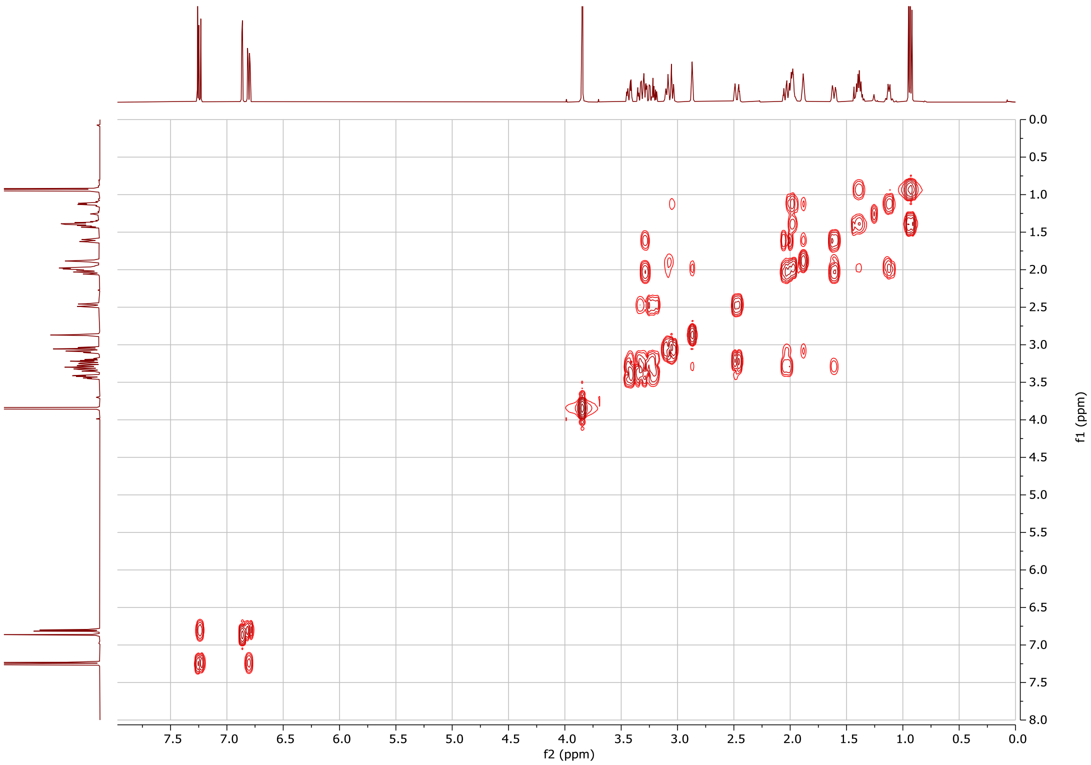

^{1}H-^{1}H COSY NMR (500 MHz, $CDCl_3$) spectrum of compound **10b.**

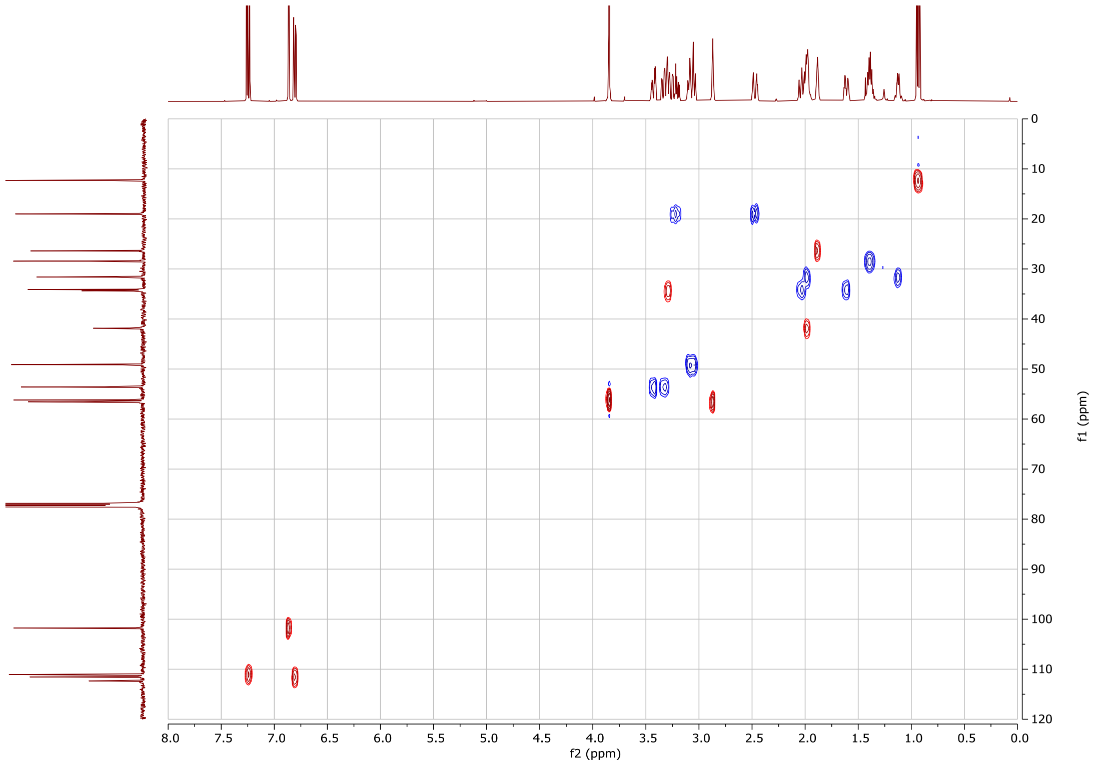

^1H-^{13}C HSQC multiplicity edited NMR (500/126 MHz, $CDCl_3$) spectrum of compound **10b.**

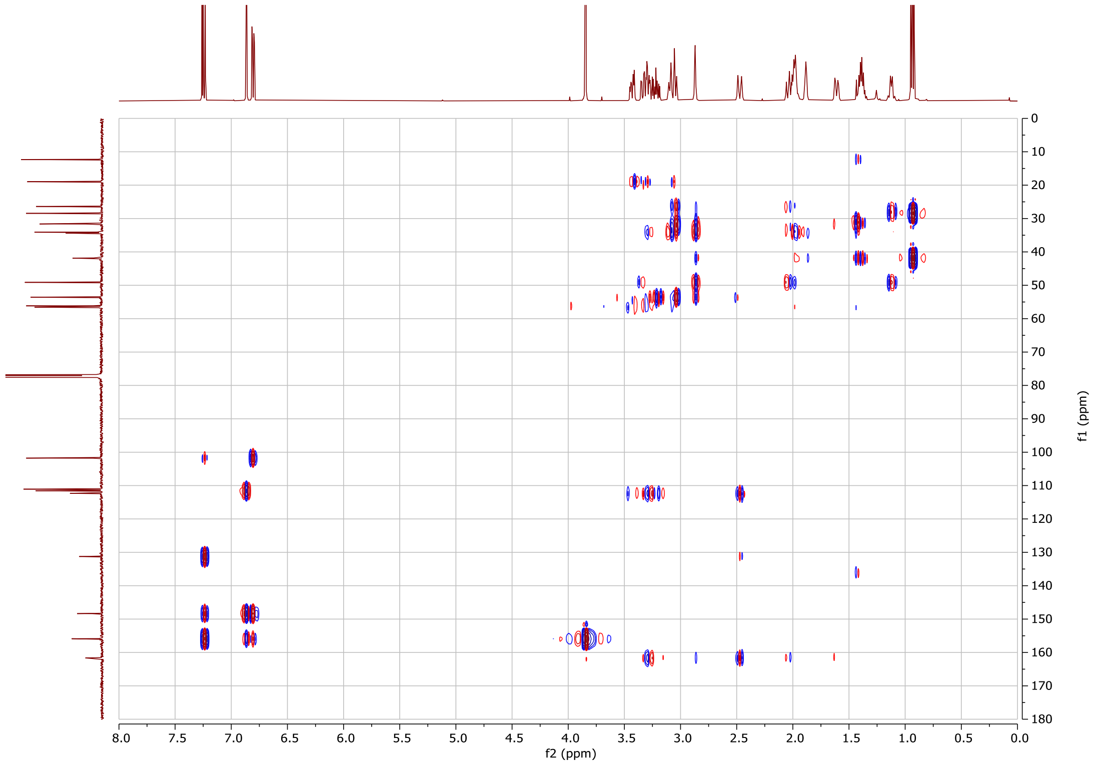

^1H-^{13}C HMBC NMR (500/126 MHz, $CDCl_3$) spectrum of compound **10b**, one-bond correlations are suppressed.

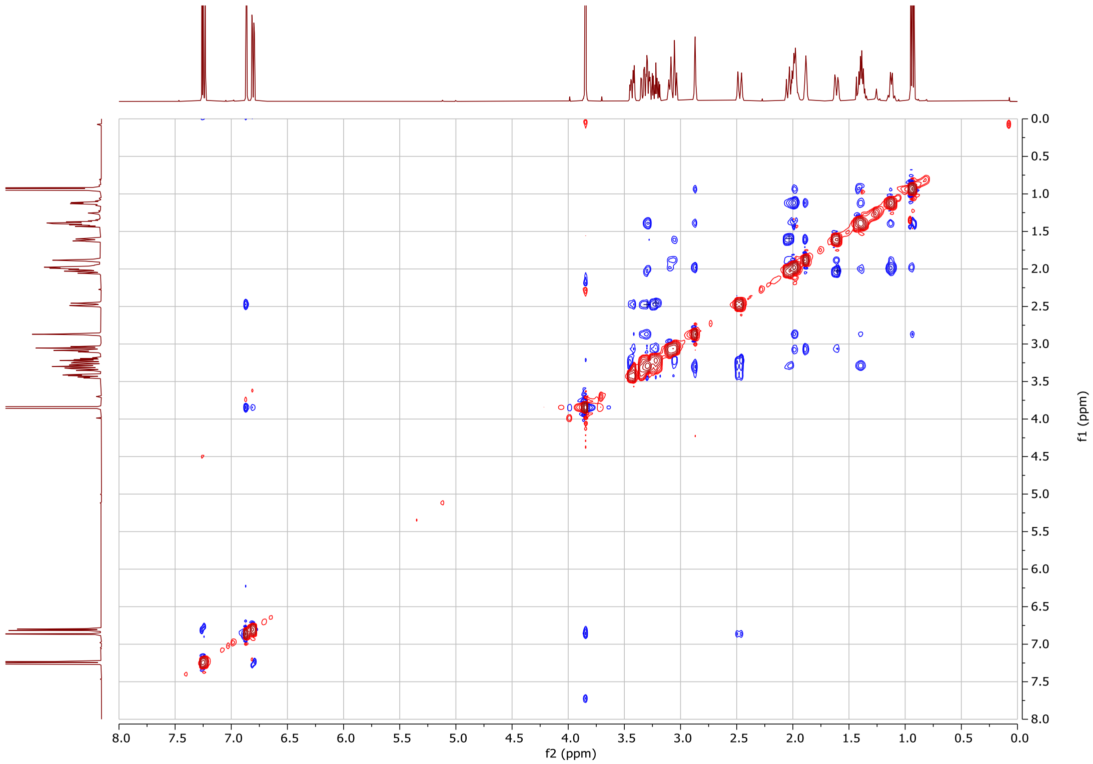

^{1}H-^{1}H NOESY NMR (500 MHz, $CDCl_3$) spectrum of compound **10b.**

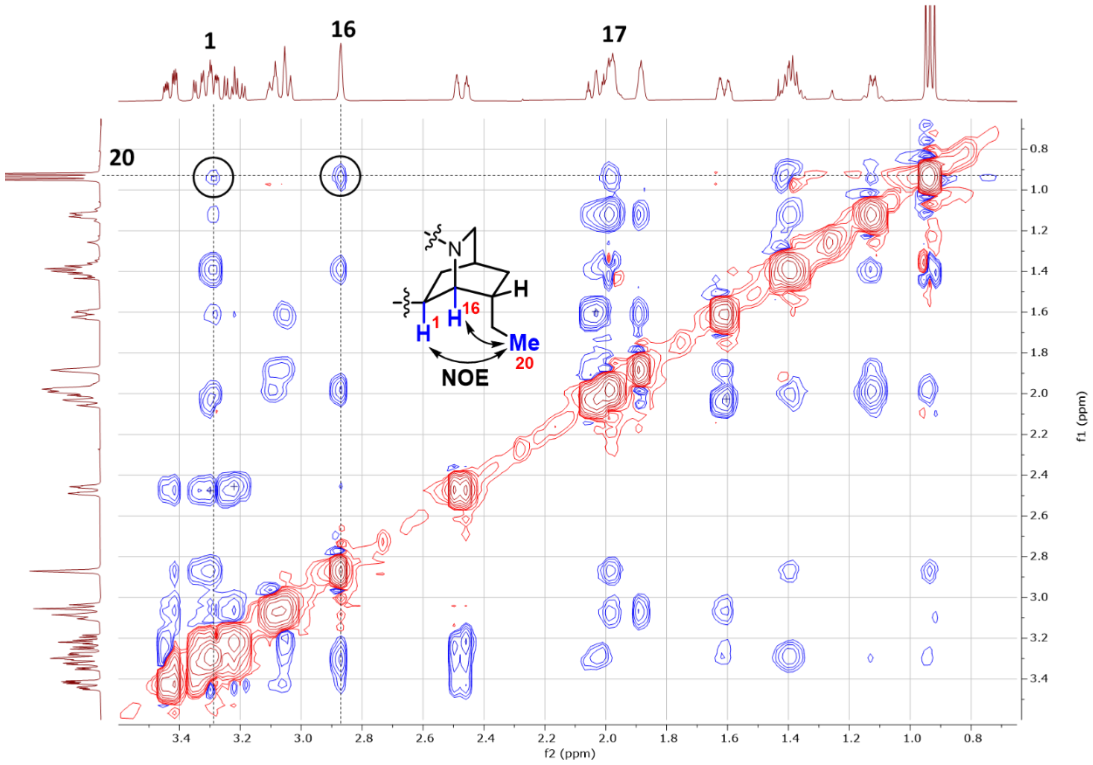

Isoquinuclidine portion of the NOESY spectrum of compound **10b.**

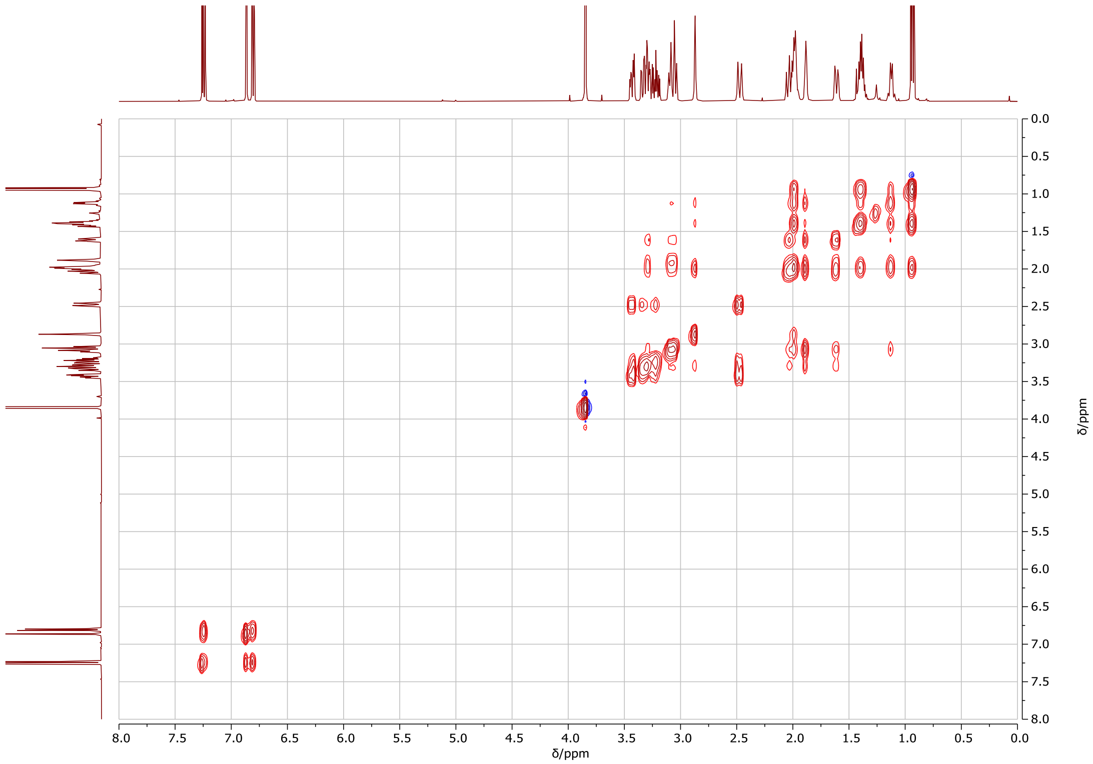

^{1}H-^{1}H TOCSY NMR (500 MHz, $CDCl_3$) spectrum of compound **10b.**

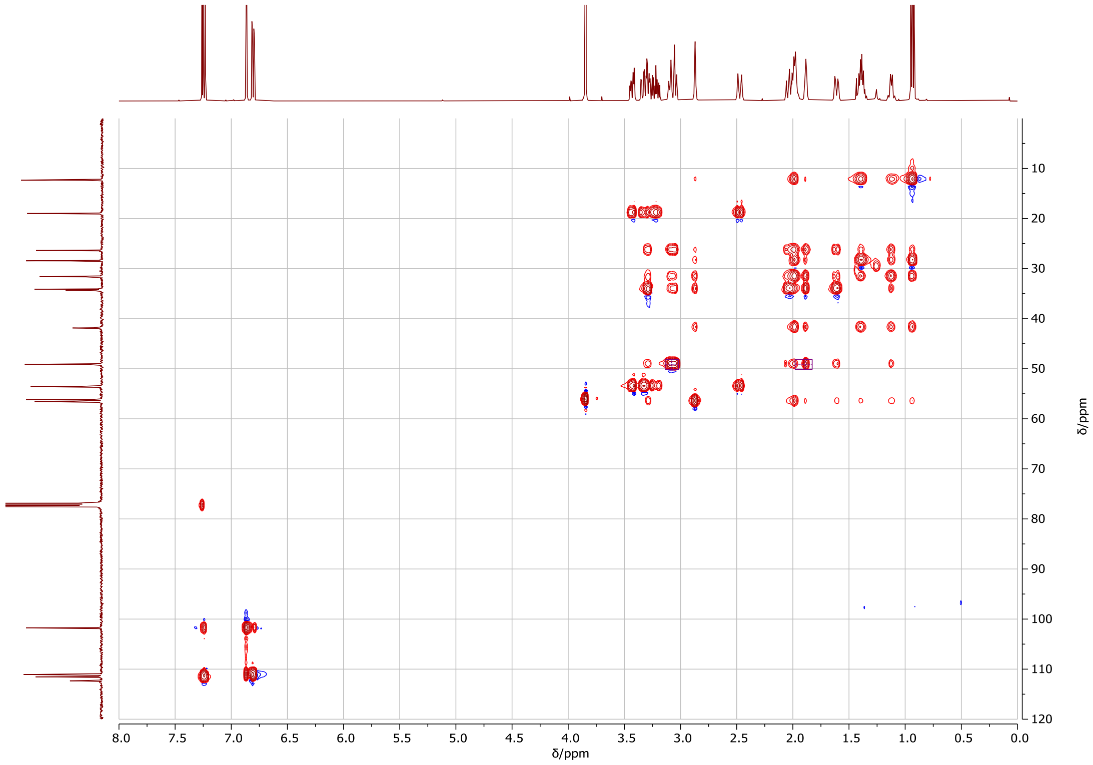

^{1}H-^{13}C HSQC-TOCSY NMR (500/126 MHz, $CDCl_3$) spectrum of compound **10b**.

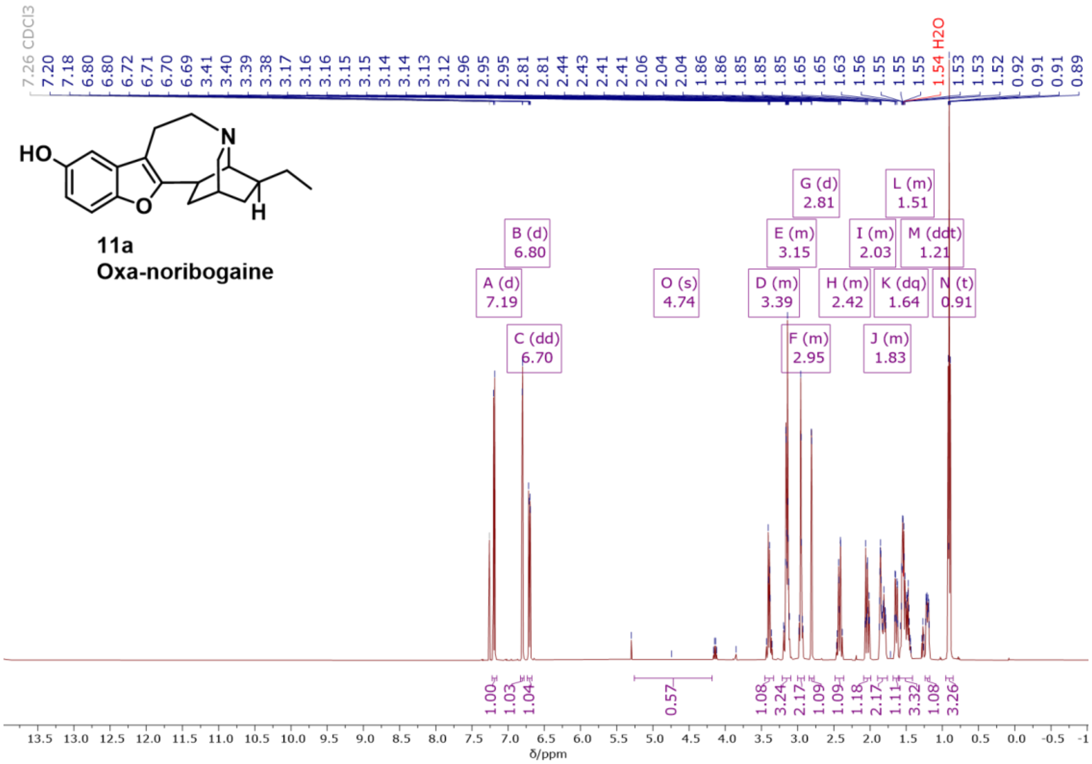

^{1}H NMR (500 MHz, $CDCl_3$) spectrum of compound **11a.**

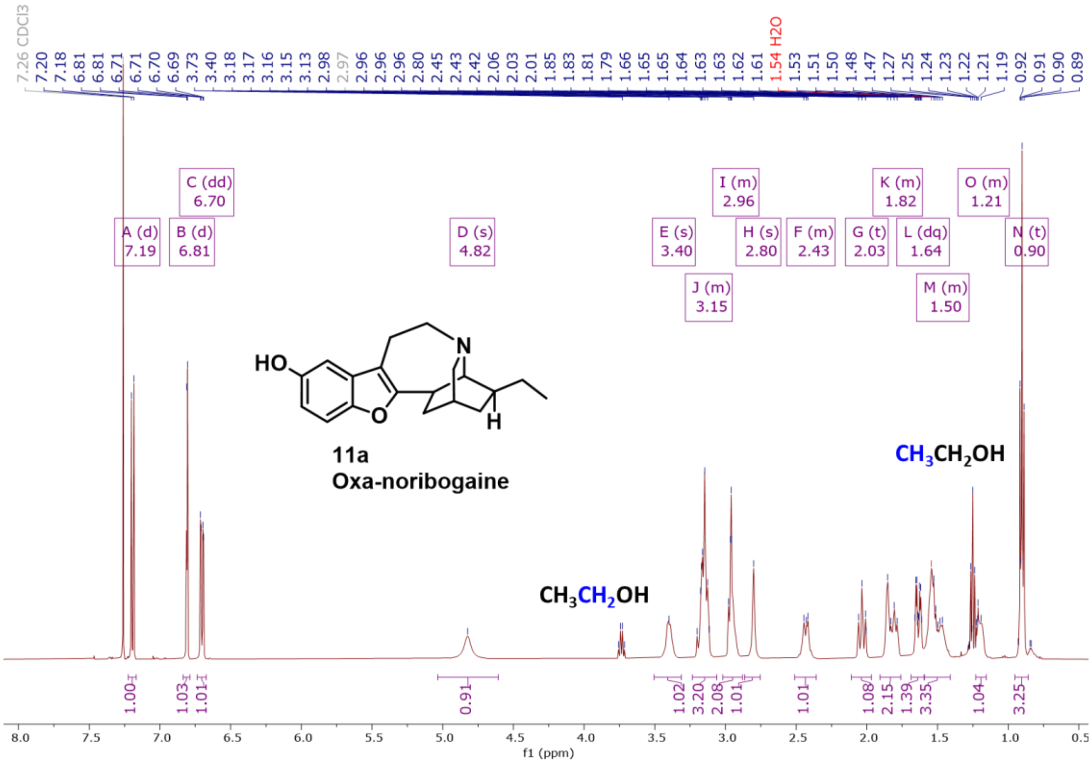

^{1}H NMR (500 MHz, $CDCl_3$) spectrum of compound **11a**, multigram scale preparation. Residual solvent (EtOH) remains trapped in solid material even after extensive drying at elevated temperature (45 °C) in vacuum.

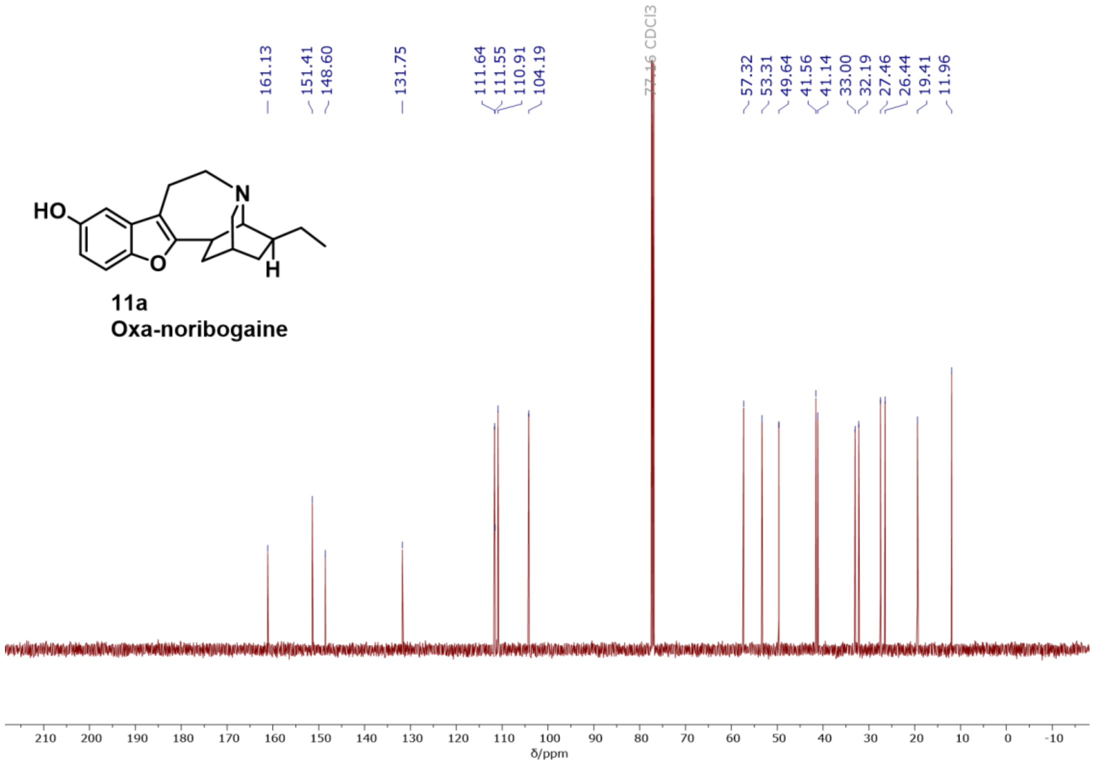

^{13}C NMR (126 MHz, $CDCl_3$) spectrum of compound **11a**.

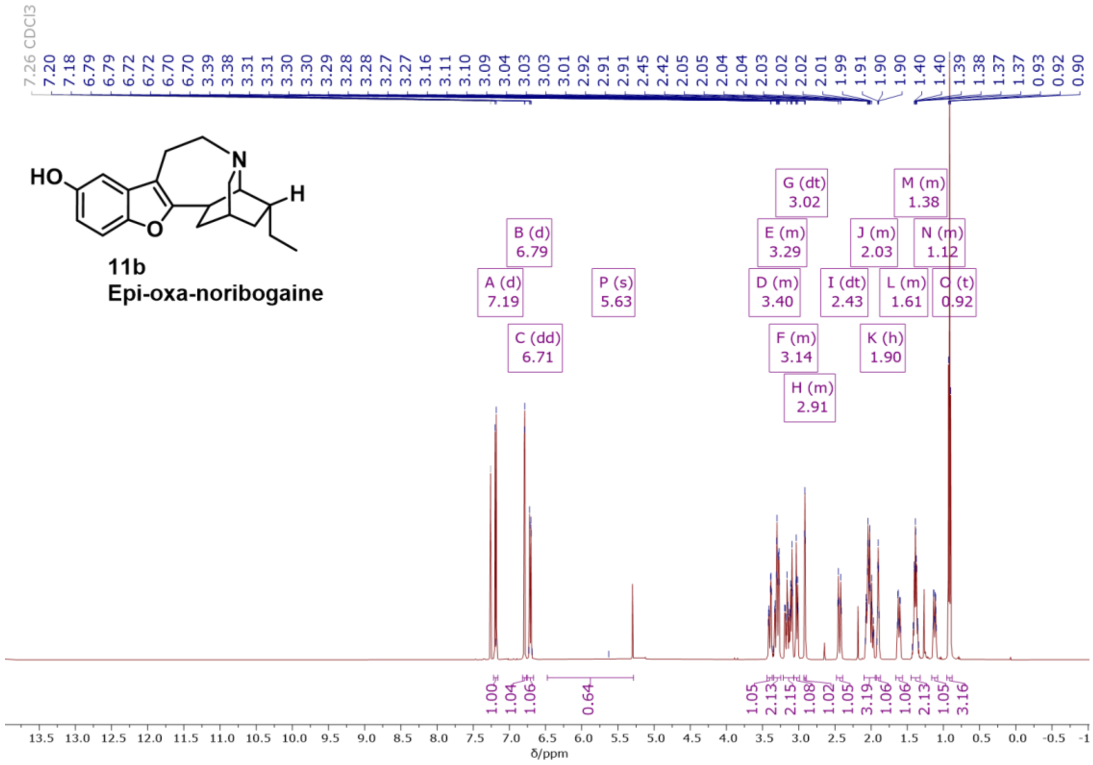

^{1}H NMR (500 MHz, $CDCl_3$) spectrum of compound **11b.**

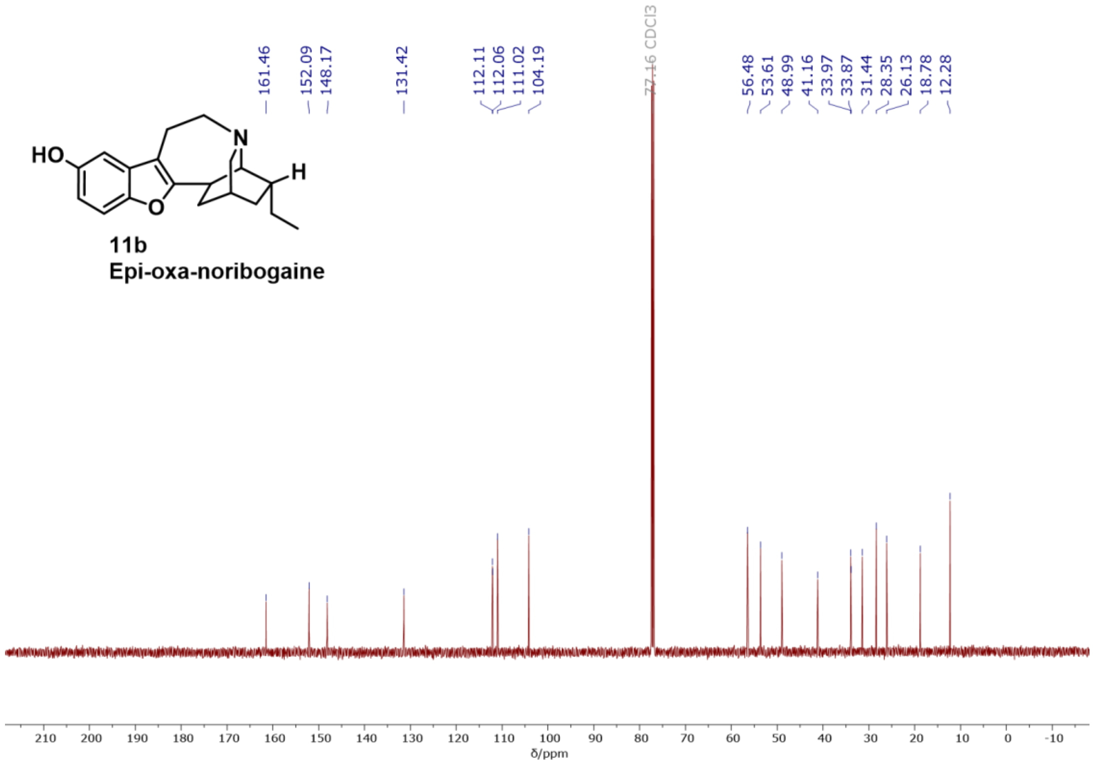

^{13}C NMR (126 MHz, $CDCl_3$) spectrum of compound **11b**.

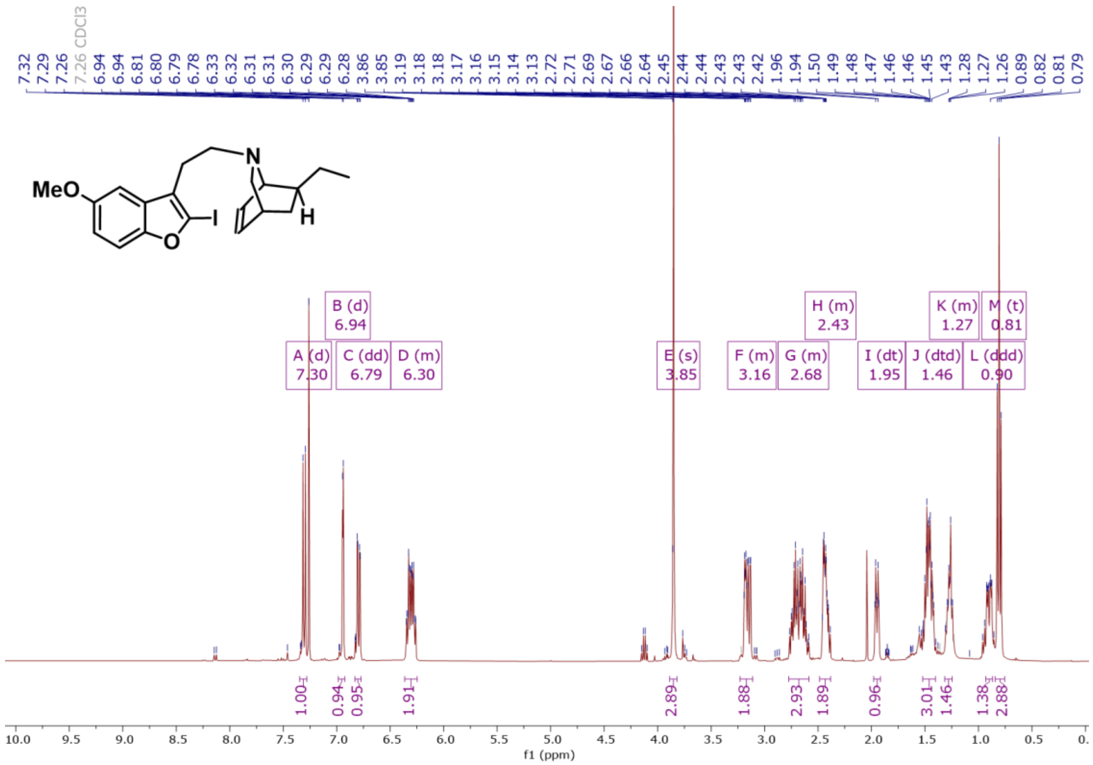

^{1}H NMR (400 MHz, $CDCl_3$) spectrum of crude 2-iodo-benzofuran-*exo*-ethyl-isoquinuclidine intermediate**.**

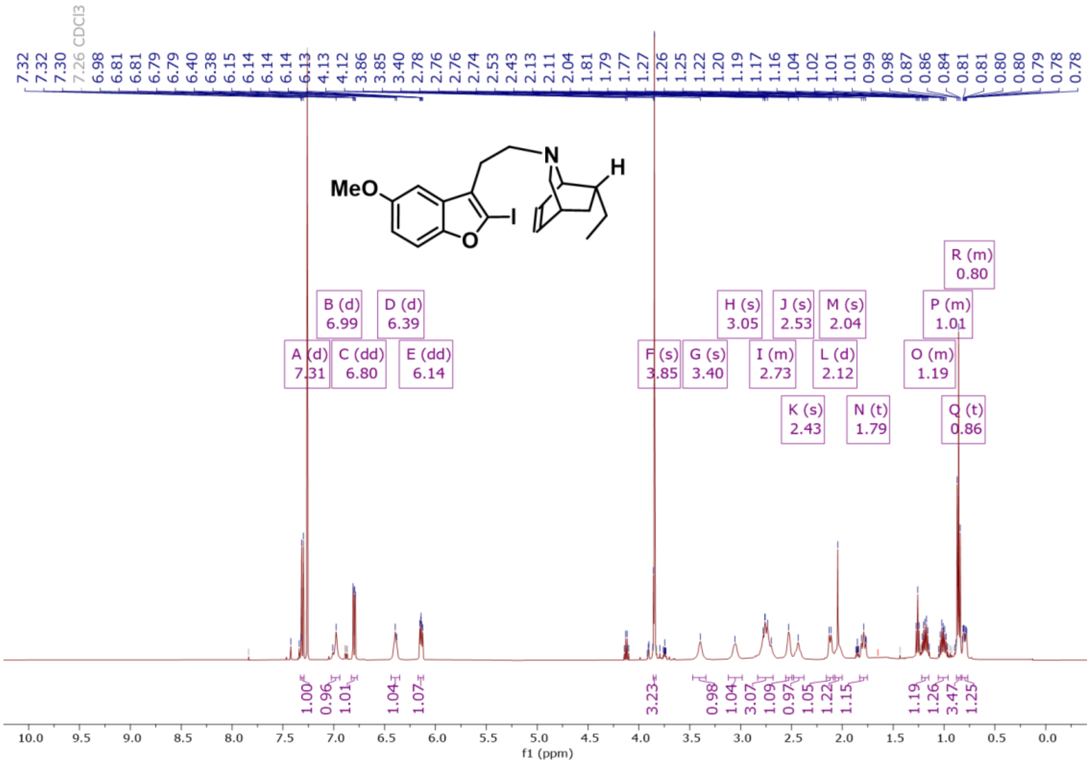

^{1}H NMR (400 MHz, $CDCl_3$) spectrum of crude 2-iodo-benzofuran-*endo*-ethyl-isoquinuclidine intermediate**.**

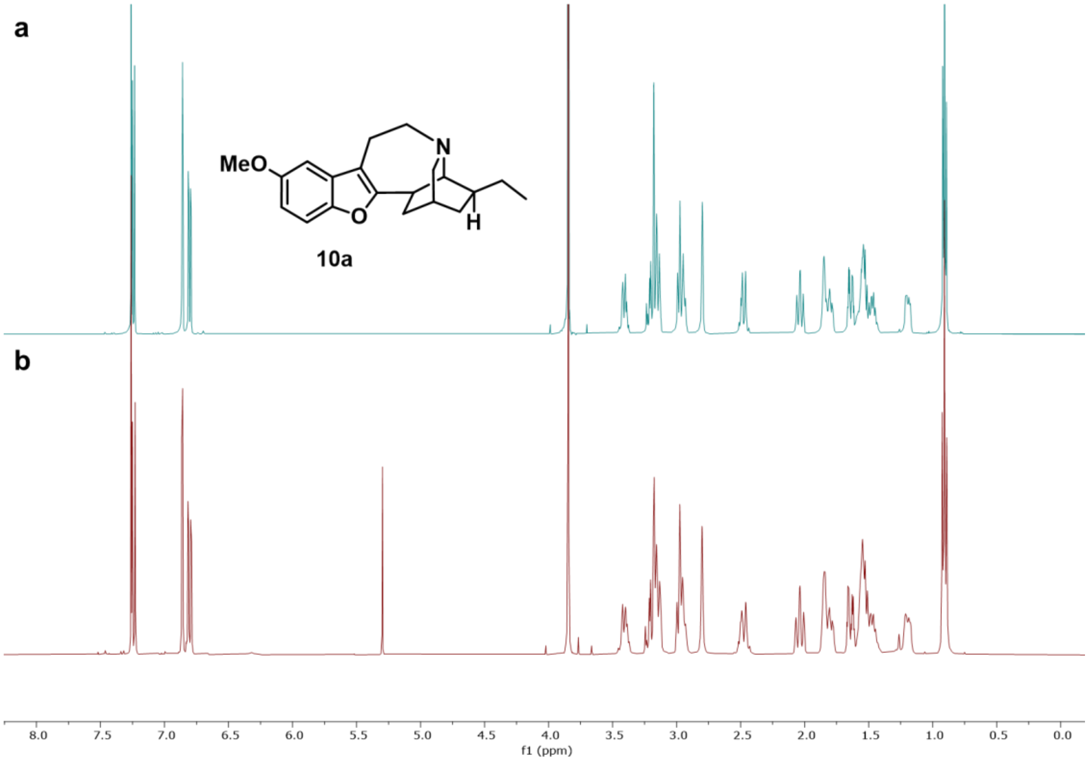

Stacked ^{1}H NMR spectra of oxa-ibogaine **10a** prepared using a) Nickel mediated C-H coupling or b) reductive Heck procedure**.**

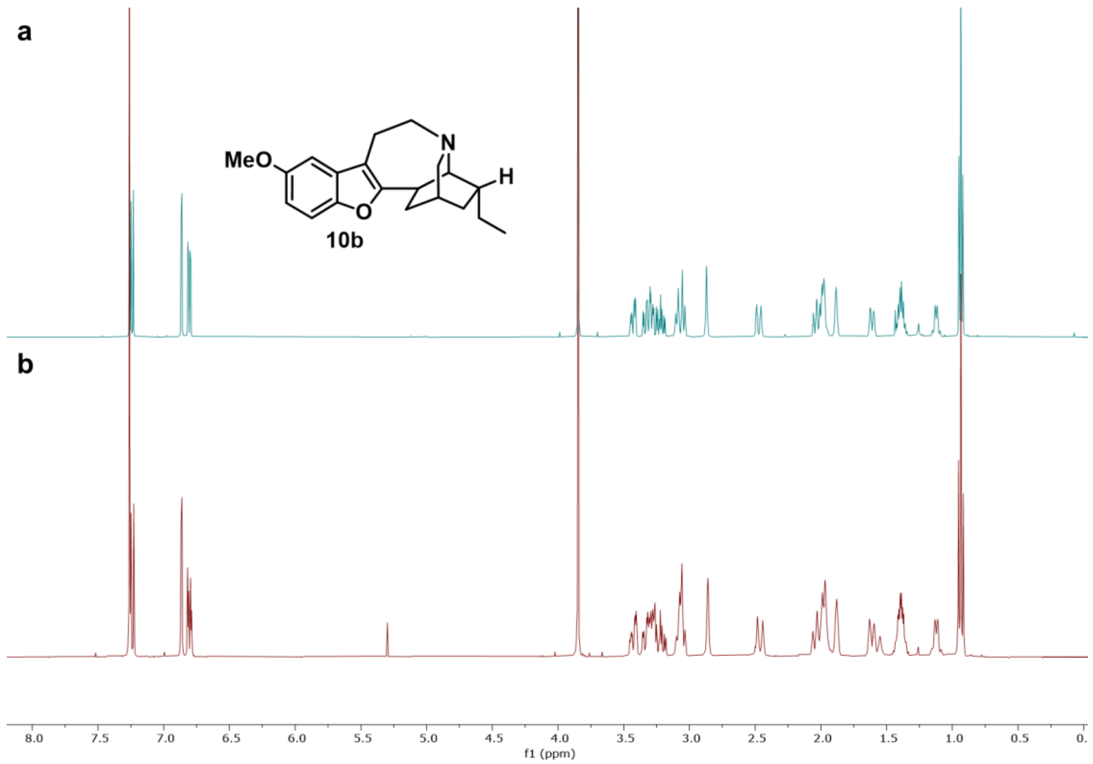

Stacked ^{1}H NMR spectra of epi-oxa-ibogaine **10b** prepared using a) Nickel mediated C-H coupling or b) reductive Heck procedure**.**

Supplementary References

1. Rodríguez, P. *et al.* A Single Administration of the Atypical Psychedelic Ibogaine or Its Metabolite Noribogaine Induces an Antidepressant-Like Effect in Rats. *ACS Chem. Neurosci.* **11**, 1661–1672 (2020).
2. González, B. *et al.* Efficient Access to the Iboga Skeleton: Optimized Procedure to Obtain Voacangine from Voacanga africana Root Bark. *ACS Omega* **6**, 16755–16762 (2021).
3. Fowler, F. W. Synthesis of 1,2- and 1,4-dihydropyridines. *J. Org. Chem.* **37**, 1321–1323 (1972).
4. Mariano, P. S., Dunaway-Mariano, D. & Huesmann, P. L. Amino-Claisen rearrangements of N-vinylisoquinuclidines in novel approaches to the synthesis of hydroisoquinolines and hydrophenanthridines. *J. Org. Chem.* **44**, 124–133 (1979).
5. Hodgson, D. M. & Galano, J.-M. Enantioselective Access to Isoquinuclidines by Tropenone Desymmetrization and Homoallylic Radical Rearrangement: Synthesis of (+)-Ibogamine. *Org. Lett.* **7**, 2221–2224 (2005).
6. Hammond, M. L. *et al.* Antioxidant-based inhibitors of leukotriene biosynthesis. The discovery of 6-[1-[2-(hydroxymethyl)phenyl]-1-propen-3-yl]-2,3-dihydro-5- benzofuranol, a potent topical antiinflammatory agent. *J. Med. Chem.* **33**, 908–918 (1990).
7. Kimura, H., Sato, H., Tsuchiya, C., Chiba, T. & Kato, T. Reaction of 4-haloacetoacetate with phenols in the presence of aluminum chloride. *Chem. Pharm. Bull. (Tokyo)* **30**, 552–558 (1982).
8. Gronauer, T. F. *et al.* Design and synthesis of tailored human caseinolytic protease P inhibitors. *Chem. Commun.* **54**, 9833–9836 (2018).
9. Tomaszewski, Z., Johnson, M. P., Huang, X. & Nichols, D. E. Benzofuran bioisosteres of hallucinogenic tryptamines. *J. Med. Chem.* **35**, 2061–2064 (1992).
10. Porcu, S. *et al.* Brønsted Acid Mediated Cascade Reaction To Access 3-(2-Bromoethyl)benzofurans. *Org. Lett.* **20**, 7699–7702 (2018).
11. Kruegel, A. C., Rakshit, S., Li, X. & Sames, D. Constructing *Iboga* Alkaloids via C–H Bond Functionalization: Examination of the Direct and Catalytic Union of Heteroarenes and Isoquinuclidine Alkenes. *J. Org. Chem.* **80**, 2062–2071 (2015).
12. Paul, S., Pattanayak, S. & Sinha, S. Synthesis of new series of iboga analogues. *Tetrahedron Lett.* **52**, 6166–6169 (2011).
13. Jana, G. K. & Sinha, S. Reductive Heck coupling: an efficient approach toward the iboga alkaloids. Synthesis of ibogamine, epiibogamine and iboga analogs. *Tetrahedron Lett.* **53**, 1671–1674 (2012).
