# Supplementary material for: Oxa-Iboga alkaloids lack cardiac risk and disrupt opioid use in animal models
Source: Nat Commun. 2024 Sep 20;15:8118. doi: 10.1038/s41467-024-51856-y (PMC11415492; doi:10.1038/s41467-024-51856-y)
Supplement: Supplementary file 3 — Description of Additional Supplementary Files [file 41467_2024_51856_MOESM3_ESM.pdf]

Description of Additional Supplementary Files

Supplementary Data 1 - Oxa-ibogaine **10a** X-ray structure (.cif file)

Crystal structure of oxa-ibogaine **10a**, presented on Supplementary Figure 2c.

Supplementary Data 2 - Oxa-ibogaine **10a** X-ray structure report (checkcif file)

CheckCIF file documenting the consistency and integrity of the reported crystal structure determination.

Supplementary Data 3 - Docking structure: noribogaine (PDB file)

Optimized conformation of noribogaine docked inside the active state kappa opioid receptor (KOR), presented on Figure 1d and Supplementary Figure 4e and 4g.

Supplementary Data 4 - Docking structure: oxa-noribogaine (PDB file)

Optimized conformation of oxa-noribogaine **11a** docked inside the active state kappa opioid receptor (KOR), presented on Supplementary Figure 4f and 4g.
