# Supplementary material for: Oxa-Iboga alkaloids lack cardiac risk and disrupt opioid use in animal models
Source: Nat Commun. 2024 Sep 20;15:8118. doi: 10.1038/s41467-024-51856-y (PMC11415492; doi:10.1038/s41467-024-51856-y)

checkCIF/PLATON report

Structure factors have been supplied for datablock(s) vh513

THIS REPORT IS FOR GUIDANCE ONLY. IF USED AS PART OF A REVIEW PROCEDURE FOR PUBLICATION, IT SHOULD NOT REPLACE THE EXPERTISE OF AN EXPERIENCED CRYSTALLOGRAPHIC REFEREE.

No syntax errors found. CIF dictionary Interpreting this report

Datablock: vh513

```
Bond precision:  C-C = 0.0016 A          Wavelength=0.71073

Cell:            a=10.9618(18)     b=8.4800(14)       c=17.660(3)
                 alpha=90          beta=97.742(3)     gamma=90
Temperature:     150 K

                Calculated                 Reported
Volume          1626.6(5)                  1626.6(5)
Space group     P 21/n                     P 21/n
Hall group      -P 2yn                     -P 2yn
Moiety formula  C20 H25 N O2               ?
Sum formula     C20 H25 N O2               C20 H25 N O2
Mr              311.41                     311.41
Dx,g cm-3       1.272                      1.272
Z               4                          4
Mu (mm-1)       0.081                      0.081
F000            672.0                      672.0
F000'           672.28
h,k,lmax        15,12,25                   15,12,25
Nref            4983                       4982
Tmin,Tmax       0.989,0.994                0.678,0.746
Tmin'           0.966

Correction method= # Reported T Limits: Tmin=0.678 Tmax=0.746
AbsCorr = EMPIRICAL

Data completeness= 1.000          Theta(max)= 30.566

R(reflections)= 0.0441( 3804)     wR2(reflections)= 0.1217( 4982)

S = 1.045                   Npar= 210
```

The following ALERTS were generated. Each ALERT has the format
test-name_ALERT_alert-type_alert-level.
Click on the hyperlinks for more details of the test.

Alert level C

```
PLAT094_ALERT_2_C Ratio of Maximum / Minimum Residual Density ....       2.06 Report
```

Alert level G

```
PLAT398_ALERT_2_G Deviating  C-O-C    Angle From 120 for O1            105.9 Degree
PLAT793_ALERT_4_G Model has Chirality at C12         (Centro SPGR)         R Verify
PLAT793_ALERT_4_G Model has Chirality at C14         (Centro SPGR)         R Verify
PLAT793_ALERT_4_G Model has Chirality at C18         (Centro SPGR)         S Verify
PLAT793_ALERT_4_G Model has Chirality at C19         (Centro SPGR)         S Verify
PLAT883_ALERT_1_G No Info/Value for _atom_sites_solution_primary .     Please Do !
PLAT912_ALERT_4_G Missing # of FCF Reflections Above STh/L=  0.600         2 Note
PLAT978_ALERT_2_G Number C-C Bonds with Positive Residual Density.        15 Info
PLAT992_ALERT_5_G Repd & Actual _reflns_number_gt Values Differ by         1 Check
```

```
   0 ALERT level A = Most likely a serious problem - resolve or explain
   0 ALERT level B = A potentially serious problem, consider carefully
   1 ALERT level C = Check. Ensure it is not caused by an omission or oversight
   9 ALERT level G = General information/check it is not something unexpected

   1 ALERT type 1 CIF construction/syntax error, inconsistent or missing data
   3 ALERT type 2 Indicator that the structure model may be wrong or deficient
   0 ALERT type 3 Indicator that the structure quality may be low
   5 ALERT type 4 Improvement, methodology, query or suggestion
   1 ALERT type 5 Informative message, check
```

It is advisable to attempt to resolve as many as possible of the alerts in all categories. Often the minor alerts point to easily fixed oversights, errors and omissions in your CIF or refinement strategy, so attention to these fine details can be worthwhile. In order to resolve some of the more serious problems it may be necessary to carry out additional measurements or structure refinements. However, the purpose of your study may justify the reported deviations and the more serious of these should normally be commented upon in the discussion or experimental section of a paper or in the "special_details" fields of the CIF. checkCIF was carefully designed to identify outliers and unusual parameters, but every test has its limitations and alerts that are not important in a particular case may appear. Conversely, the absence of alerts does not guarantee there are no aspects of the results needing attention. It is up to the individual to critically assess their own results and, if necessary, seek expert advice.

Publication of your CIF in IUCr journals

A basic structural check has been run on your CIF. These basic checks will be run on all CIFs submitted for publication in IUCr journals (*Acta Crystallographica*, *Journal of Applied Crystallography*, *Journal of Synchrotron Radiation*); however, if you intend to submit to *Acta Crystallographica Section C* or *E* or *IUCrData*, you should make sure that full publication checks are run on the final version of your CIF prior to submission.

Publication of your CIF in other journals

Please refer to the *Notes for Authors* of the relevant journal for any special instructions relating to CIF submission.

PLATON version of 22/12/2019; check.def file version of 13/12/2019

Datablock vh513 - ellipsoid plot

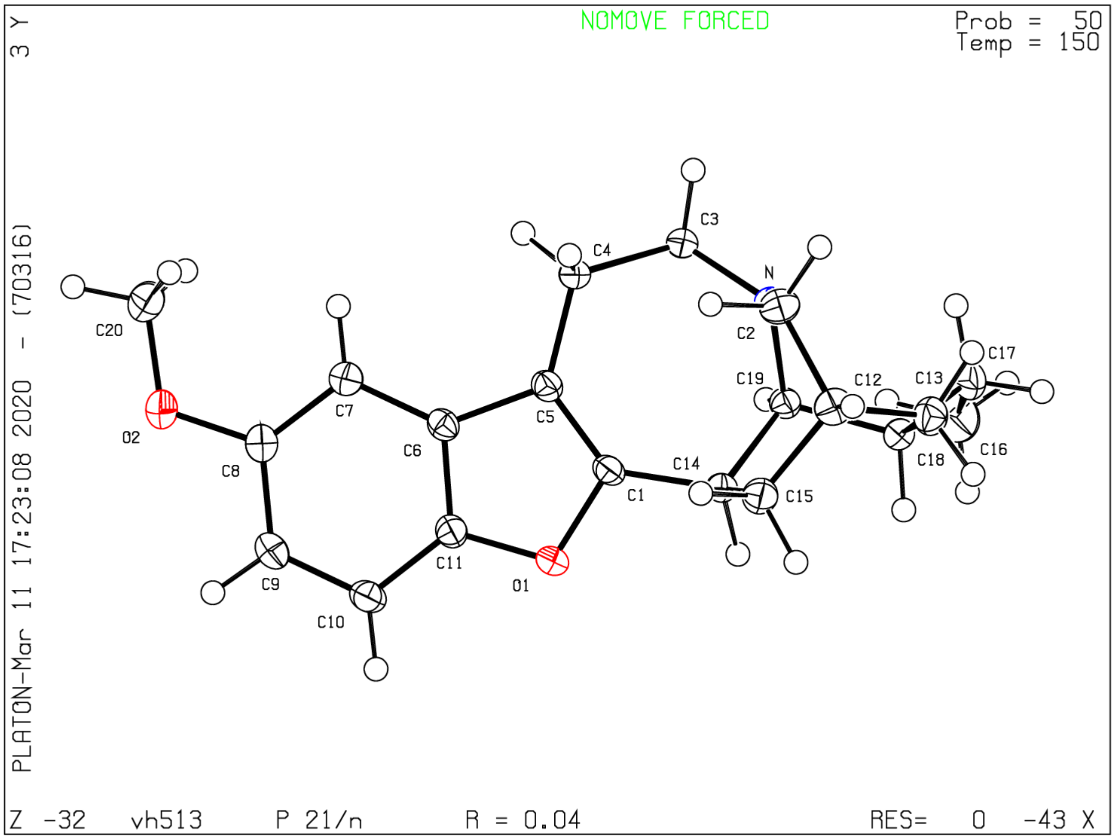

Supplement: Supplementary file 5 — Supplementary Dataset 2 [file 41467_2024_51856_MOESM5_ESM.pdf]
