# Supplementary material for: Oxa-Iboga alkaloids lack cardiac risk and disrupt opioid use in animal models
Source: Nat Commun. 2024 Sep 20;15:8118. doi: 10.1038/s41467-024-51856-y (PMC11415492; doi:10.1038/s41467-024-51856-y)
Supplement: Supplementary file 8 — Reporting Summary [file 41467_2024_51856_MOESM8_ESM.pdf]

nature portfolio

Corresponding author(s): Dalibor Sames

Last updated by author(s): Aug 7, 2024

Reporting Summary

Nature Portfolio wishes to improve the reproducibility of the work that we publish. This form provides structure for consistency and transparency in reporting. For further information on Nature Portfolio policies, see our Editorial Policies and the Editorial Policy Checklist.

Statistics

For all statistical analyses, confirm that the following items are present in the figure legend, table legend, main text, or Methods section.

n/a	Confirmed	
☐	☒	The exact sample size (*n*) for each experimental group/condition, given as a discrete number and unit of measurement
☐	☒	A statement on whether measurements were taken from distinct samples or whether the same sample was measured repeatedly
☐	☒	The statistical test(s) used AND whether they are one- or two-sided<br>*Only common tests should be described solely by name; describe more complex techniques in the Methods section.*
☒	☐	A description of all covariates tested
☐	☒	A description of any assumptions or corrections, such as tests of normality and adjustment for multiple comparisons
☐	☒	A full description of the statistical parameters including central tendency (e.g. means) or other basic estimates (e.g. regression coefficient) AND variation (e.g. standard deviation) or associated estimates of uncertainty (e.g. confidence intervals)
☐	☒	For null hypothesis testing, the test statistic (e.g. *F*, *t*, *r*) with confidence intervals, effect sizes, degrees of freedom and *P* value noted<br>*Give P values as exact values whenever suitable.*
☒	☐	For Bayesian analysis, information on the choice of priors and Markov chain Monte Carlo settings
☒	☐	For hierarchical and complex designs, identification of the appropriate level for tests and full reporting of outcomes
☒	☐	Estimates of effect sizes (e.g. Cohen's *d*, Pearson's *r*), indicating how they were calculated

Our web collection on statistics for biologists contains articles on many of the points above.

Software and code

Policy information about availability of computer code

Data collection	Nuclear magnetic resonance spectra were collected using Bruker TopSpin (3.5 pl6) software. Control and data collecting software appropriate for each instrument mentioned in the Methods section was used: BioTek H1MF and PHERAstar FS plate readers; IonOptix and MyoBLAZER microscopes and cameras; Ugo Basile unit and IITC Model 33 Tail Flick Analgesia Meters; Activity Monitor by Med Associates; Noldus FST scoring software; CPP/CPA and SA studies (Med Associates instruments and Micro Interfaces MCS system); Pharmacokinetic study (LC-MS/MS Waters and Applied Biosystems instruments); ICM-Pro v3.9 molecular modeling and drug discovery suite (Molsoft LLC);
Data analysis	Nuclear magnetic resonance spectra were processed using MestReNova 14. Non-linear curve fitting and statistical analysis was performed using mathematical models supplied with GraphPad Prism (Versions 8, 9 and 10). Cardiotoxicity data were analyzed using IonWizard and MyoBLAZER software. Forced swim test experiments were video recorded and automatically scored (Noldus software). Non-Compartmental-Analysis tool of Phoenix WinNonlin® (Version 8.0 for oxa-noribogaine, Version 7.0 for noribogaine) was used to assess the pharmacokinetic parameters.<br>Additional software used: Microsoft Office 365; ChemOffice 2020; PyMOL 2.3.1; BioRender (https://biorender.com);

For manuscripts utilizing custom algorithms or software that are central to the research but not yet described in published literature, software must be made available to editors and reviewers. We strongly encourage code deposition in a community repository (e.g. GitHub). See the Nature Portfolio guidelines for submitting code & software for further information.

Data

Policy information about availability of data

All manuscripts must include a data availability statement. This statement should provide the following information, where applicable:

- Accession codes, unique identifiers, or web links for publicly available datasets
- A description of any restrictions on data availability
- For clinical datasets or third party data, please ensure that the statement adheres to our policy

The crystallographic data (oxa-ibogaine 10a) generated in this study have been deposited in the Cambridge Crystallographic Data Centre database under accession code #2215015 www.ccdc.cam.ac.uk/structures. The .cif file was provided together with the docking structures as supplementary data.
The data generated in this study are provided in the Supplementary Information and Source Data file (Figures 2-6 and Supplementary Figures 3-9).

Research involving human participants, their data, or biological material

Policy information about studies with human participants or human data. See also policy information about sex, gender (identity/presentation), and sexual orientation and race, ethnicity and racism.

Reporting on sex and gender	Only tissue material originating from deceased adult male donor hearts was used within the study.
Reporting on race, ethnicity, or other socially relevant groupings	Donors were reported to be of Hispanic or Caucasian ethnicity.
Population characteristics	Age and sex are provided for each donor. Remaining indirect identifiers: ethnicity, body mass index, cause of death and ejection fraction are provided in aggregate.
Recruitment	Donor characteristics are reported on (Figure S8F) and exclusion criteria were previously described. Page, G. et al. Human ex-vivo action potential model for pro-arrhythmia risk assessment. Journal of Pharmacological and Toxicological Methods 81, 183–195 (2016).
Ethics oversight	All human hearts used for the present study were not suitable for transplantation and originated solely from deceased organ donors in the United States (US). Research was carried out by investigators not involved in donor recruitment and all human originating material was received de-identified. As such the study is classified as non-human subjects research (NHR) and does not require prior Institutional Review Board (IRB) approval. Written legal, study nonspecific consent was obtained by the Organ Procurement Organizations (OPOs) from the donor or next of kin wishes. No compensation was provided for the organ donations.<br><br>AnaBios Corporation's procurement network includes only US based Organ Procurement Organizations (OPOs) and Hospitals. Policies for donor screening and consent are the ones established by the United Network for Organ Sharing (UNOS). Organizations supplying human tissues to AnaBios follow the standards and procedures established by the US Centers for Disease Control (CDC), are inspected biannually by the Department of Health and Human Services (DHHS), are certified by Centers for Medicare & Medicaid Services (CMS) and must abide by CMS regulations. Tissue distribution by OPOs is governed by their internal Institutional Review Board (IRB) procedures in compliance with Health Insurance Portability and Accountability Act (HIPAA) regulations regarding patient privacy. All transfers of donor organs to AnaBios are fully traceable and periodically reviewed by US Federal authorities.

Note that full information on the approval of the study protocol must also be provided in the manuscript.

Field-specific reporting

Please select the one below that is the best fit for your research. If you are not sure, read the appropriate sections before making your selection.

☒ Life sciences ☐ Behavioural & social sciences ☐ Ecological, evolutionary & environmental sciences

For a reference copy of the document with all sections, see nature.com/documents/nr-reporting-summary-flat.pdf

Life sciences study design

All studies must disclose on these points even when the disclosure is negative.

Sample size	Number of replicates and statistical analysis are reported in the figure and table legends, in the supplementary information, supplementary statistics table and GraphPad Prism figure files.<br>All comparisons were planned before performing each experiment based on literature reports and/or preliminary experiments. Statistical methods were not used to predetermine sample sizes, with the exception that power analyses were conducted to estimate number of animal subjects required for in vivo experiments.<br>For self-administration studies samples size calculations were used to determine the number of subjects required for $\alpha = 0.05$ and $\beta = 0.9$, assuming variance determined from previous studies for specific inferential statistical analyses.

Data exclusions	Outlier data were not excluded from analysis.<br>Subjects that did not complete the treatment regimens during self-administration studies were excluded from analysis.
Replication	Data were replicated using technical and independent replicates. See figure and table legends, the supplementary information and source data files for specific details.
Randomization	For self-administration studies pseudo-randomization prior to initiation of self-administration training was used.
Blinding	In Vitro pharmacological characterization, MOR/KOR KO and control WT tailf-lick tests, cardiotoxicity study, pharmacokinetic and protein/ tissue binding experiments were performed and analyzed by experimenters blinded to the chemical identity of the test substance.<br>Antagonism tail-flick and openfield experiments were performed unblinded, due to the fact that the specific traits of the antagonists used (pre-administration time, receptor target) needed to be known to correctly perform the experiments.<br>The overt behavioral effects of the test compounds precluded blinding of drugs or doses during self-administration studies.

Reporting for specific materials, systems and methods

We require information from authors about some types of materials, experimental systems and methods used in many studies. Here, indicate whether each material, system or method listed is relevant to your study. If you are not sure if a list item applies to your research, read the appropriate section before selecting a response.

Materials & experimental systems

n/a	Involved in the study
☒	☐ Antibodies
☐	☒ Eukaryotic cell lines
☒	☐ Palaeontology and archaeology
☐	☒ Animals and other organisms
☒	☐ Clinical data
☒	☐ Dual use research of concern
☒	☐ Plants

Methods

n/a	Involved in the study
☒	☐ ChIP-seq
☒	☐ Flow cytometry
☒	☐ MRI-based neuroimaging

Eukaryotic cell lines

Policy information about cell lines and Sex and Gender in Research

Cell line source(s)	HEK293T (CRL-3216) was purchased on from American Type Culture Collection (Rockville, MD, USA).<br>CHO-K1 cells were obtained from the American Type Culture Collection American Type Culture Collection (Rockville, MD, USA).<br>For assays performed by contracted research organizations, cells were procured by the specified service provider.
Authentication	Cells have not been authenticated after purchase.
Mycoplasma contamination	Cells have not been tested for mycoplasma.
Commonly misidentified lines<br>(See ICLAC register)	No commonly misidentified cell lines were used in the study.

Animals and other research organisms

Policy information about studies involving animals; ARRIVE guidelines recommended for reporting animal research, and Sex and Gender in Research

Laboratory animals	Specific information regarding the animal test subjects used (species, strain, gender, age and body weight) are provided in the methods section.<br><br>Tail-flick, open-field and forced swim test: adult male/female mice C57BL/6J (8-15 weeks, 29-35 g; Jackson Laboratory (Bar Harbor, ME).<br>Condition placed preference: adult male/female C57Bl/6 mice (10-15 weeks, 25-32 g; Jackson Laboratory (Bar Harbor, ME).<br>Self-administration studies: adult male Fisher F-344 rats (90-150 days, 230-280 g; Charles River Laboratories, Wilmington, MA).<br>Neurotropic factors expression experiments: adult male Fisher F-344 rats (90-150 days, 230-280 g; Charles River Laboratories, Wilmington, MA).<br>Pharmacokinetic studies: healthy adult male C57BL/6 mice (8-12 weeks old, 18 to 36 g) or healthy male Wistar rats (8-12 weeks old, 250 to 280 g) were procured from Global (India).
Wild animals	No wild animals were used in the course of the study.
Reporting on sex	Both sexes were examined in the following mice experiments: tail-flick test (including Knock-out models), novelty induced locomotion (open field test) and conditioned place preference/aversion. Data are provided disaggregated in the source data file.

	Only male mice were examined in the forced swim test and antagonist pretreatment effect on analgesic activity (tail-flick test). Only male rats were used in the GDNF expression, opioid self-administration, food operant intake, cue-induced reinstatement and opioid induced hyperalgesia studies. Pharmacokinetic distribution was determined only in male rodents. And only male rodent plasma and tissue were used for the protein binding experiments.
Field-collected samples	No field-collected samples were used in the course of the study.
Ethics oversight	All experimental procedures involving animals were approved by the Columbia University, Memorial Sloan Kettering Cancer Center, Rutgers University or High Point University Institutional Animal Care and Use Committee (IACUC) and adhered to principles described in the National Institutes of Health Guide for the Care and Use of Laboratory Animals and abide by the ARRIVE guidelines.

Note that full information on the approval of the study protocol must also be provided in the manuscript.

Plants

Seed stocks	*Report on the source of all seed stocks or other plant material used. If applicable, state the seed stock centre and catalogue number. If plant specimens were collected from the field, describe the collection location, date and sampling procedures.*
Novel plant genotypes	*Describe the methods by which all novel plant genotypes were produced. This includes those generated by transgenic approaches, gene editing, chemical/radiation-based mutagenesis and hybridization. For transgenic lines, describe the transformation method, the number of independent lines analyzed and the generation upon which experiments were performed. For gene-edited lines, describe the editor used, the endogenous sequence targeted for editing, the targeting guide RNA sequence (if applicable) and how the editor was applied.*
Authentication	*Describe any authentication procedures for each seed stock used or novel genotype generated. Describe any experiments used to assess the effect of a mutation and, where applicable, how potential secondary effects (e.g. second site T-DNA insertions, mosiacism, off-target gene editing) were examined.*
